# Goondapyrones A–J: Polyketide α and γ Pyrone Anthelmintics from an Australian Soil-Derived *Streptomyces* sp.

**DOI:** 10.3390/antibiotics13100989

**Published:** 2024-10-18

**Authors:** Shengbin Jin, David F. Bruhn, Cynthia T. Childs, Erica Burkman, Yovany Moreno, Angela A. Salim, Zeinab G. Khalil, Robert J. Capon

**Affiliations:** 1 Institute for Molecular Bioscience, The University of Queensland, St Lucia, QLD 4072, Australia; shengbin.jin@uq.edu.au (S.J.); a.salim@uq.edu.au (A.A.S.); z.khalil@uq.edu.au (Z.G.K.); 2 Boehringer Ingelheim Animal Health, USA Inc., 1730 Olympic Drive, Athens, GA 30601, USA; david.bruhn@boehringer-ingelheim.com (D.F.B.); cynthia.childs@boehringer-ingelheim.com (C.T.C.); erica.burkman@boehringer-ingelheim.com (E.B.); yovany.moreno@boehringer-ingelheim.com (Y.M.)

**Keywords:** anthelmintic, citizen science, *Dirofilaria immitis*, *Haemonchus contortus*, microbial natural product, polyketide, pyrones, Soils for Science, *Streptomyces*

## Abstract

An investigation of ×19 soil samples collected under the auspices of the Australian citizen science initiative, Soils for Science, returned ×559 chemically dereplicated microbial isolates, of which ×54 exhibited noteworthy anthelmintic activity against either the heartworm *Dirofilaria immitis* microfilaria and/or the gastrointestinal parasite *Haemonchus contortus* L1–L3 larvae. Chemical (GNPS and UPLC-DAD) and cultivation (MATRIX) profiling prompted a detailed chemical investigation of *Streptomyces* sp. S4S-00196A10, which yielded new anthelmintic polyketide goondapyrones A–J (**1**–**10**), together with the known actinopyrones A (**11**) and C (**12**). Structures for **1**–**12** were assigned on the basis of detailed spectroscopic and chemical analysis, with preliminary structure activity relationship analysis revealing selected γ-pyrones >50-fold and >13-fold more potent than isomeric α-pyrones against *D. immitis* mf motility (e.g., EC_50_ 0.05 μM for **1**; EC_50_ 2.7 μM for **5**) and *H. contortus* L1–L3 larvae development (e.g., EC_50_ 0.58 μM for **1**; EC_50_ 8.2 μM for **5**), respectively.

## 1. Introduction

As part of the ongoing investigations at the University of Queensland—Institute of Molecular Bioscience into the bioactive natural products of soil-derived microbes, we recently launched a first of its kind Australian citizen science initiative, Soils for Science (S4S) [1]. S4S engages with and enables the public to collect and share soil samples from urban backyards, rural properties, and native ecosystems across the nation as a valuable resource from which pure microbial (bacterial and fungal) isolates are recovered. Natural products of these isolates hold the prospect of new scientific knowledge that can inspire the development of new and improved pharmaceuticals and agrochemicals.

**Figure 1:**
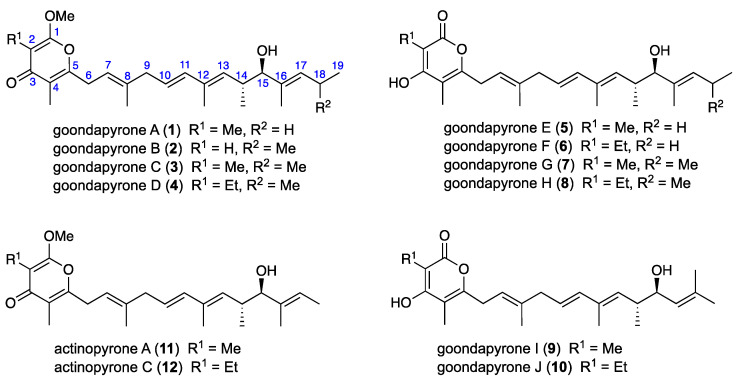
*Streptomyces* sp. S4S-00196A10 metabolites **1**–**12**.

To illustrate this potential, this report describes a proof-of-concept search for microbial natural products capable of inspiring new treatments for animals infected by life-threatening nematodes, including the heartworm *Dirofilaria immitis* that afflicts companion animals (e.g., dogs and cats,) and gastrointestinal parasite *Haemonchus contortus* that infects ruminant livestock (e.g., sheep). In this study, an assessment of ×19 pasture soils collected from Goondicum Station, a cattle property situated in an extinct volcanic crater near the headwaters of the Burnett River, Queensland, Australia, returned a library of microbial isolates which were dereplicated and prioritized based on chemical profiling (UPLC-DAD and UPLC-QTOF-GNPS) and anthelmintic potential against either *D. immitis* and/or *H. contortus.* This report provides an account of a detailed chemical analysis of just one of these isolates, *Streptomyces* sp. S4S-00196A10, that yielded the new anthelmintic polyketide goondapyrones A–J (**1**–**10**), along with the known actinopyrones A (**11**) and C (**12**) (Figure 1).

## 2. Results

M1 and ISP2 agar mother plates prepared from each of ×19 Goondicum Station soil samples were incubated for 14 d at 27 °C, after which manual colony picking returned ×704 pure microbial (bacterial and fungal) isolates. Each isolate was cultured on a fresh agar plate (ISP2 or M1, based on the mother plate media) to confirm purity and provide cells for cryopreservation, with residue mycelia/agar biomass per plate used to generate analytical scale EtOAc extracts. These extracts were dried in vacuo, resuspended in DMSO and dereplicated by chemical analysis (GNPS and UPLC-DAD) to ×559 unique extracts/isolates. Subsequent anthelmintic screening prioritized ×54 (10%) of these for their ability to inhibit the motility of *D. immitis* microfilaria (mf) and/or the development of *H. contortus* L1–L3 larvae (>75% inhibition at 25 μg/mL).

With a global natural product social (GNPS) [2] molecular network analysis of the prioritized extracts revealing a wealth of chemical diversity (Figure S3), attention was focused on those extracts/isolates that were the sole source/producer of a GNPS molecular family (i.e., cluster of MS/MS-related natural products), reasoning that targeting both anthelmintic activity and molecular rarity would enhance discovery prospects. Following this strategy, attention was drawn to *Streptomyces* sp. S4S-00196A10 as the sole producer of a GNPS molecular family that in a UPLC-DAD analysis aligned with major and minor metabolites exhibiting different UV–vis (DAD) chromophores (Figure S4). In an effort to determine how rare this class of chemistry may be, the GNPS molecular family was matched against a larger in-house library of ×1957 microbial extracts, derived from single agar plate cultivations of additional Goondicum (56%) and S4S (25%) soils, Australian sheep pasture plants (8%) and venomous snakes (1.8%), scorpions (1.5%), cone snails (2.5%), spiders (0.2%), centipedes (2%) and wasps (3%). This search revealed only a single replicate of S4S-00196A10, the Goondicum soil-derived *Streptomyces* sp. S4S-00246A11, an EtOAc extract of which also displayed activity against *D. immitis* mf.

Prior to scaled up cultivation, with a view to accessing both major and minor metabolites, we employed miniaturized 24-well plate cultivation profiling (MATRIX) [3,4] using ×11 different media compositions (Table S1) under solid phase as well as static and shaken conditions, supported by quantitative UPLC-DAD analysis (Figure S4). This process revealed solid phase glucose yeast extract agar (GYA) as optimal for the major co-metabolites, and a shaken broth comprised of glucose, malt and yeast extract, with peptone and soluble starch agar (D400) as optimal for the minor metabolites (as detected in ISP2).

An EtOAc extract prepared from a ×100 plate GYA 14 d, 30 °C cultivation was subjected to sequential solvent trituration and reversed phase fractionation to yield the new (γ-pyrone) goondapyrones A–D (**1**–**4**) and known (γ-pyrone) actinopyrones A (**11**) and C (**12**). By contrast, comparable fractionation of an EtOAc extract prepared from a D400 shaken broth cultivation (2 × 500 mL) yielded (α-pyrone) goondapyrones E–J (**5**–**10**). Structures were assigned to **1**–**12** by detailed spectroscopic analysis as summarized below, with the difference in UV–vis (DAD) spectra for major versus minor metabolites attributed to γ-pyrone versus α-pyrone chromophores, respectively.

HRESIMS measurements established molecular formula for **11** (C_25_H_36_O_4_, *m/z* M+Na ∆mmu +0.9) and **12** (C_26_H_38_O_4_, *m/z* M+Na ∆mmu −0.9), with the latter consistent with a higher homologue (+CH_2_) of the former. A literature comparison confirmed excellent concordance between the ^1^H NMR (CDCl_3_) data for **11** with those reported in 2006 [4] for actinopyrone A (Table S12, Figure S75). Likewise, the [α]_D_ for **11** (+13.6 in CH_2_Cl_2_) compared well with that reported for both natural and synthetic 14*R*,15*R* actinopyrone A [4], although it is important to note that the [α]_D_ in MeOH for **11** was of opposite sign (−14.6), highlighting the importance of making [α]_D_ comparisons (experimental vs literature) in the same solvent. Comparison of the 1D NMR (DMSO-*d*_6_) data for **12** (Table S14, Figures S78 and S79) with those for **11** (Table S13, Figures S76 and S77) allowed the key difference to be attributed to replacement of the 2-Me in **11** (δ_H_ 1.68, s; δ_C_ 6.8) with a 2-Et in **12** (δ_H_ 2.23, q, *J* 7.4 Hz, CH_2_; 0.91, t, *J* 7.4 Hz, CH_3_; δ_C_ 14.8, CH_2_; 12.9, CH_3_), thereby identifying **12** as actinopyrone C, first reported in 1986 as a co-metabolite with actinopyrone A from the Japanese soil-derived *Streptomyces pactum* S12538 [5,6]. Co-metabolites **1**–**10** co-clustered in the GNPS with **11**–**12** suggested structural (biosynthetic) similarities, as evident in the structure assignments outlined below.

**Figure 2:**
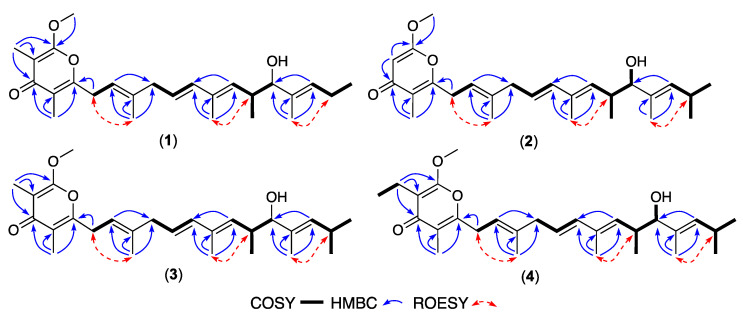
Selected 2D NMR (DMSO-*d*_6_) correlations for **1**–**4**.

HRESIMS measurements established isomeric molecular formula for **1** (C_26_H_38_O_4_, *m/z* M+Na ∆mmu +1.1) and **2** (C_26_H_38_O_4_, *m/z* M+Na ∆mmu +1.1), consistent with lower homologues (-CH_2_) of **11**. Comparison of the NMR (DMSO-*d*_6_) data for **1** (Table 1, Table 2 and Table S2, Figures S5–S10) with those for **11** revealed a high degree of concordance with the principle differences attributed to elongation of the polyketide chain (δ_H_ 0.89, t, *J* 7.5 Hz, H_3_-19; δ_C_ 14.1, C-19). Likewise, comparison of the NMR (DMSO-*d*_6_) data for **2** (Table 1, Table 2 and Table S3, Figures S12–S17) with those for **1** allowed the principle differences to be attributed to the absence of the 2-Me and inclusion of an 18-Me in **2** (δ_H_ 5.52, s, H-2; 0.87, d, *J* 6.1 Hz, H_3_-18; 0.89, d, *J* 5.9 Hz, H_3_-19; δ_C_ 87.7, C-2; 22.8, 18-CH_3_). Diagnostic 2D NMR (DMSO-*d*_6_) correlations supported the proposed planar structures for **1**–**2** (Figure 2), with a large *J*_10,11_ together with ROESY correlations between H_2_-6 and 8-CH_3_, H-14 and 12-CH_3_, and H_2_-18 and 16-CH_3_, allowing assignment of an all-*E* configuration. These observations, together with **1**–**2** and **11**–**12** sharing comparable [α]_D_ (MeOH) and ECD spectra (Figure S84), along with biosynthetic considerations, allowed the structures for goondapyrones A (**1**) and B (**2**) to be assigned as shown.

**Figure 3:**
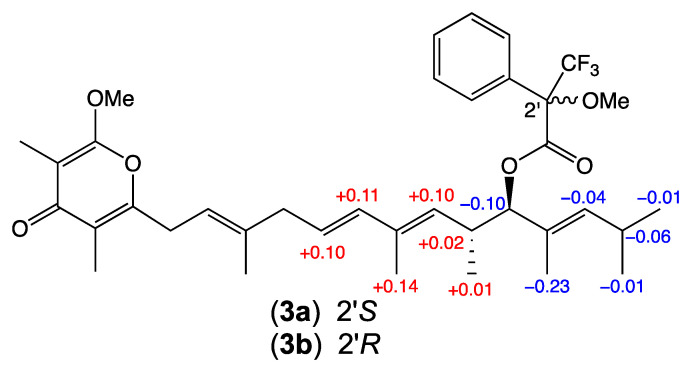
Mosher analysis of **3**. Δ*δ* (*δ_S_* − *δ_R_*) data for the *S*-MTPA (**3a**) and *R*-MTPA (**3b**).

HRESIMS measurements established molecular formula for **3** (C_27_H_40_O_4_, *m/z* M+Na ∆mmu +0.5) and **4** (C_28_H_42_O_4_, *m/z* M+Na ∆mmu −0.4), with **3** consistent with a higher homologue (+CH_2_) of **2**, and **4** consistent with a higher homologue (+CH_2_) of **3**. Comparison of the NMR (DMSO-*d*_6_) data for **3** (Table 1, Table 2 and Table S4, Figures S19–S24) with those for **2** revealed a high degree of concordance with the principle differences attributed to inclusion of a 2-Me in **3** (δ_H_ 1.68, s, 2-CH_3_; δ_C_ 6.8, 2-CH_3_). Likewise, comparison of the NMR (DMSO-*d*_6_) data for **4** (Table 1, Table 2 and Table S5, Figures S26–S31) with those for **3** allowed the principle differences to be attributed to inclusion of a 2-Et in **4** (δ_H_ 0.91, t, *J* 7.5 Hz, CH_3_; 2.23, q, *J* 7.5 Hz, CH_2_; δ_C_ 12.9, CH_3_; 14.8, CH_2_). A large value for *J*_10,11_ together with diagnostic 2D NMR (DMSO-*d*_6_) correlations supported the proposed planar structures for **3**–**4** inclusive of an all-*E* configuration (Figure 2). In addition to **1**–**4** sharing comparable [α]_D_ (MeOH) and ECD spectra (Figure S84), analysis of ^1^H NMR data for the Mosher esters **3a** (*S*-MTPA) and **3b** (*R*-MTPA) independently confirmed a 15*R* absolute configuration for **3** (Figure 3), consistent with data reported prior [4] for **11** and allowing the structures for goondapyrones C (**3**) and D (**4**) to be assigned as shown.

Whereas goondapyrones **1**–**4** and actinopyrones **11**–**12** produced under GYA solid phase cultivation share a common γ-pyrone chromophore (as detailed above), the metabolites **5**–**10** produced under D400 shaken broth cultivation co-clustered (GNPS molecular network) with **1**–**4** and **11**–**12** but featured a different chromophore. This difference was further evident in the 1D NMR (DMSO-*d*_6_) data where the 1-OMe moiety common to all of **1**–**4** and **11**–**12** was absent in **5**–**10**. Importantly, where the *O*-methylated γ-pyrone moieties in **1**–**4** and **11**–**12** cannot undergo tautomerisation, as demonstrated below, the pyrones in **5**–**10** can tautomerize and are expected to exist as the more thermodynamically stable α-pyrone tautomer, with a corresponding change in UV–vis (DAD) spectra.

**Figure 4:**
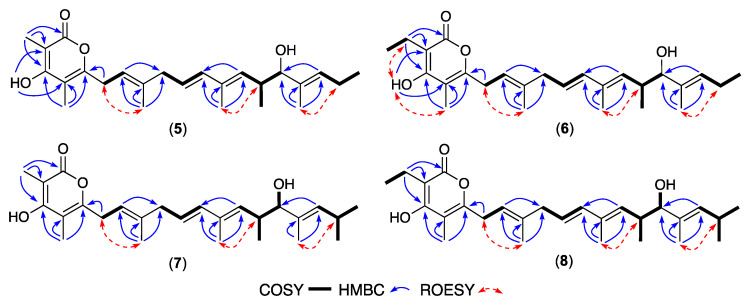
Selected 2D NMR (DMSO-*d*_6_) correlations for **5**–**8**.

HRESIMS measurements established molecular formula for **5** (C_25_H_36_O_4_, *m/z* M+Na ∆mmu −1.7) and **6** (C_26_H_38_O_4_, *m/z* M+Na ∆mmu +1.5), with **5** consistent with a lower homologue (-CH_2_) of **1** and **6** consistent with a higher homologue (+CH_2_) of **5**. Comparison of the NMR (DMSO-*d*_6_) data for **5** (Table 2, Table 3 and Table S6, Figures S33–S38) with **1** allowed the principle difference to be attributed to replacement of the 1-OMe in **1** with a chelated 3–OH in **5** (δ_H_ 10.48, s), with the alternate tautomeric regiochemistry evidenced by HMBC correlations from 3-OH to C-2 (δ_C_ 97.3) and C-4 (δ_C_ 106.4), and from 2-CH_3_ to C-1 (δ_C_ 164.4), C-2 and C-3 (δ_C_ 164.7) (Figure 4). Likewise, a comparison of the NMR (DMSO-*d*_6_) data for **6** (Table 2, Table 3 and Table S7, Figures S40–S45) with **5** attributed differences to the replacement of the 2-Me in **5** with a 2-Et in **6** (δ_H_ 0.93, t, *J* 7.4 Hz, CH_3_; 2.35, q, *J* 7.4 Hz, CH_2_; δ_C_ 12.8, CH_3_; 16.5, CH_2_). A large *J*_10,11_ value and diagnostic 2D NMR ROESY correlations supported the proposed planar structures for **5**–**6**, inclusive of an all-*E* configuration (Figure 4). The above, together with comparable [α]_D_ (MeOH) and ECD spectra (Figure S84) and biosynthetic considerations, permitted assignment of structures to goondapyrones E (**5**) and F (**6**).

HRESIMS measurements established molecular formula for **7** (C_26_H_38_O_4_, *m/z* M+Na ∆mmu +1.4) and **8** (C_27_H_40_O_4_, *m/z* M+Na ∆mmu +1.2), consistent with higher homologues (+CH_2_) of **5** and **6**, respectively. Comparison of the NMR (DMSO-*d*_6_) data for **7** and **8** (Table 2, Table 3, Tables S8 and S9, Figure 4, Figures S47–S52 and S54–S59) with **5** and **6** attributed the principle differences to the inclusion of an 18-Me moiety in both **7** and **8** (absent in both **5** and **6**) while retaining a common all-*E* configuration. These observations together with comparable [α]_D_ (MeOH), ECD spectra (Figure S84) and biosynthetic considerations allowed the structures for goondapyrones G (**7**) and H (**8**) to be assigned as shown.

**Figure 5:**
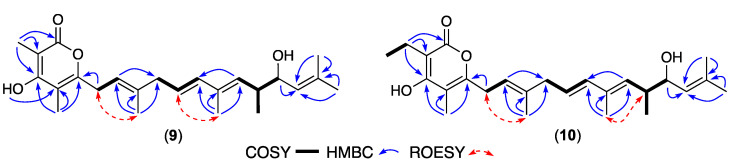
Selected 2D NMR (DMSO-*d*_6_) correlations for **9**–**10**.

HRESIMS measurements established molecular formula for **9** (C_24_H_34_O_4_, *m/z* M+Na ∆mmu +0.6) and **10** (C_25_H_36_O_4_, *m/z* M+Na ∆mmu +1.4), consistent with lower homologues (-CH_2_) of **5** and **6**, respectively. Comparison of the NMR (DMSO-*d*_6_) data for **9** and **10** (Table 4, Tables S10 and S11, Figure 5, Figures S61–S66 and S68–S73) with those for **5** and **6** revealed a common C-1 to C-15 scaffold inclusive of an a-pyrone moiety, alkyl branching and all-*E* configuration, with the principle differences attributed to the loss of the 16-Me and C-19 and incorporation of a 17-Me. These observations, together with comparable [α]_D_ (MeOH), ECD spectra (Figure S84) and biosynthetic considerations, allowed the structures for goondapyrones I (**9**) and J (**10**) to be assigned as shown.

## 3. Discussion

The polyketide pyrones **1**–**12** did not exhibit significant antibacterial activity against either the Gram-positive *Staphylococcus aureus* ATCC 25923 or Gram-negative *Escherichia coli* ATCC 11775 or antifungal activity against *Candida albicans* ATCC 10231 or cytotoxic activity against human colorectal (SW620) or lung (NCI-H460) carcinoma cells (EC_50_ > 30 μM) (Figures S80–S82). On the other hand, goondapyrones A–B (**1**–**2**) and actinopyrones A and C (**11** and **12**) were exceptionally potent inhibitors of the motility of *D. immitis* microfilaria (EC_50_ 0.01 to 0.07 μM), but exhibited marginal activity against the L4 larvae, and along with goondapyrone C (**3**) also significantly inhibited the development of *H. contortus* L1–L3 larvae (EC_50_ 0.15 to 0.66 μM) (Table 5). *D. immitis*, commonly known as heartworm, is a significant pathogen affecting animals such as dogs and cats [7]. It poses serious health risks, often leading to heart failure if untreated. Similarly, *H. contortus* is a major gastrointestinal parasite that infects ruminant livestock, including sheep and goats, leading to severe economic losses in the agricultural sector due to reduced productivity and increased mortality [8]. The discovery of natural products that exhibit potent anthelmintic activity against these parasites is crucial due to increasing resistance to existing anthelmintics such as ivermectins [9]. There is an urgent need to identify new bioactive compounds for managing and controlling these parasitic infections effectively.

**Figure 6:**
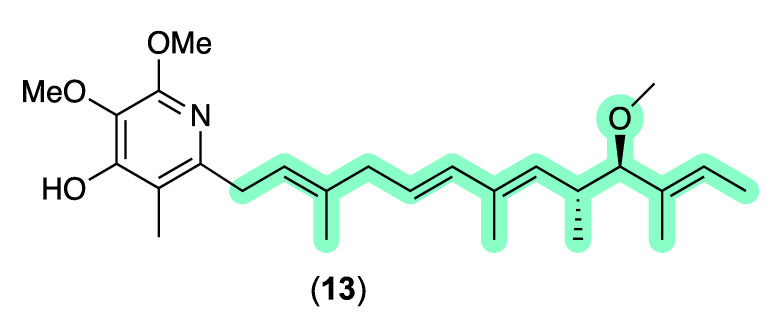
Piericidin B (**13**). Colour highlights—similarities with actinopyrone A (**11**) (green).

An anthelmintic structure activity relationship (SAR) assessment of **1**–**12** revealed that γ-pyrones **1**–**2** and **11**–**12** are up to 1000-fold more active than α-pyrones **5**–**10**, and up to 10-fold more effective against *D. immitis* mf than *H. contortus* L1–L3 larvae. As an aside, γ-pyrones **1**–**2** and **11**–**12** share common structural features with the well-known piericidin family of *Streptomyces* polyketides, with an authentic sample of piericidin B (**13**) active in our assays against *D. immitis* mf and *H. contortus* L1–L3 larvae (Figure 6, Table 5). Of note, piericidins can be detected across our microbe libraries with >4-fold higher frequency than the actinopyrones/goondapyrones, and typically also register as hits in our anthelmintic assays. Notwithstanding, as piericidins are known neurotoxins that inhibit NADH-ubiquinone oxidoreductase (Complex I) in the mitochondrial electron transport, anthelmintic hit extracts containing piericidins are typically deprioritized against further investigation. By contrast, pyrones **1**–**12** do not exhibit mammalian cell cytotoxicity (or antibiotic activity), with goondapyrone A (**1**) and actinopyrone A (**11**) being 35- and 170-fold more active against *D. immitis* mf, respectively, than authentic piericidin B (**13**).

## 4. Materials and Methods

For general experimental details, see Supplementary Materials.

### 4.1. Collection of Soils and Isolation of Microbes

Soil samples collected under the auspices of the Australian Soils for Science (S4S) citizen science initiative [1] from Goondicum Station situated in an extinct volcanic crater near the headwaters of the Burnett River, Queensland, Australia were used to inoculate ISP2 and M1 agar mother plates. After incubation at 27 °C for 14 days, manual colony picking yielded isolates were cultivated on fresh ISP2 or M1 agar plates (media choice based on that of the source mother plate) before being (i) photographed and the image uploaded to the S4S Gallery [1], (ii) cryopreserved at −80 °C, and (iii) an EtOAc extract was prepared, dried, resuspended in DMSO and archived at −20 °C.

### 4.2. Chemical Profiling (UPLC-DAD and UPLC-QTOF-GNPS)

EtOAc extracts prepared from agar plate cultivations of S4S soil-derived microbes were subjected to both UPLC-DAD and UPLC-QTOF-GNPS chemical profiling. UPLC-DAD chemical profiling involved injection of an aliquot of each analyte (2 μL at ~1 mg/mL in MeOH) through an Agilent 1290 infinity UPLC system (Zorbax SB-C_8_ RRHD 1.8 μm, 2.1 × 50 mm column, eluting at 0.417 mL/min, 2.50 min gradient elution from 90% H_2_O/MeCN to 100% MeCN with a constant 0.01% TFA modifier) equipped with a diode array multiple wavelength detector (DAD). UPLC-QTOF-GNPS chemical profiling involved injection of an aliquot of each analyte (1 μL at ~100 μg/mL in MeOH) through an Agilent 1290 Infinity II UPLC (Zorbax SB-C_8_ RRHD 1.8 μm, 2.1 × 50 mm column, eluting with 0.417 mL/min, 2.50 min gradient elution from 90% H_2_O/MeCN to 100% MeCN with a constant 0.1% formic acid/MeCN modifier) equipped with Agilent 6545 QTOF detector. UPLC-QTOF-(+) MS/MS data for GNPS analysis were acquired for all samples at a collision energy of 35 eV, transferred to the GNPS server [10], and resulting spectral network visualized using Cytoscape version 3.8.0 [11] (see Supplementary Materials for details).

### 4.3. Taxonomic Identification of S4S-00196A10 (and S4S-00246A11)

Genomic DNA was extracted from an ISP2 agar plate cultivation of target bacteria using the DNeasy Blood and Tissue Kit (Qiagen) as per the manufacturer’s protocol (see Supplementary Materials for details). BLAST analysis (NCBI database) showed that the amplified 16S rRNA sequence for S4S-00196A10 (accession number MG597592) has 98.9% identity with *Streptomyces parvisporogenes* strain MJM12043 (Figures S1a and S2). A comparable analysis of S4S-00246A11 revealed it to be a replicate of S4S-00196A10 (Figures S1b and S2).

### 4.4. Cultivation Profiling (MATRIX) of Streptomyces sp. S4S-00196A10

S4S-00196A10 was subjected to cultivation profiling in a 24-well plate (MATRIX) [3] system using 11 different media compositions (Table S1) under solid agar (1.5 g) as well as static (1.5 mL) and shaken (1.5 mL, 190 rpm) broth formats at 27 °C for 10 days. An additional set of control incubations were prepared from 11 different media solid-phase cultivations without inoculation. Individual MATRIX wells (Figure S4) were extracted in situ with EtOAc (2 mL) with the organic phases dried at 40 °C under a stream of N_2_, re-suspended in MeOH (100 mL). A portion of the analyte was then subjected to GNPS chemical profiling (as described above), while a second portion was treated with an internal calibrant (1-decyloxy-2,4-dinitrobenzene, 50 μg/mL) and subjected to UPLC-DAD-MS chemical profiling (as described above) (Figure S4).

### 4.5. Scale-Up Cultivation and Fractionation of Streptomyces sp. S4S-00196A10

*Cultivation in GY agar media:* A seed culture of S4S-00196A10 was prepared by inoculating D400 broth medium (70 mL) and shaking at 190 rpm at 30 °C for 5 days. Aliquots of the seed culture (100 μL) were used to inoculate glucose yeast (GY) agar plates (×100), and after incubation at 30 °C for 14 days, the combined agar was extracted with EtOAc (2 × 500 mL) and the organic phase concentrated in vacuo to yield an extract (585.4 mg). This extract was then subjected to sequential trituration to afford, after drying under nitrogen at 40 °C, *n*-hexane (120.5 mg), CH_2_Cl_2_ (298.6 mg) and MeOH (166.3 mg) solubles. The combined CH_2_Cl_2_ and MeOH solubles were fractionated by preparative reverse-phase HPLC (Zorbax RX-C_8_ 7 μm, 21.2 × 250 mm column, 20 mL/min gradient elution over 20 min from 90% H_2_O/MeCN to 100% MeCN, with a constant 0.01% TFA/MeCN modifier) to result in 40 fractions. Fractions 30–33 were combined and subjected to semi-preparative reverse-phase HPLC (Zorbax SB-C_18_ 5 μm, 9.4×250 mm column, 3 mL/min gradient elution over 20 min from 25% H_2_O/MeCN to 15% H_2_O/MeCN, with a constant 0.01% TFA/MeCN modifier) to produce pure goondapyrones A (**1**) (2.0 mg, 0.25%), B (**2**) (1.7 mg, 0.21%), C (**3**) (10.3 mg, 1.30%) and D (**4**) (1.5 mg, 0.19%), and actinopyrones A (**11**) (2.5 mg, 0.31%) and C (**12**) (4.0 mg, 0.50%).

*goondapyrone A (**1**)*. Light yellow oil; [α]^24^_D_ −15.0 (*c* 0.1, MeOH); ECD (MeOH) (Figure S84); 1D and 2D NMR (DMSO-*d*_6_) (Table 1, Table 2 and Table S2, Figures S5–S10); HRESIMS *m/z* 437.2679 [M+Na]^+^ (calculated for C_26_H_38_NaO_4_, 437.2662).

*goondapyrone B (**2**)*. Light yellow oil; [α]^24^_D_ −13.7 (*c* 0.1, MeOH); ECD (MeOH) (Figure S84); 1D and 2D NMR (DMSO-*d*_6_) (Table 1, Table 2 and Table S3, Figures S12–S17); HRESIMS *m/z* 437.2679 [M+Na]^+^ (calculated for C_26_H_38_NaO_4_, 437.2662).

*goondapyrone C (**3**)*. Light yellow oil; [α]^24^_D_ −21.2 (*c* 0.1, MeOH); ECD (MeOH) (Figure S84); 1D and 2D NMR (DMSO-*d*_6_) (Table 1, Table 2 and Table S4, Figures S19–S24); HRESIMS *m/z* 451.2824 [M+Na]^+^ (calculated for C_27_H_40_NaO_4_, 451.2819).

*goondapyrone D (**4**)*. Light yellow oil; [α]^24^_D_ −14.2 (*c* 0.1, MeOH); ECD (MeOH) (Figure S84); 1D and 2D NMR (DMSO-*d*_6_) (Table 1, Table 2 and Table S5, Figures S26–S31); HRESIMS *m/z* 465.2971 [M+Na]^+^ (calculated for C_28_H_42_NaO_4_, 465.2975).

*actinopyrone A (**11**)*. Light yellow oil; [α]^24^_D_ −13.0 (*c* 0.1, MeOH) and [α]^24^_D_ +13.6 (*c* 0.1, CH_2_Cl_2_); ECD (MeOH) (Figure S84); ^1^H NMR (CDCl_3_) (Table S12 and Figure S75) and ^1^H and ^13^C NMR (DMSO-*d*_6_) (Table S13 and Figures S76 and S77); HRESIMS *m/z* 423.2520 [M+Na]^+^ (calculated for C_25_H_36_NaO_4_, 423.2511).

*actinopyrone C (**12**)*. Light yellow oil; [α]^24^_D_ −14.6 (*c* 0.1, MeOH) and [α]^24^_D_ +13.6 (*c* 0.1, CH_2_Cl_2_); ECD (MeOH) (Figure S84); 1D NMR (DMSO-*d*_6_) (Table S14 and Figures S78 and S79); HRESIMS *m/z* 437.2657 [M+Na]^+^ (calculated for C_26_H_38_NaO_4_, 437.2668).

*Cultivation in D400 shaking broth media:* A seed culture of S4S-00196A10 was prepared by inoculating D400 broth medium (70 mL) and shaking at 190 rpm at 30 °C for 5 days. Aliquots of the seed culture (100 μL) were used to inoculate D400 broth media (2 × 500 mL), and after incubation at 190 rpm and 30°C for 14 days, the cultures were each extracted with EtOAc (2 × 500 mL) and the combined organic phase concentrated in vacuo to yield an extract (676.7 mg). This extract was then subjected to sequential trituration to afford after drying under nitrogen at 40 °C *n*-hexane (127.7 mg), CH_2_Cl_2_ (346.5 mg) and MeOH (202.5 mg) solubles. The combined CH_2_Cl_2_ and MeOH solubles were fractionated by reverse-phase HPLC (preparative: Zorbax RX-C_8_ 7 μm, 21.2 × 250 mm column, 20 mL/min gradient elution over 20 min from 90% H_2_O/MeCN to 100% MeCN, with a constant 0.01% TFA/MeCN modifier) to result in 40 fractions, which were further resolved by semi-preparative HPLC (Zorbax SB-C_18_ 5 μm, 250 × 9.4 mm column, 3 mL/min gradient elution over 20 min from 30% H_2_O/MeCN to 20% MeCN, with a constant 0.01% TFA/MeCN modifier) to yield goondapyrones E (**5**) (2.0 mg, 0.33%), F (**6**) (1.5 mg, 0.25%), G (**7**) (3.6 mg, 0.60%), H (**8**) (1.2 mg, 0.20%), I (**9**) (3.0 mg, 0.50%) and J (**10**) (1.1 mg, 0.18%).

*goondapyrone E (**5**)*. Light yellow oil; [α]^24^_D_ −13.1 (*c* 0.1, MeOH); ECD (MeOH) (Figure S84); 1D and 2D NMR (DMSO-*d*_6_) (Table 2, Table 3 and Table S6, Figures S33–S38); HRESIMS *m/z* 423.2523 [M+Na]^+^ (calculated for C_25_H_36_NaO_4_, 423.2506).

*goondapyrone F (**6**)*. Light yellow oil; [α]^24^_D_ −12.1 (*c* 0.1, MeOH); ECD (MeOH) (Figure S84); 1D and 2D NMR (DMSO-*d*_6_), (Table 2, Table 3 and Table S7, Figures S40–S45); HRESIMS *m/z* 437.2677 [M+Na]^+^ (calculated for C_26_H_38_NaO_4_, 437.2662).

*goondapyrone G (**7**)*. Light yellow oil; [α]^24^_D_ −15.5 (*c* 0.1, MeOH); ECD (MeOH) (Figure S84); 1D and 2D NMR (DMSO-*d*_6_), (Table 2, Table 3 and Table S8, Figures S47–S52); HRESIMS *m/z* 437.2679 [M+Na]^+^ (calculated for C_26_H_38_NaO_4_, 437.2662).

*goondapyrone H (**8**)*. Light yellow oil; [α]^24^_D_ −13.6 (*c* 0.1, MeOH); ECD (MeOH) (Figure S84); 1D and 2D NMR (DMSO-*d*_6_), (Table 2, Table 3 and Table S9, Figures S54–S59); HRESIMS *m/z* 451.2831 [M+Na]^+^ (calculated for C_27_H_40_NaO_4_, 451.2819).

*goondapyrone I (**9**)*. Light yellow oil; [α]^24^_D_ −13.0 (*c* 0.1, MeOH); ECD (MeOH) (Figure S84); 1D and 2D NMR (DMSO-*d*_6_), (Table 4 and Table S10, Figures S61–S66); HRESIMS *m/z* 409.2355 [M+Na]^+^ (calculated for C_24_H_34_NaO_4_, 409.2349).

*goondapyrone J (**10**)*. Light yellow oil; [α]^24^_D_ −8.4 (*c* 0.1, MeOH); ECD (MeOH) (Figure S84); 1D and 2D NMR (DMSO-*d*_6_), (Table 4 and Table S11, Figures S68–S73); HRESIMS *m/z* 423.2520 [M+Na]^+^ (calculated for C_25_H_36_NaO_4_, 423.2506).

### 4.6. Mosher Analysis of Goondapyrone C (***3***)

A solution of goondapyrone C (**3**) (1 mg) and *S*-α-methoxy-α-trifluoromethylphenylacetic acid [(*S*)-MTPA, 2.3 µL, 14.3 µmol, 6.2 eq.] in anhydrous CH_2_Cl_2_ (100 μL) was treated with dicyclocarbodiimide (DCC, 3.0 mg, 14.3 µmol, 6.2 eq.) and 4-dimethylaminopyridine (DMAP, 1.7 mg, 14.3 µmol, 6.2 eq.) at room temperature for 24 h, after which the reaction mixture was dried under N_2_ at 40 °C and the was residue extracted with EtOAc, which was in turn dried, dissolved in MeOH and purified by semi-preparative HPLC (Zorbax C_18_ 5 μm, 250 × 9.4 mm column, 3 mL/min gradient elution over 20 min from 10% H_2_O/MeCN to 100% MeCN, with a constant 0.01% TFA modifier) to yield the (*S*)-MTPA ester **3a** (0.76 mg, 51%). The procedure as outlined above was repeated using (*R*)-MTPA to yield the (*R*)-MTPA ester **3b** (0.71 mg, 48%).

*goondapyrone C (S)-MTPA ester* ***3a***. ^1^H NMR (DMSO-*d*_6_), (Figure S83); HRESIMS *m/z* 667.3245 [M+Na]^+^ (calculated for C_37_H_47_F_3_NaO_6_, 667.3217).

*goondapyrone C (R)-MTPA ester* ***3b***. ^1^H NMR (DMSO-*d*_6_), (Figure S83); HRESIMS *m/z* 667.3251 [M+Na]^+^ (calculated for C_37_H_47_F_3_NaO_6_, 667.3217).

### 4.7. Antiparasitic Assays

*D. immitis microfilariae motility inhibition assay*. *D. immitis* microfilariae were resuspended in RPMI 1640 media (Hyclone) and ~250 suspended parasites (in 100 µL) were delivered to microtiter plate wells containing test compounds **1**–**12** dissolved in 100% DMSO. Compounds were delivered to generate 10 doses (3.2-fold serial dilutions) covering the range of 25–0.0007 µg/mL. Microtiter plates were then incubated at 37 °C and 5% CO_2_ for ~72 h, after which they were imaged in a camera-based system and quantitative descriptors of parasite motility were calculated. Values were normalized using positive (1.0 µM gramicidin) and negative (DMSO) controls and motility inhibition calculated for each well. For each dose–response curve, an EC_50_ value was calculated using a four-parametric logistic model in Boehringer Ingelheim’s MegaLab application.

*H. contortus L1–L3 larvae development assay (LDA).* ~20 L1 stage *H. contortus* were delivered to microtiter plate wells containing compounds **1**–**12** dissolved in 100% DMSO and nutrient medium. Compounds were delivered to generate 10 doses (3.2-fold serial dilutions) in the range of 25–0.0007 µg/mL. Microtiter plates were then incubated at 27 °C and 85% relative humidity. After incubating for ~96 h to allow development from the L1 to the L3 stage, plates were imaged on a camera-based system and quantitative motility descriptors were calculated on resulting developed worms. Values were normalized using positive (1.0 µM ivermectin) and negative (DMSO) controls and motility inhibition calculated for each well. For each dose–response curve, an EC_50_ value was calculated using a four-parametric logistic model in Boehringer Ingelheim’s MegaLab application.

*Inhibition of motility of D. immitis L4 larvae.* Four to six L4-stage *D. immitis* worms suspended in a 1:1 mixture of NCTC-109 (Thermo Fisher, Waltham, MA, USA) and iMDM (with sodium bicarbonate and 25 mM HEPES (Sigma-Aldrich, Burlington, NJ, USA)) media supplemented with antibiotics/antimycotics and 1% Fetal Bovine Serum (Cytiva Hyclone, Logan, UT, USA) were added to wells of a microtiter plate. Compounds diluted in 100% DMSO were prediluted in culture media and added to the wells containing the L4s for a final assay volume of 100 μL. Compounds were delivered to generate 5 doses (4-fold serial dilution) covering the range of 25–0.098 µg/mL. Plates were held for ~72 h at 37 °C and 5% CO_2_ and imaged for calculation of motility descriptors as described above. Percentage motility inhibition was calculated by normalization of the motility descriptor values of a test compound well with the average motility of positive (5.0 μM monensin) and negative (DMSO) controls on each plate. EC_50_ values were calculated with Boehringer Ingelheim’s MegaLab application using a four-parametric logistic model.

## 5. Conclusions

This study demonstrates that there is still much to learn from the defensive natural products that have evolved and are encoded within the global microbiome while validating a citizen science-based approach to acquiring microbial diversity and an integrated platform of biological, chemical and cultivation profiling as an efficient strategy for exploring bioactive microbial natural products. In particular, this study demonstrates that a selection of polyketide pyrones isolated from a pasture soil-derived *Streptomyces* can exhibit promising anthelmintic activity against both the dog heartworm *D. immitis* mf and the livestock gastrointestinal parasite *H. contortus* L1–L3 larvae, without displaying toxicity towards bacterial, fungal or mammalian (human carcinoma) cells.

## Figures and Tables

**Table 1:** ^1^H NMR (DMSO-*d*_6_) data for goondapyrones A–D (**1**–**4**).

Position	(1) *δ*_H_, Mult (*J* in Hz)	(2) *δ*_H_, Mult (*J* in Hz)	(3) *δ*_H_, Mult (*J* in Hz)	(4) *δ*_H_, Mult (*J* in Hz)
2	-	5.52, s	-	-
6	3.35 ^A^	3.30, d *(7.3)*	3.35, d *(7.1)*	3.34, d *(7.3)*
7	5.29, t *(7.2)*	5.22, t *(7.3)*	5.28, t *(7.0)*	5.28, t *(7.3)*
9	2.77, d *(6.9)*	2.76, d *(7.5)*	2.77, d *(6.9)*	2.76, d *(7.0)*
10	5.46, dt *(15.5, 6.9)*	5.44, dt *(15.6, 7.5)*	5.46, dt *(15.5, 6.9)*	5.46, dt *(15.5, 7.0)*
11	6.02, d *(15.5)*	6.02, d *(15.6)*	6.01, d *(15.5)*	6.01, d *(15.5)*
13	5.27, d *(9.4)*	5.26, d *(9.4)*	5.25, d *(9.3)*	5.25, d *(9.5)*
14	2.53, m	2.53, m	2.54, m	2.53, m
15	3.59, d *(6.9)*	3.56, dd *(7.2, 4.1)*	3.56, d *(6.9)*	3.56, dd *(6.8, 4.0)*
16	-	-	-	-
17	5.25, t *(7.3)*	5.07, d *(9.1)*	5.07, d *(9.1)*	5.07, d *(9.1)*
18	1.96, m	2.47, m	2.46, m	2.46, m
19	0.89, t *(7.5)*	0.89, d *(5.9)*	0.88, d *(6.2)*	0.88, d *(6.3)*
2-CH_3_	1.68, s	-	1.68, s	-
2-CH_2_CH_3_	-	-	-	2.23, q *(7.5)*
2-CH_2_CH_3_	-	-	-	0.91, t *(7.5)*
4-CH_3_	1.83, s	1.80, s	1.82, s	1.82, s
8-CH_3_	1.70, s	1.66, s	1.69, s	1.69, s
12-CH_3_	1.65, s	1.64, s	1.64, s	1.64, s
14-CH_3_	0.77, d *(6.8)*	0.78, d *(6.6)*	0.77, d *(6.9)*	0.77, d *(6.7)*
16-CH_3_	1.50, s	1.51, s	1.51, s	1.51, s
18-CH_3_	-	0.87, d *(6.1)*	0.87, d *(6.2)*	0.87, d *(6.3)*
1-OCH_3_	3.91, s	3.83, s	3.91, s	3.90, s
15-OH	ND	4.49, d (*4.1*)	ND	4.50, d *(4.0)*

^A^—obscured by solvent, ND—not detected.

**Table 2:** ^13^C NMR (DMSO-*d*_6_) data for goondapyrones A–H (**1**–**8**).

Position	(1) *δ*_C_, Type ^A^	(2) *δ*_C_, Type ^A^	(3) *δ*_C_, Type ^A^	(4) *δ*_C_, Type ^A^	(5) *δ*_C_, Type ^A^	(6) *δ*_C_, Type ^A^	(7) *δ*_C_, Type ^A^	(8) *δ*_C_, Type ^A^
1	161.8, C	167.0, C	161.8, C	161.8, C	164.4, C	164.0, C	164.6, C	164.0, C
2	97.5, C	87.7, CH	97.5, C	103.7, C	97.3, C	103.5, C	97.1, C	103.5, C
3	179.4, C	180.0, C	179.4, C	178.8, C	164.7, C	164.2, C	165.2, C	164.2, C
4	116.8, C	117.4, C	116.8, C	117.2, C	106.4, C	106.4, C	106.6, C	106.4, C
5	157.2, C	158.9, C	157.2, C	157.3, C	156.7, C	156.9, C	156.6, C	156.9, C
6	29.4, CH_2_	29.5, CH_2_	29.4, CH_2_	29.4, CH_2_	29.7, CH_2_	29.7, CH_2_	29.7, CH_2_	29.7, CH_2_
7	117.8, CH	117.9, CH	117.8, CH	117.8, CH	118.6, CH	118.6, CH	118.7, CH	118.6, CH
8	137.7, C	137.7, C	137.7, C	137.7, C	136.9, C	136.9, C	136.9, C	136.9, C
9	42.1, CH_2_	42.1, CH_2_	42.1, CH_2_	42.1, CH_2_	42.2, CH_2_	42.2, CH_2_	42.3, CH_2_	42.2, CH_2_
10	124.0, CH	123.9, CH	123.9, CH	124.0, CH	124.0, CH	124.0, CH	124.0, CH	124.0, CH
11	136.6, CH	136.6, CH	136.6, CH	136.6, CH	136.6, CH	136.6, CH	136.6, CH	136.6, CH
12	132.0, C	132.0, C	132.0, C	132.0, C	132.1, C	132.1, C	132.1, C	132.0, C
13	135.4, CH	135.3, CH	135.3, CH	135.3, CH	135.4, CH	135.4, CH	135.4, CH	135.3, CH
14	36.0, CH	36.1, CH	36.1, CH	36.1, CH	36.1, CH	36.1, CH	36.2, CH	36.1, CH
15	80.4, CH	80.2, CH	80.2, CH	80.2, CH	80.4, CH	80.4, CH	80.3, CH	80.2, CH
16	136.2, C	134.4, C	134.4, C	134.4, C	136.2, C	136.2, C	134.4, C	134.4, C
17	127.1, CH	133.0, CH	133.0, CH	133.0, CH	127.1, CH	127.1, CH	133.1, CH	133.0, CH
18	20.2, CH_2_	26.2, CH	26.1, CH	26.2, CH	20.2, CH_2_	20.2, CH	26.2, CH	26.1, CH
19	14.1, CH_3_	23.0, CH_3_	23.0, CH_3_	23.0, CH_3_	14.1, CH_3_	14.1, CH_3_	23.0, CH_3_	23.0, CH_3_
2-CH_3_	6.8, CH_3_	-	6.8, CH_3_	-	9.2, CH_3_	-	9.3, CH_3_	-
2-CH_2_CH_3_	-	-	-	14.8, CH_2_	-	16.5, CH_2_	-	16.5, CH_2_
2-CH_2_CH_3_	-	-	-	12.9, CH_3_	-	12.8, CH_3_	-	12.8, CH_3_
4-CH_3_	9.5, CH_3_	9.1, CH_3_	9.5, CH_3_	9.5, CH_3_	9.8, CH_3_	9.8, CH_3_	9.9, CH_3_	9.8, CH_3_
8-CH_3_	16.3, CH_3_	16.3, CH_3_	16.3, CH_3_	16.3, CH_3_	16.3, CH_3_	16.3, CH_3_	16.4, CH_3_	16.3, CH_3_
12-CH_3_	12.7, CH_3_	12.7, CH_3_	12.7, CH_3_	12.7, CH_3_	12.8, CH_3_	12.8, CH_3_	12.8, CH_3_	12.8, CH_3_
14-CH_3_	17.9, CH_3_	17.9, CH_3_	17.9, CH_3_	17.9, CH_3_	17.9, CH_3_	17.9, CH_3_	17.9, CH_3_	17.9, CH_3_
16-CH_3_	11.4, CH_3_	11.6, CH_3_	11.6, CH_3_	11.6, CH_3_	11.4, CH_3_	11.4, CH_3_	11.6, CH_3_	11.6, CH_3_
18-CH_3_	-	22.8, CH_3_	22.8, CH_3_	22.8, CH_3_	-	-	22.9, CH_3_	22.8, CH_3_
1-OCH_3_	55.6, CH_3_	56.4, CH_3_	55.6, CH_3_	55.7, CH_3_	-	-	-	-

^A^—Resonance type assigned on the basis of 1D and 2D NMR analysis.

**Table 3:** ^1^H NMR (DMSO-*d*_6_) data for goondapyrones E–H (**5**–**8**).

Position	(5) *δ*_H_, Mult (*J* in Hz)	(6) *δ*_H_, Mult (*J* in Hz)	(7) *δ*_H_, Mult (*J* in Hz)	(8) *δ*_H_, Mult (*J* in Hz)
6	3.21, d *(6.9)*	3.21, d *(6.9)*	3.20, d *(7.0)*	3.20, d *(7.0)*
7	5.19, t *(6.9)*	5.19, t *(6.8)*	5.19, t *(7.0)*	5.19, t *(7.0)*
9	2.75, d *(6.9)*	2.75, d *(6.9)*	2.75, d *(6.9)*	2.75, d *(6.9)*
10	5.44, dt *(15.5, 6.9)*	5.44, dt *(15.5, 7.0)*	5.44, dt *(15.6, 6.9)*	5.44, dt *(15.6, 6.9)*
11	6.02, d *(15.5)*	6.02, d *(15.5)*	6.03, d *(15.6)*	6.02, d *(15.6)*
13	5.28, d *(9.3)*	5.28, d *(9.3)*	5.28, d *(9.4)*	5.27, d *(9.3)*
14	2.54, m	2.53, m	2.54, m	2.53, m
15	3.58, d *(7.1)*	3.58, d *(7.2)*	3.57, br d *(7.1)*	3.56, dd *(7.1, 4.3)*
17	5.25, t *(7.1)*	5.25, t *(7.2)*	5.08, d *(9.1)*	5.07, t *(9.0)*
18	1.96, m	1.96, m	2.48, m	2.47, m
19	0.89, t *(7.3)*	0.89, t *(7.5)*	0.89, d *(5.3)*	0.89, d *(5.5)*
2-CH_3_	1.82, s	-	1.81, s	-
2-CH_2_CH_3_	-	2.35, q *(7.4)*	-	2.35, q *(7.4)*
2-CH_2_CH_3_	-	0.93, t *(7.4)*	-	0.93, t *(7.4)*
4-CH_3_	1.88, s	1.88, s	1.87, s	1.88, s
8-CH_3_	1.66, s	1.66, s	1.66, s	1.66, s
12-CH_3_	1.65, s	1.65, s	1.65, s	1.64, s
14-CH_3_	0.77, d *(6.8)*	0.77, d *(6.8)*	0.78, d *(6.8)*	0.77, d *(6.9)*
16-CH_3_	1.50, s	1.50, s	1.52, s	1.51, s
18-CH_3_	-	-	0.88, d *(5.3)*	0.88, d *(5.5)*
3-OH	10.48, s	10.44, s	ND	ND
15-OH	ND	ND	4.48, br s	4.48, d *(4.3)*

ND—not detected.

**Table 4:** ^1^H and ^13^C NMR (DMSO-*d*_6_) data for goondapyrones I–J (**9**–**10**).

Position	(9) *δ*_C_, Type ^A^	*δ*_H_, Mult (*J* in Hz)	(10) *δ*_C_, Type ^A^	*δ*_H_, Mult (*J* in Hz)
1	164.4, C	-	164.0, C	-
2	97.3, C	-	103.5, C	-
3	164.7, C	-	164.2, C	-
4	106.4, C	-	106.4, C	-
5	156.6, C	-	156.9, C	-
6	29.6, CH_2_	3.21, d *(7.0)*	29.7, CH_2_	3.21, d *(7.0)*
7	118.6, CH	5.19, t *(7.0)*	118.6, CH	5.19, t *(7.0)*
8	136.9, C	-	136.9, C	-
9	42.2, CH_2_	2.75, d *(6.9)*	42.2, CH_2_	2.75, d *(6.9)*
10	124.0, CH	5.45, dt *(15.6, 6.9)*	124.0, CH	5.45, dt *(15.6, 6.9)*
11	136.6, CH	6.02, d *(15.6)*	136.6, CH	6.02, d *(15.6)*
12	132.1, C	-	132.1, C	-
13	135.4, CH	5.29, d *(9.3)*	135.4, CH	5.29, d *(9.3)*
14	36.1, CH	2.53, m	36.1, CH	2.54, m
15	80.5, CH	3.59, br d *(6.6)*	80.5, CH	3.59, br s
16	119.4, CH	5.33, q *(6.6)*	119.4, CH	5.32, q *(6.6)*
17	137.7, C	-	137.8, C	-
18	11.3, CH_3_	1.50, s	11.3, CH_3_	1.50, s
2-CH_3_	9.2, CH_3_	1.82, s	-	-
2-CH_2_CH_3_	-	-	16.5, CH_2_	-
2-CH_2_CH_3_	-	-	12.8, CH_3_	-
4-CH_3_	9.8, CH_3_	1.88, s	9.8, CH_3_	1.88, s
8-CH_3_	16.3, CH_3_	1.66, s	16.3, CH_3_	1.66, s
12-CH_3_	12.7, CH_3_	1.65, s	12.7, CH_3_	1.65, s
14-CH_3_	17.9, CH_3_	0.76, d *(6.8)*	17.9, CH_3_	0.75, d *(6.7)*
17-CH_3_	12.8, CH_3_	1.54, d (6.7)	12.8, CH_3_	1.54, d *(6.6)*
3-OH	-	10.46, s	-	10.44, br s
15-OH	-	4.46, d *(3.6)*	-	4.47, d *(3.9)*

^A^—Resonance type assigned on the basis of 1D and 2D NMR analysis.

**Table 5:** Summary of anthelmintic properties of **1**–**13** (plus positive controls)—EC_50_ (μM).

Compounds	*D. immitis* mf Motility	*D. immitis* L4 Motility	*H. contortus* L1–L3 Larval Development
**1**	0.05	34	0.58
**2**	0.07	31	0.63
**3**	22.3	48	0.66
**4**	3.2	25	15.3
**5**	2.7	>60	8.2
**6**	16.7	>60	>60
**7**	3.2	>60	11.9
**8**	3.2	>60	31.9
**9**	6.5	>60	17.4
**10**	1.4	45	23.8
**11**	0.01	27	0.15
**12**	0.02	34	0.65
**13**	1.77	>60	0.72
gramicidin	0.10	-	-
monensin	-	0.4	-
ivermectin	-	-	0.0005

## Data Availability

NMR and mass spectrometric data are available on request.

## Supplementary Materials

The following supporting information can be downloaded at: https://www.mdpi.com/article/10.3390/antibiotics13100989/s1, Bacterial taxonomy and phylogenetic analysis for *Streptomyces* spp. S4S-00196A10, together with NMR, HRESIMS, ECD and biological assay data for **1**–**12**.
