# Supplementary material for: Goondapyrones A–J: Polyketide α and γ Pyrone Anthelmintics from an Australian Soil-Derived Streptomyces sp
Source: Antibiotics (Basel). 2024 Oct 18;13(10):989. doi: 10.3390/antibiotics13100989 (PMC11505385; doi:10.3390/antibiotics13100989)
Supplement: Supplementary file 1 [file antibiotics-13-00989-s001.zip › antibiotics-3236617-supplementary.pdf]

Supplementary Materials

Goondapyrones A–J: Polyketide α and γ Pyrone Anthelmintics from an Australian Soil-Derived *Streptomyces* sp.

Shengbin Jin,[1] David F. Bruhn,[2] Cynthia T. Childs,[2] Erica Burkman,[2] Yovany Moreno,[2] Angela A. Salim, [1] Zeinab G. Khalil[1] and Robert J. Capon[1*]

[1] Institute for Molecular Bioscience, The University of Queensland, St Lucia, QLD 4072, Australia
[2] Boehringer Ingelheim Animal Health, USA Inc. 1730 Olympic Drive, Athens, GA 30601, USA
* Correspondence: r.capon@uq.edu.au

List of Figures

Figure S1. BLAST search (closest match) of S4S-00196A10 and S4S-00246A11 16S rRNA sequence. 7
Figure S2. Phylogenetic tree of S4S-00196A10 and S4S-00246A11; inset: S4S-00196A10 grown on ISP2 agar. [1] Yang, Z., Goldman, N., Friday, A., *Mol. Biol. Evol.* **1994**, *11*, 316–324; [2] Edgar, R.C. *BMC Bioinform.* **2004**, *5*, 113–113; [3] Okonechnikov, K.; Golosova, O.; Fursov, M.; *Bioinformatics* **2012**, *28*, 1166–1167. 8
Figure S3. GNPS analysis of S4S-00196A10 with compounds related nodes. (A): GNPS molecular network for the library of ×704 isolates form ×19 Goondicum soil samples; (B): GNPS molecular network for an expanded Capon lab library of an additional ×1957 microbial extracts; S4S-00246A11 is a duplicate of S4S-00196A10, obtained from another soil sample collected on Goondicum Station. CMB-SNF32 is a roadside soil-derived fungus, that produces the known and closely related fungal metabolite verticipyrone. 10
Figure S4. Top: Selection of UPLC-DAD (210 nm) chromatograms of S4S-00196A10 grown on 6 representative media, under 3 culture conditions (i: agar; ii: static broth; iii: shaking broth; iv: media blank; *: internal standard), and UV-vis spectra insets. Colour highlights: α-pyrones (blue) and γ-pyrones (red). Bottom: image of MATRIX culture plates. 12
Figure S5. ^{1}H NMR (DMSO-d_6) spectrum of goondapyrone A (**1**). 14
Figure S6. ^{13}C NMR (DMSO-d_6) and UV-vis (inset) spectra of goondapyrone A (**1**). 14
Figure S7. HSQC NMR (DMSO-d_6) spectrum of goondapyrone A (**1**). 15
Figure S8. HMBC NMR (DMSO-d_6) spectrum of goondapyrone A (**1**). 15
Figure S9. COSY NMR (DMSO-d_6) spectrum of goondapyrone A (**1**). 16
Figure S10. ROESY NMR (DMSO-d_6) spectrum of goondapyrone A (**1**). 16
Figure S11. HRESIMS spectrum for goondapyrone A (**1**). 17
Figure S12. ^{1}H NMR (DMSO-d_6) spectrum of goondapyrone B (**2**). 19
Figure S13. ^{13}C NMR (DMSO-d_6) and UV-vis (inset) spectra of goondapyrone B (**2**). 19
Figure S14. HSQC NMR (DMSO-d_6) spectrum of goondapyrone B (**2**). 20
Figure S15. HMBC NMR (DMSO-d_6) spectrum of goondapyrone B (**2**). 20
Figure S16. COSY NMR (DMSO-d_6) spectrum of goondapyrone B (**2**). 21
Figure S17. ROESY NMR (DMSO-d_6) spectrum of goondapyrone B (**2**). 21
Figure S18. HRESIMS spectrum for goondapyrone B (**2**). 22
Figure S19. ^{1}H NMR (DMSO-d_6) spectrum of goondapyrone C (**3**). 24
Figure S20. ^{13}C NMR (DMSO-d_6) and UV-vis (inset) spectra of goondapyrone C (**3**). 24
Figure S21. HSQC NMR (DMSO-d_6) spectrum of goondapyrone C (**3**). 25
Figure S22. HMBC NMR (DMSO-d_6) spectrum of goondapyrone C (**3**). 25
Figure S23. COSY NMR (DMSO-d_6) spectrum of goondapyrone C (**3**). 26
Figure S24. ROESY NMR (DMSO-d_6) spectrum of goondapyrone C (**3**). 26
Figure S25. HRESIMS spectrum for goondapyrone C (**3**). 27
Figure S26. ^{1}H NMR (DMSO-d_6) spectrum of goondapyrone D (**4**). 29
Figure S27. ^{13}C NMR (DMSO-d_6) and UV-vis (inset) spectra of goondapyrone D (**4**). 29
Figure S28. HSQC NMR (DMSO-d_6) spectrum of goondapyrone D (**4**). 30
Figure S29. HMBC NMR (DMSO-d_6) spectrum of goondapyrone D (**4**). 30
Figure S30. COSY NMR (DMSO-d_6) spectrum of goondapyrone D (**4**). 31
Figure S31. ROESY NMR (DMSO-d_6) spectrum of goondapyrone D (**4**). 31
Figure S32. HRESIMS spectrum for goondapyrone D (**4**). 32

Figure S33. ^{1}H NMR (DMSO-d_6) spectrum of goondapyrone E (**5**). 34
Figure S34. ^{13}C NMR (DMSO-d_6) and UV-vis (inset) spectra of goondapyrone E (**5**). 34
Figure S35. HSQC NMR (DMSO-d_6) spectrum of goondapyrone E (**5**). 35
Figure S36. HMBC NMR (DMSO-d_6) spectrum of goondapyrone E (**5**). 35
Figure S37. COSY NMR (DMSO-d_6) spectrum of goondapyrone E (**5**).................................. 36
Figure S38. ROESY NMR (DMSO-d_6) spectrum of goondapyrone E (**5**). 36
Figure S39. HRESIMS spectrum for goondapyrone E (**5**). 37
Figure S40. ^{1}H NMR (DMSO-d_6) spectrum of goondapyrone F (**6**). 39
Figure S41. ^{13}C NMR (DMSO-d_6) and UV-vis (inset) spectra of goondapyrone F (**6**). 39
Figure S42. HSQC NMR (DMSO-d_6) spectrum of goondapyrone F (**6**).................................. 40
Figure S43. HMBC NMR (DMSO-d_6) spectrum of goondapyrone F (**6**). 40
Figure S44. COSY NMR (DMSO-d_6) spectrum of goondapyrone F (**6**).................................. 41
Figure S45. ROESY NMR (DMSO-d_6) spectrum of goondapyrone F (**6**). 41
Figure S46. HRESIMS spectrum for goondapyrone F (**6**)... 42
Figure S47. ^{1}H NMR (DMSO-d_6) spectrum of goondapyrone G (**7**)... 44
Figure S48. ^{13}C NMR (DMSO-d_6) and UV-vis (inset) spectra of goondapyrone G (**7**).............. 44
Figure S49. HSQC NMR (DMSO-d_6) spectrum of goondapyrone G (**7**). 45
Figure S50. HMBC NMR (DMSO-d_6) spectrum of goondapyrone G (**7**)................................. 45
Figure S51. COSY NMR (DMSO-d_6) spectrum of goondapyrone G (**7**). 46
Figure S52. ROESY NMR (DMSO-d_6) spectrum of goondapyrone G (**7**)................................ 46
Figure S53. HRESIMS spectrum for goondapyrone G (**7**). 47
Figure S54. ^{1}H NMR (DMSO-d_6) spectrum of goondapyrone H (**8**)... 49
Figure S55. ^{13}C NMR (DMSO-d_6) and UV-vis (inset) spectra of goondapyrone H (**8**).............. 49
Figure S56. HSQC NMR (DMSO-d_6) spectrum of goondapyrone H (**8**). 50
Figure S57. HMBC NMR (DMSO-d_6) spectrum of goondapyrone H (**8**)................................. 50
Figure S58. COSY NMR (DMSO-d_6) spectrum of goondapyrone H (**8**). 51
Figure S59. ROESY NMR (DMSO-d_6) spectrum of goondapyrone H (**8**)................................ 51
Figure S60. HRESIMS spectrum for goondapyrone H (**8**). 52
Figure S61. ^{1}H NMR (DMSO-d_6) spectrum of goondapyrone I (**9**). 54
Figure S62. ^{13}C NMR (DMSO-d_6) and UV-vis (inset) spectra of goondapyrone I (**9**). 54
Figure S63. HSQC NMR (DMSO-d_6) spectrum of goondapyrone I (**9**). 55
Figure S64. HMBC NMR (DMSO-d_6) spectrum of goondapyrone I (**9**). 55
Figure S65. COSY NMR (DMSO-d_6) spectrum of goondapyrone I (**9**)..................................... 56
Figure S66. ROESY NMR (DMSO-d_6) spectrum of goondapyrone I (**9**). 56
Figure S67. HRESIMS spectrum for goondapyrone I (**9**). 57
Figure S68. ^{1}H NMR (DMSO-d_6) spectrum of goondapyrone J (**10**).. 59
Figure S69. ^{13}C NMR (DMSO-d_6) and UV-vis (inset) spectra of goondapyrone J (**10**)............. 59
Figure S70. HSQC NMR (DMSO-d_6) spectrum of goondapyrone J (**10**). 60
Figure S71. HMBC NMR (DMSO-d_6) spectrum of goondapyrone J (**10**). 60
Figure S72. COSY NMR (DMSO-d_6) spectrum of goondapyrone J (**10**). 61
Figure S73. ROESY NMR (DMSO-d_6) spectrum of goondapyrone J (**10**)................................ 61
Figure S74. HRESIMS spectrum for goondapyrone J (**10**). 62
Figure S75. ^{1}H NMR ($CDCl_3$) spectrum of actinopyrone A (**11**)... 63
Figure S76. ^{1}H NMR (DMSO-d_6) spectrum of actinopyrone A (**11**). 65
Figure S77. ^{13}C NMR (DMSO-d_6) spectrum of actinopyrone A (**11**). 65
Figure S78. ^{1}H NMR (600 MHz, DMSO-d_6) spectrum of actinopyrone C (**12**)......................... 67

Figure S79. ^{13}C NMR (DMSO-d_6) spectrum of actinopyrone C (**12**). 67
Figure S80. Antibacterial assays for **1-12**. 69
Figure S81. Antifungal assay for **1-12**. 70
Figure S82. Cytotoxicity assays for **1-12**. 71
Figure S83. ^{1}H NMR (DMSO-d_6) spectrum of **3a** (*S*-MTPA) and **3b** (*R*-MTPA) esters. 72
Figure S84. ECD spectra of goondapyrones and actinopyrones. 72

List of Tables

Table S1. MATRIX media compositions 11
Table S2. 1D and 2D NMR (DMSO-d_6) data for goondapyrone A (**1**). 13
Table S3. 1D and 2D NMR (DMSO-d_6) data for goondapyrone B (**2**). 18
Table S4. 1D and 2D NMR (DMSO-d_6) data for goondapyrone C (**3**). 23
Table S5. 1D and 2D NMR (600 MHz, DMSO-d_6) data for goondapyrone D (**4**). 28
Table S6. 1D and 2D NMR (DMSO-d_6) data for goondapyrone E (**5**). 33
Table S7. 1D and 2D NMR (DMSO-d_6) data for goondapyrone F (**6**). 38
Table S8. 1D and 2D NMR (600 MHz, DMSO-d_6) data for goondapyrone G (**7**). 43
Table S9. 1D and 2D NMR (600 MHz, DMSO-d_6) data for goondapyrone H (**8**). 48
Table S10. 1D and 2D NMR (DMSO-d_6) data for goondapyrone I (**9**). 53
Table S11. 1D and 2D NMR (DMSO-d_6) data for goondapyrone J (**10**). 58
Table S12. ^{1}H NMR ($CDCl_3$) data for actinopyrone A (**11**). 63
Table S13. 1D NMR (DMSO-d_6) data for actinopyrone A (**11**) 64
Table S14. 1D NMR (DMSO-d_6) data for actinopyrone A (**11**) and C (**12**). 66

1. Experimental

1.1 General Experimental

Chiroptical measurements ([α]$_D$) were obtained on a JASCO P-1010 polarimeter in a 100 × 2 mm cell at specified temperatures. ECD spectra were acquired on a Jasco J-810 spectropolarimeter (163–900 nm) as 0.5 mg/mL solutions in MeOH. Nuclear magnetic resonance (NMR) spectra were acquired on a Bruker Avance 600 MHz spectrometer with a 5 mm PASEL ^{1}H/D-^{13}C Z-Gradient probe. In all cases spectra were acquired at 25°C in solvents as specified with referencing to residual solvent ^{1}H or ^{13}C NMR resonances (DMSO-d_6: δ_H 2.50 and δ_C 39.5). High-resolution ESIMS spectra were obtained on a Bruker micrOTOF mass spectrometer by direct injection in MeOH at 3 μL/min using sodium formate clusters as an internal calibrant. High performance liquid chromatography-diode array-mass spectrometry (HPLC-DAD-MS) data were acquired on an Agilent 1260 series separation module equipped with a diode array detector and an Agilent G6125B series LC/MSD mass detector (Agilent Poroshell 120 SB-C_8 2.7 μm, 3.0×150 mm column, gradient elution at 0.8 mL/min over 6.5 min from 90% H_2O/MeCN to 100% MeCN with a constant 0.05% formic acid/MeCN modifier). Semi-preparative HPLCs were performed using Agilent 1100 series HPLC instruments with corresponding detectors, fraction collectors and software inclusively. UPLC chromatograms were obtained on Agilent 1290 infinity UPLC system equipped with a diode array multiple wavelength detector (Zorbax C_8 RRHD 1.8 μm, 2.1× 50 mm column, gradient elution at 0.417 mL/min over 2.50 min from 90% H_2O/MeCN to 100% MeCN with a constant 0.01% TFA/MeCN modifier). UPLC-QTOF analysis was performed on UPLC-QTOF instrument comprising of an Agilent 1290 Infinity II UPLC (Zorbax SB-C_8 RRHD 1.8 μm, 2.1× 50 mm column, gradient elution at 0.417 mL/min over 2.50 min from 90% H_2O/MeCN to 100% MeCN with a constant 0.1% formic acid/MeCN modifier) coupled to an Agilent 6545 Q-TOF. MS/MS analysis was performed on the same instrument for ions detected in the full scan at an intensity above 1000 counts at 10 scans/s, with an isolation width of 4 ~*m*/*z* using a fixed collision energy and a maximum of 3 selected precursors per cycle. Chemicals were purchased from Merck unless otherwise specified. Analytical-grade solvents were used for solvent extractions. Chromatography solvents were of HPLC grade supplied by Merck and filtered/degassed through 0.45 μm polytetrafluoroethylene (PTFE) membrane prior to use. Deuterated solvents were purchased from Cambridge Isotopes. Microorganisms were manipulated under sterile conditions using a Laftech class II biological safety cabinet and incubated in either MMM Friocell incubators (Lomb Scientific) or an Innova 42R incubator shaker (John Morris).

1.2 Taxonomic identification of S4S-00196A10 and S4S-00246A11

Genomic DNA was extracted from an ISP2 agar plate cultivation of target bacteria using the DNeasy Blood & Tissue Kit (Qiagen) as per the manufacturer's protocol. The 16S rRNA genes were amplified by PCR using the universal primers 27F (5′-AGAGTTTGATCCTGGCTCAG-3′) and 1492R (5′- TACGGCTACCTTCTTACGACTT-3′) purchased from Sigma-Aldrich. The PCR mixture (50 μL) contained genomic DNA (2 μL, 20–40 ng), EmeraldAmp GT PCR Master Mix (2XPremix) (25 μL), primer (0.2 μM, each), and H_2O (up to 50 μL). PCR was performed using the following conditions: initial denaturation at 95°C for 2 min, 40 cycles in series of 95°C for 20 s (denaturation), 56°C for 20 s (annealing) and 72°C for 30 s (extension), followed by one cycle at 72 °C for 5 min. The PCR products were purified with PCR purification kit (Qiagen) and sequenced.

16S rRNA sequence of *Streptomyces* sp. S4S-00196A10

CCGGCTTCGGGTGTTACCGACTTTCGTGACGTGACGGGCGGTGTGTACAAGGCCCGG
GAACGTATTCACCGCAGCAATGCTGATCTGCGATTACTAGCGACTCCGACTTCATGGG
GTCGAGTTGCAGACCCCAATCCGAACTGAGACCGGCTTTTTGAGATTCGCTCCACCT
CGCGGTATCGCAGCTCATTGTACCGGCCATTGTAGCACGTGTGCAGCCCAAGACATAA
GGGGCATGATGACTTGACGTCGTCCCCACCTTCCTCCGAGTTGACCCCGGCAGTCTC
CTGTGAGTCCCCATCACCCCGAAGGGCATGCTGGCAACACAGAACAAGGGTTGCGCT
CGTTGCGGGACTTAACCCAACATCTCACGACACGAGCTGACGACAGCCATGCACCAC
CTGTACACCGACCACAAGGGGGCGACCATCTCTGGCCGTTTCCGGTGTATGTCAAGC
CTTGGTAAGGTTCTTCGCGTTGCGTCGAATTAAGCCACATGCTCCGCCGCTTGTGCGG
GCCCCCGTCAATTCCTTTGAGTTTTAGCCTTGCGGCCGTACTCCCCAGGCGGGGAACT
TAATGCGTTAGCTGCGGCACGGACAACGTGGAATGTCGCCCACACCTAGTTCCCAAC
GTTTACGGCGTGGACTACCAGGGTATCTAATCCTGTTCGCTCCCCACGCTTTCGCTCCT
CAGCGTCAGTATCGGCCCAGAGATCCGCCTTCGCCACCGGTGTTCCTCCTGATATCTG
CGCATTTCACCGCTACACCAGGAATTCCGATCTCCCCTACCGAACTCTAGCCTGCCCG
TATCGAATGCAGACCCGGGGTTAAGCCCCGGGCTTTCACATCCGACGTGACAAGCCG
CCTACGAGCTCTTTACGCCCAATAATTCCGGACAACGCTCGCGCCCTACGTATTACCG
CGGCTGCTGGCACGTAGTTAGCCGGCGCTTCTTCTGCAGGTACCGTCACTTGCGCTTC
TTCCCTGCTGAAAGAGGTTTACAACCCGAAGGCCGTCATCCCTCACGCGGCGTCGCT
GCATCAGGCTTTCGCCCATTGTGCAATATTCCACTGCTGCCTCCCGTAGGAATCTGGG
CCGTGTCTCAGTCCCAGGGTGCCGGTCGCCCTCCAGGCGGCTACCCGTCGTCGCCTT
GGTAGGCCTCACCCCACCA-AAGCTGAAAGGC (1186 bp; S4S-00196A10)

Description	Scientific Name	Max Score	Total Score	Query Cover	E value	Per. Ident	Acc. Len	Accession
Streptomyces parvisporogenes strain MJM12043 16S ribosomal RNA gene, partial sequence	Streptomyces p...	2119	2119	100%	0.0	98.91%	1413	GU350508.1
Streptomyces ramulosus DSC3-6 gene for 16S ribosomal RNA, partial sequence	Streptomyces ra...	2108	2108	100%	0.0	98.74%	1374	LC536753.1
Streptomyces bingchengensis strain HBUM174849 16S ribosomal RNA gene, partial sequence	Streptomyces bi...	2108	2108	100%	0.0	98.74%	1439	EU841632.1
Uncultured bacterium clone AKIW955 16S ribosomal RNA gene, partial sequence	uncultured bacte...	2104	2104	100%	0.0	98.66%	1487	DQ129579.1
Streptomyces buecherae strain NA00687 chromosome, complete genome	Streptomyces b...	2102	12615	100%	0.0	98.66%	9069632	CP054929.1
Streptomyces sp. strain KIB-HW85 16S ribosomal RNA gene, partial sequence	Streptomyces sp.	2102	2102	100%	0.0	98.66%	1359	MT534112.1
Streptomyces sp. strain RCPA2-9 16S ribosomal RNA gene, partial sequence	Streptomyces sp.	2102	2102	100%	0.0	98.66%	1521	MN058246.1
Streptomyces sp. 124 16S ribosomal RNA gene, partial sequence	Streptomyces s...	2102	2102	100%	0.0	98.66%	1332	KP748239.1
Streptomyces sp. strain Je 1-426 16S ribosomal RNA gene, partial sequence	Streptomyces sp.	2102	2102	100%	0.0	98.66%	1344	OP720566.1
Streptomyces sp. strain Je 1-424 16S ribosomal RNA gene, partial sequence	Streptomyces sp.	2102	2102	100%	0.0	98.66%	1372	OP720564.1
Streptomyces sp. strain Je 1-149 16S ribosomal RNA gene, partial sequence	Streptomyces sp.	2102	2102	100%	0.0	98.66%	1382	OP720414.1
Streptomyces sp. qua23-1 16S ribosomal RNA gene, partial sequence	Streptomyces s...	2102	2102	100%	0.0	98.66%	1375	KM035649.1
Candidatus Streptomyces philanthi from Philanthus bilunatus USA-BS-33 16S ribosomal RNA gene, partial s...	Candidatus Stre...	2102	2102	100%	0.0	98.66%	1376	KC607722.1
Streptomyces sp. strain ZMR2-80 16S ribosomal RNA gene, partial sequence	Streptomyces sp.	2102	2102	100%	0.0	98.66%	1382	MZ833418.1
Streptomyces youssoufiensis strain ICTA169 16S ribosomal RNA gene, partial sequence	Streptomyces y...	2102	2102	100%	0.0	98.66%	1385	KF006390.1
Candidatus Streptomyces philanthi 16S ribosomal RNA gene, partial sequence	Candidatus Stre...	2102	2102	100%	0.0	98.66%	1417	JN104609.1
Streptomyces buecherae strain AC541 chromosome, complete genome	Streptomyces b...	2102	12597	100%	0.0	98.66%	8416829	CP060404.1
Streptomyces youssoufiensis strain OUC6819 16S ribosomal RNA gene, partial sequence	Streptomyces y...	2102	2102	100%	0.0	98.66%	1516	KY582832.1
Candidatus Streptomyces philanthi biovar pulcher 16S ribosomal RNA gene, partial sequence	Candidatus Stre...	2102	2102	100%	0.0	98.66%	1313	DQ375799.1
Streptomyces luteoverticillatus strain HBUM173698 16S ribosomal RNA gene, partial sequence	Streptomyces lu...	2102	2102	100%	0.0	98.66%	1439	FJ486315.1
Streptomyces luteoverticillatus strain 173699 16S ribosomal RNA gene, partial sequence	Streptomyces lu...	2102	2102	100%	0.0	98.66%	1442	EU593751.1

16S rRNA sequence of *Streptomyces* sp. S4S-00246A11

TGCTACCACCGGCTTCGGGTGTTACCGACTTTCCTGACGTGACGGGCGGTGTGTACA
AGGCCCGGGAACGTATTCACCGCAGCAATGCTGATCTGCGATTACTAGCGACTCCGAC
TTCATGGGGTCGAGTTGCAGACCCCAATCCGAACTGAGACCGGCTTTTTGAGATTCG
CTCCACCTCGCGGTATCGCAGCTCATTGTACCGGCCATTGTAGCACGTGTGCAGCCCA
AGACATAAGGGGCATGATGACTTGACGTCGTCCCCACCTTCCTCCGAGTTGACCCCG
GCAGTCTCCTGTGAGTCCCCATCACCCCGAAGGGCATGCTGGCAACACAGAACAAG
GGTTGCGCTCGTTGCGGGACTTAACCCAACATCTCACGACACGAGCTGACGACAGCC
ATGCACCACCTGTACACCGACCACAAGGGGGCGACCATCTCTGGCCGTTTCCGGTGT
ATGTCAAGCCTTGGTAAGGTTCTTCGCGTTGCGTCGAATTAAGCCACATGCTCCGCCG
CTTGTGCGGGCCCCCGTCAATTCCTTTGAGTTTTAGCCTTGCGGCCGTACTCCCCAGG
CGGGGAACTTAATGCGTTAGCTGCGGCACGGACAACGTGGAATGTCGCCCACACCTA
GTTCCCAACGTTTACGGCGTGGACTACCAGGGTATCTAATCCTGTTCGCTCCCCACGC
TTTCGCTCCTCAGCGTCAGTATCGGCCCAGAGATCCGCCTTCGCCACCGGTGTTCCTC
CTGATATCTGCGCATTTCACCGCTACACCAGGAATTCCGATCTCCCCTACCGAACTCTA
GCCTGCCCGTATCGAATGCAGACCCGGGGTTAAGCCCCGGGCTTTCACATCCGACGT
GACAAGCCGCCTACGAGCTCTTTACGCCCAATAATTCCGGACAACGCTCGCGCCCTAC
GTATTACCGCGGCTGCTGGCACGTAGTTAGCCGGCGCTTCTTCTGCAGGTACCGTCAC
TTGCGCTTCTTCCCTGCTGAAAGAGGTTTACAACCCGAAGGCCGTCATCCCTCACGC
GGCGTCGCTGCATCAGGCTTTCGCCATTGTGCAATATTCCCACTGCTGCCTCCCGTAG
GAATCTGGGCCGTGTCTCATTCCCAGTGTGGCCGGTCGCCCTCTCAGCCGGCTACCGT
CCTCGCCTTGGTAG (1166 bp; S4S-00246A11)

	Description	Scientific Name	Max Score	Total Score	Query Cover	E value	Per. Ident	Acc. Len	Accession
☐	Streptomyces parvisporogenes strain MJM12043 16S ribosomal RNA gene, partial sequence	Streptomyces p...	2097	2097	99%	0.0	99.23%	1413	GU350508.1
☐	Streptomyces ramulosus DSC3-6 gene for 16S ribosomal RNA, partial sequence	Streptomyces ra...	2085	2085	99%	0.0	99.05%	1374	LC536753.1
☐	Streptomyces sp. strain KIB-HW85 16S ribosomal RNA gene, partial sequence	Streptomyces sp.	2080	2080	99%	0.0	98.97%	1359	MT534112.1
☐	Streptomyces sp. strain RCPA2-9 16S ribosomal RNA gene, partial sequence	Streptomyces sp.	2080	2080	99%	0.0	98.97%	1521	MN058246.1
☐	Streptomyces sp. strain HSW2009 16S ribosomal RNA gene, partial sequence	Streptomyces sp.	2080	2080	99%	0.0	98.97%	1516	OR826630.1
☐	Streptomyces bingchengensis strain HBUM174849 16S ribosomal RNA gene, partial sequence	Streptomyces bi...	2080	2080	99%	0.0	98.97%	1439	EU841632.1
☐	Uncultured bacterium clone AKIW955 16S ribosomal RNA gene, partial sequence	uncultured bacte...	2076	2076	99%	0.0	98.89%	1487	DQ129579.1
☐	Streptomyces buecherae strain NA00687 chromosome, complete genome	Streptomyces b...	2074	12449	99%	0.0	98.88%	9069632	CP054929.1
☐	Streptomyces sp. strain 1N409 16S ribosomal RNA gene, partial sequence	Streptomyces sp.	2074	2074	99%	0.0	98.88%	1493	PP758746.1
☐	Streptomyces sp. strain 9N102 16S ribosomal RNA gene, partial sequence	Streptomyces sp.	2074	2074	99%	0.0	98.88%	1492	PP758721.1
☐	Streptomyces sp. EUSKR2S82 gene for 16S ribosomal RNA, partial sequence	Streptomyces s...	2074	2074	99%	0.0	98.88%	1429	LC085600.1
☐	Streptomyces sp. 124 16S ribosomal RNA gene, partial sequence	Streptomyces s...	2074	2074	99%	0.0	98.88%	1332	KP748239.1
☐	Streptomyces sp. strain Je 1-149 16S ribosomal RNA gene, partial sequence	Streptomyces sp.	2074	2074	99%	0.0	98.88%	1382	OP720414.1
☐	Candidatus Streptomyces philanthi from Philanthus bilunatus USA-BS-33 16S ribosomal RNA gene, partial s...	Candidatus Stre...	2074	2074	99%	0.0	98.88%	1376	KC607722.1
☐	Streptomyces sp. strain ZMR2-80 16S ribosomal RNA gene, partial sequence	Streptomyces sp.	2074	2074	99%	0.0	98.88%	1382	MZ833418.1
☐	Streptomyces youssoufiensis strain ICTA169 16S ribosomal RNA gene, partial sequence	Streptomyces y...	2074	2074	99%	0.0	98.88%	1385	KF006390.1
☐	Candidatus Streptomyces philanthi 16S ribosomal RNA gene, partial sequence	Candidatus Stre...	2074	2074	99%	0.0	98.88%	1417	JN104609.1
☐	Streptomyces buecherae strain AC541 chromosome, complete genome	Streptomyces b...	2074	12430	99%	0.0	98.88%	8416829	CP060404.1
☐	Streptomyces youssoufiensis strain OUC6819 16S ribosomal RNA gene, partial sequence	Streptomyces y...	2074	2074	99%	0.0	98.88%	1516	KY582832.1

Figure S1. BLAST search (closest match) of S4S-00196A10 and S4S-00246A11 16S rRNA sequence.

1.3 Phylogenetic analysis of S4S-00196A10 and S4S-00246A11

A phylogenetic tree obtained by PhyML Maximum Likelihood analysis was constructed using the top similar 16S rRNA sequences displayed after BLAST on the Refseq RNA NCBI database using the target bacteria 16S rRNA as the query. The JC69 model was used to infer phylogenetic sequences.[1] Sequence alignments were produced with the MUSCLE program.[2] A phylogenetic tree was constructed using the UGENE program using the aforementioned models and visualized using Ugene's tree view.[3]

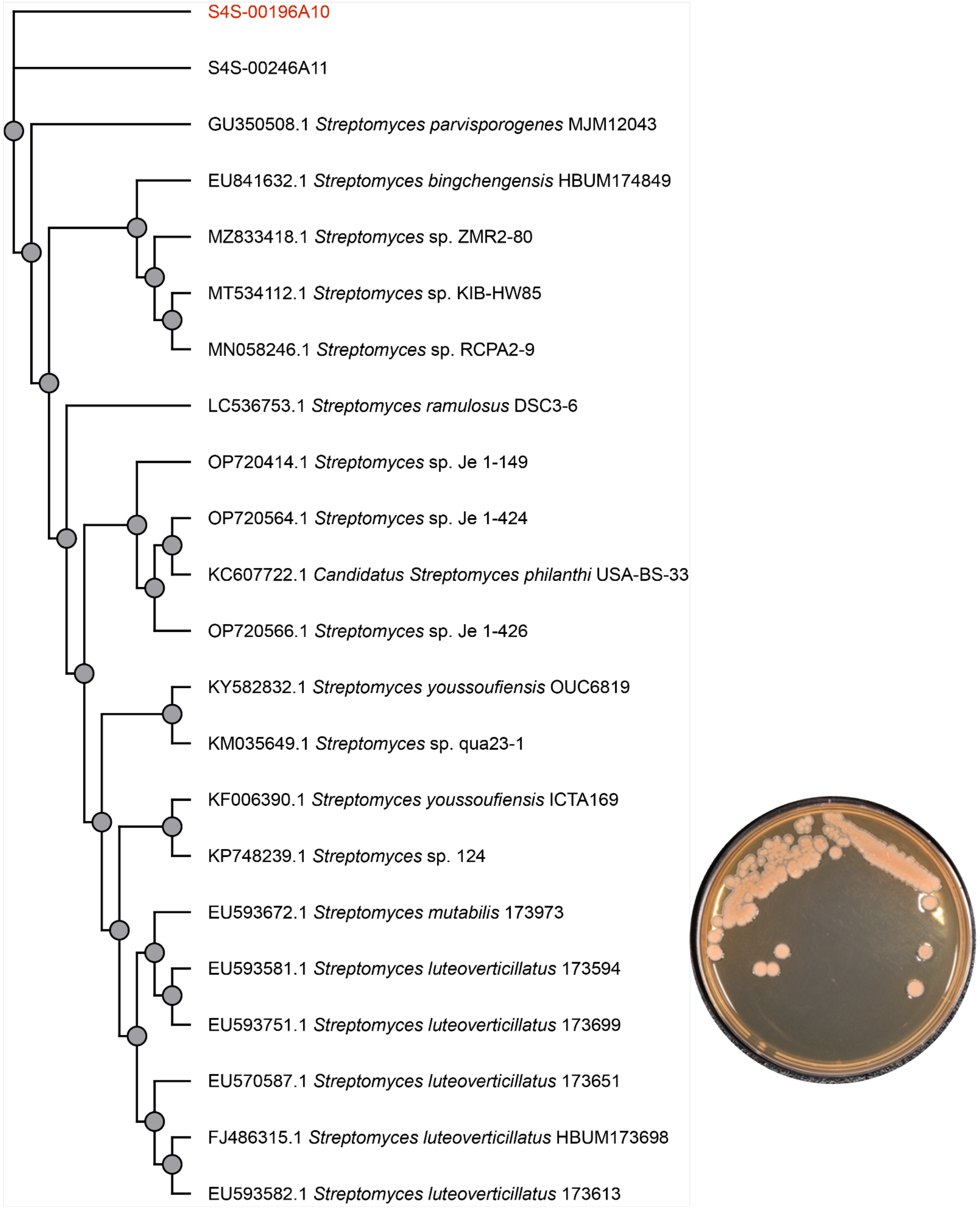

Figure S2. Phylogenetic tree of S4S-00196A10 and S4S-00246A11; inset: S4S-00196A10 grown on ISP2 agar. [1] Yang, Z., Goldman, N., Friday, A., *Mol. Biol. Evol.* **1994**, *11*, 316–324; [2] Edgar, R.C. *BMC Bioinform.* **2004**, *5*, 113–113; [3] Okonechnikov, K.; Golosova, O.; Fursov, M.; *Bioinformatics* **2012**, *28*, 1166–1167.

1.4 GNPS Molecular Networking

Aliquots (1 μL) of individual EtOAc extracts (100 μg/mL in MeOH) were analysed on UPLC-QTOF instrument comprising an Agilent 1290 Infinity II UPLC (Zorbax SB-C_8 RRHD 1.8 μm, 2.1×50 mm column, gradient elution at 0.417 mL/min over 2.50 min from 90% H_2O/MeCN to 100% MeCN with a constant 0.1% formic acid/MeCN modifier) coupled to an Agilent 6545 QTOF mass detector. UPLC-QTOF-(+) MS/MS data acquired for all samples at a collision energy of 35 eV were converted from Agilent MassHunter data files (.d) to an mzXML file format using MSConvert software and transferred to the GNPS server. Molecular networking was performed using the GNPS data analysis workflow employing the spectral clustering algorithm with a cosine score of 0.7 and a minimum of 6 matched peaks. The resulting spectral network was imported into Cytoscape version 3.8.0[8] and visualized using a ball–stick layout, where nodes represent parent mass, and cosine score was reflected by edge thickness. Also, group abundances were set as pie charts, which reflected the intensity of MS signals. MS/MS fragmentation analysis was performed on the same machine for ions detected in the full scan range at an intensity above 200 counts at 5 scans/sec, with an isolation width of ~4 *m/z* using fixed collision energy and a maximum of 3 selected precursors per cycle. General instrument parameters included gas temperature at 325°C, drying gas at 10 L/min, nebulizer at 20 psi, sheath gas temperature at 400°C, fragmentation voltage at 180 V and skimmer at 45 V.

(A)

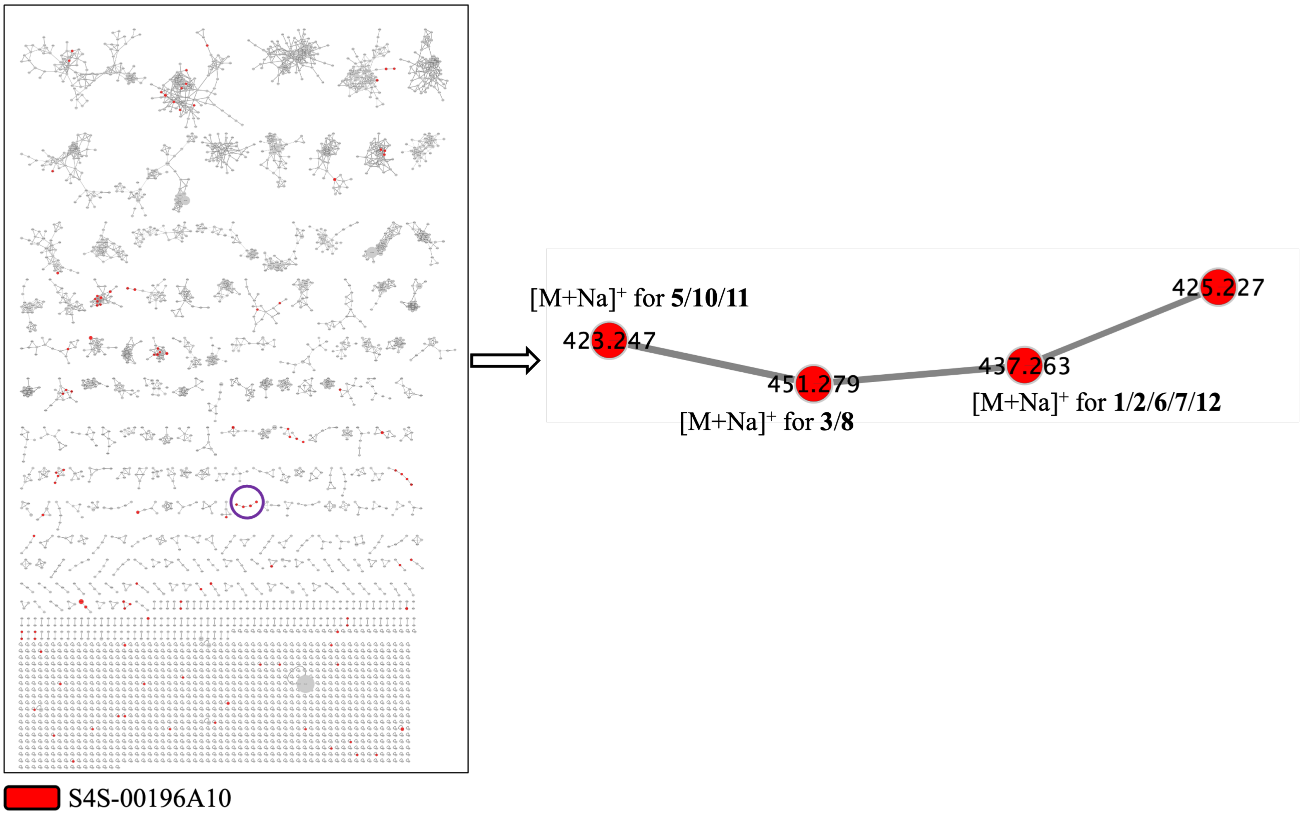

(B)

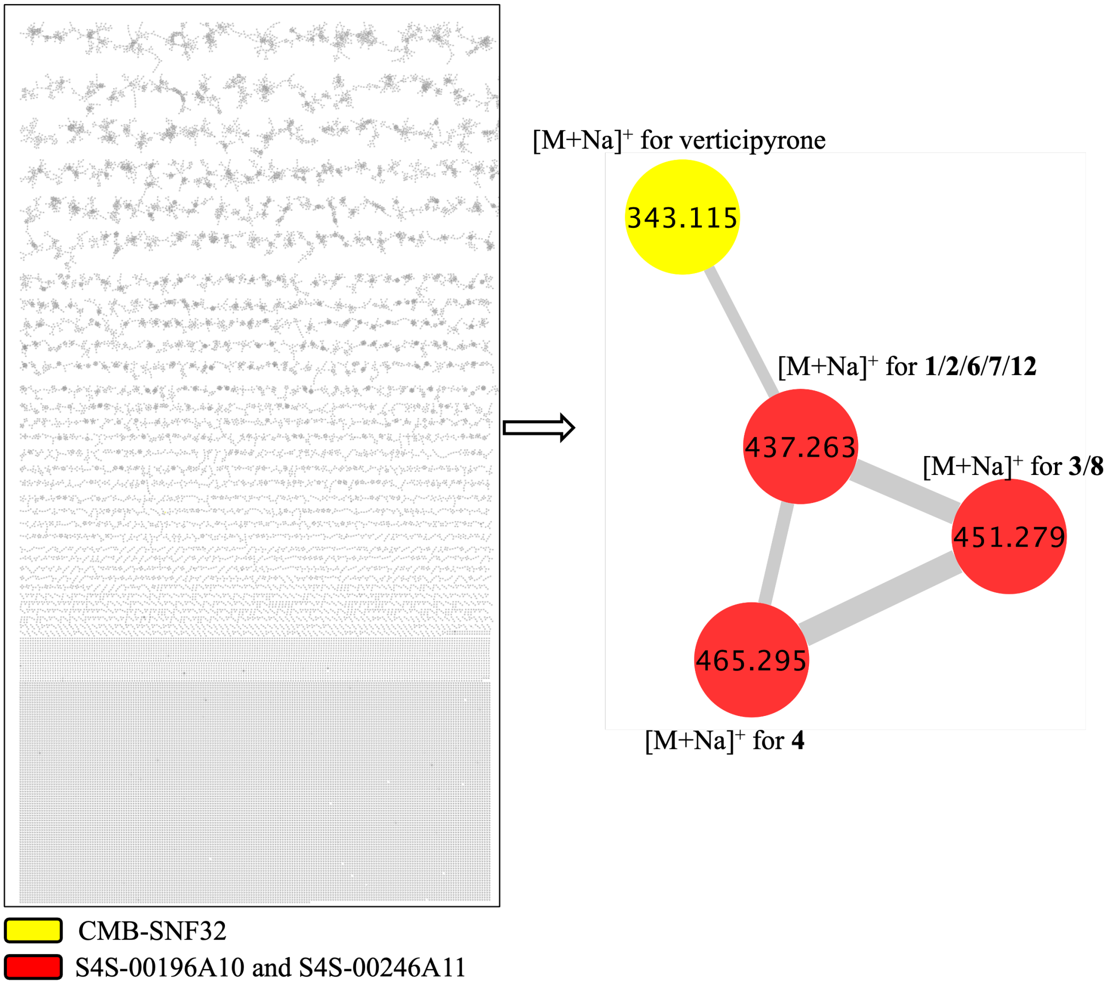

Figure S3. GNPS analysis of S4S-00196A10 with compounds related nodes. (A): GNPS molecular network for the library of ×704 isolates form ×19 Goondicum soil samples; (B): GNPS molecular network for an expanded Capon lab library of an additional ×1957 microbial extracts; S4S-00246A11 is a duplicate of S4S-00196A10, obtained from another soil sample collected on Goondicum Station. CMB-SNF32 is a roadside soil-derived fungus, that produces the known and closely related fungal metabolite verticipyrone.

Table S1. MATRIX media compositions

Medium	Composition (per Litre)
**333**	Glucose (5.0 g), Peptone (3.0 g), Soluble Starch (10.0 g), Yeast extract (3.0 g), $CaCO_3$ (2.0 g), Agar (20.0 g)
**Glycerol Casein Agar (CGA)**	Glycerol (Chem-Supply) (30.0 g), Casein peptone (Amyl) (2.0 g), K_2HPO_4(Chem-Supply) (1.0 g), NaCl (Chem-Supply) (1.0 g), $MgSO_4.7H_2O$ (AnalaR) (0.5 g), Trace element solution (5.0 mL)*, Agar (Amyl) (20.0 g)<br><br>***Trace element solution:**<br>$CaCl_2.2H_2O$ (3.0 g), $FeC_6O_7H_5$ (1.0 g), $MnSO_4$ (0.2 g), $ZnCl_2$ (0.1 g), $CuSO_4.5H_2O$ (0.025 g), $Na_2B_4O_7.10H_2O$ (0.02 g), $CoCl_2$ (0.004 g), $Na_2MoO_4.2H_2O$ (0.01 g), Distilled H_2O (1 L) (Filter sterilize)
**D400**	Glucose (5.0 g), Malt extract (3.0 g), Peptone (3.0 g), Soluble Starch (20.0 g), Yeast extract (5.0 g), $CaCO_3$ (3.0 g), Agar (20.0 g)
**Glucose Yeast Extract Starch (GYA)**	Yeast extract (Difco) (4.0 g), Malt extract (Difco) (10.0 g), Glucose (Country Brewers) (4.0 g), $CaCO_3$ (Univar Ajax) (2.0 g), Soluble starch (Difco) (20.0 g), Agar (Amyl) (20.0 g), Adjust pH to 7.3
**ISP2 + Mannitol Agar (IMA)**	Yeast extract (Difco) (4.0 g), Malt extract (Difco) (10.0 g), Glucose (Country Brewers) (4.0 g), Mannitol (Amyl) (40.0 g), Agar (Amyl) (20.0 g), Adjust pH to 7.3
**ISP-2**	Yeast extract (Difco) (4.0 g), Malt extract (Difco) (10.0 g), Glucose (Country Brewers) (4.0 g), Agar (Amyl) (18.0 g), Adjust pH to 7.3
**M1**	Peptone (2.0 g), Yeast extract (4.0 g), Starch (10.0 g), Agar (18.0 g), pH 7.0
**M2**	Mannitol (40.0 g), Maltose (40.0 g), Yeast extract (10.0 g), K_2HPO_4 (2.0 g), $MgSO_4.7H_2O$ (0.5 g), $FeSO_4.7H_2O$ (0.01 g), Agar (20 g)
**SDA**	Peptic digest of animal tissue (5.0 g), Pancreatic digest of casein (5.0 g), Dextrose (40.0 g), Agar (15 g), pH 5.6
**YES**	Sucrose (150 g), Yeast extract (20 g), $MgSO_4.7H_2O$ (0.5 g), $ZnSO_4.7H_2O$ (0.01 g), $CuSO_4.5H_2O$ (0.005 g), Agar (20.0 g)
**Modified YEME media**	Yeast extract (Difco) (3.0 g), Bacto peptone (Difco) (5.0 g), Oxoin malt extract (3.0 g), Glucose (10.0 g), Sucrose (170.0 g), Agar (15.0 g)

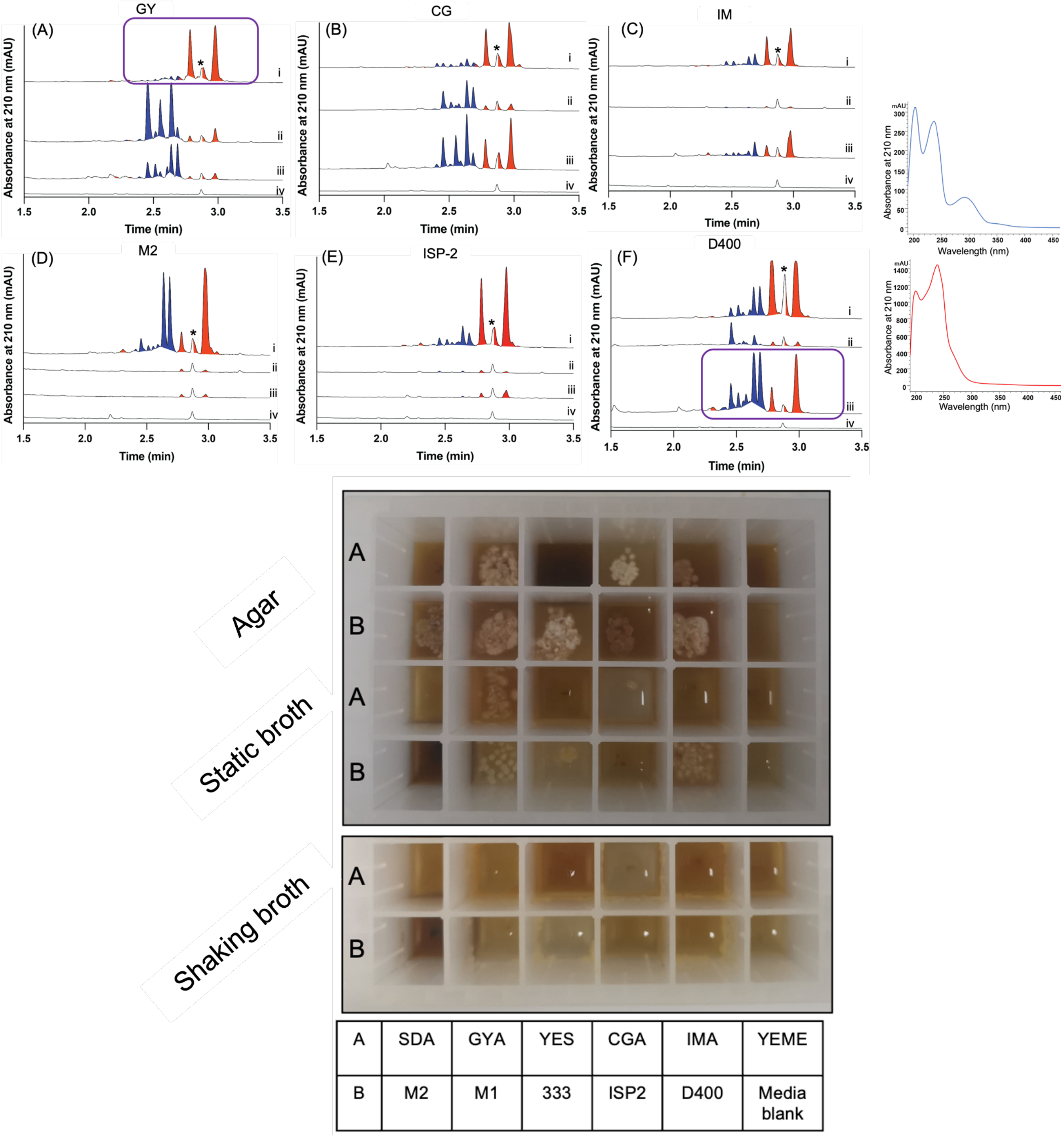

A	SDA	GYA	YES	CGA	IMA	YEME
B	M2	M1	333	ISP2	D400	Media blank

Figure S4. Top: Selection of UPLC-DAD (210 nm) chromatograms of S4S-00196A10 grown on 6 representative media, under 3 culture conditions (i: agar; ii: static broth; iii: shaking broth; iv: media blank; *: internal standard), and UV-vis spectra insets. Colour highlights: α-pyrones (blue) and γ-pyrones (red). Bottom: image of MATRIX culture plates.

2. Spectroscopic characterisations of compounds 1-12

2.1. Goondapyrone A (1)

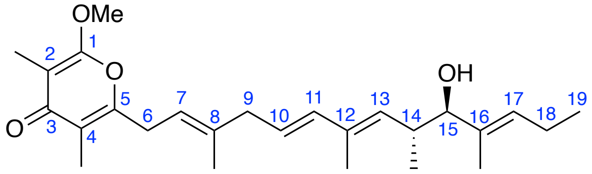

Table S2. 1D and 2D NMR (DMSO-d_6) data for goondapyrone A (**1**).

Pos.	δ_C	δ_H, mult (*J* in Hz)	COSY	^{1}H-^{13}C HMBC	ROESY
1	161.8				
2	97.5				
3	179.4				
4	116.8				
5	157.2				
6	29.4	3.35[A], d	7	5, 7, 8	7, 4-CH_3, 8-CH_3
7	117.8	5.29, t *(7.2)*	6	6, 9, 8-CH_3	6, 9
8	137.7				
9	42.1	2.77, d *(6.9)*	10	7, 8, 10, 11, 8-CH_3	7, 10, 11, 8-CH_3
10	124.0	5.46, dt *(15.5, 6.9)*	9, 11	9, 12	9, 12-CH_3
11	136.6	6.02, d *(15.5)*	9, 10	9, 12, 13, 12-CH_3	9, 10
12	132.0				
13	135.4	5.27, d *(9.4)*	14	11, 14, 12-CH_3	11, 14, 15, 14-CH_3
14	36.0	2.53, m	13, 15, 14-CH_3	12, 13, 15, 14-CH_3	15, 12-CH_3, 14-CH_3, 16-CH_3
15	80.4	3.59, d *(6.9)*	14	13, 16, 17, 14-CH_3, 16-CH_3	13, 14, 14-CH_3, 16-CH_3
16	136.2				
17	127.1	5.25, t *(7.3)*	18	15, 18, 19, 16-CH_3	15, 19
18	20.2	1.96, m	17, 19	16, 17, 19	17, 19, 16-CH_3
19	14.1	0.89, t *(7.5)*	18	17, 18	17, 18
15-OH					
2-CH_3	6.8	1.68, s		1, 2, 3	
4-CH_3	9.5	1.83, s	6	3, 4, 5	6
8-CH_3	16.3	1.70, s	6, 7	7, 8, 9	6, 9
12-CH_3	12.7	1.65, s	13	11, 12, 13	10, 14
14-CH_3	17.9	0.77, d *(6.8)*	14	13, 14, 15	13, 14, 15
16-CH_3	11.4	1.50, s	17	15, 16, 17	14, 15, 18
1-OCH_3	55.6	3.91, s		1	2-CH_3

[A] obscured by solvent

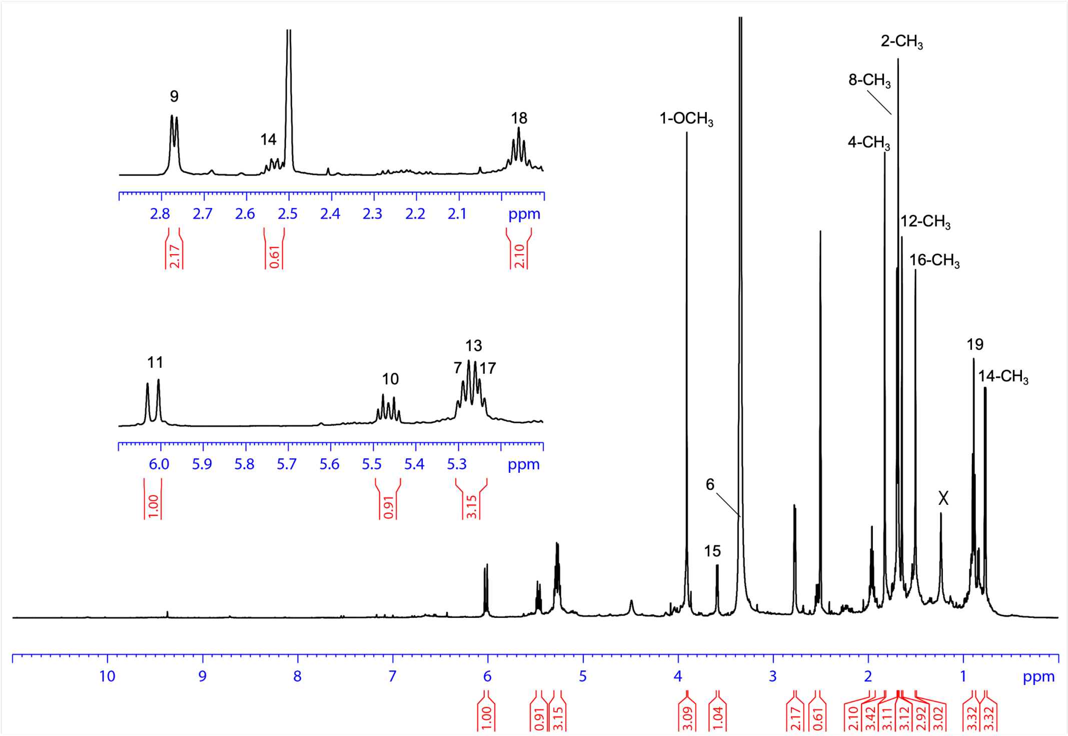

Figure S5. ^{1}H NMR (DMSO-d_6) spectrum of goondapyrone A (**1**).

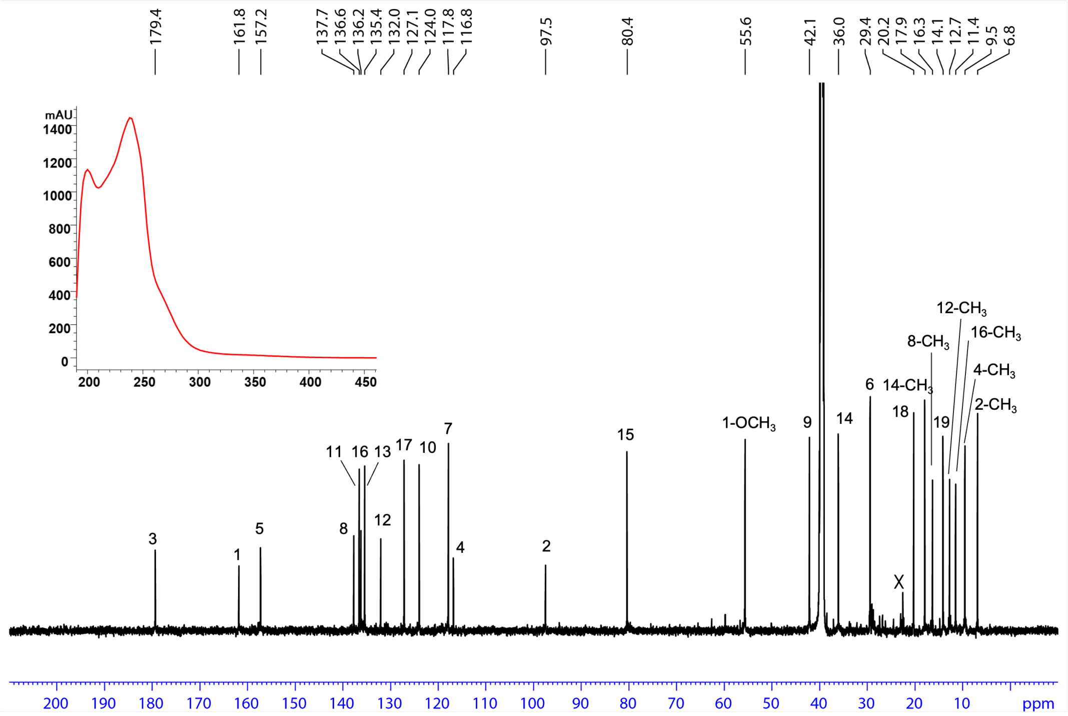

Figure S6. ^{13}C NMR (DMSO-d_6) and UV-vis (inset) spectra of goondapyrone A (**1**).

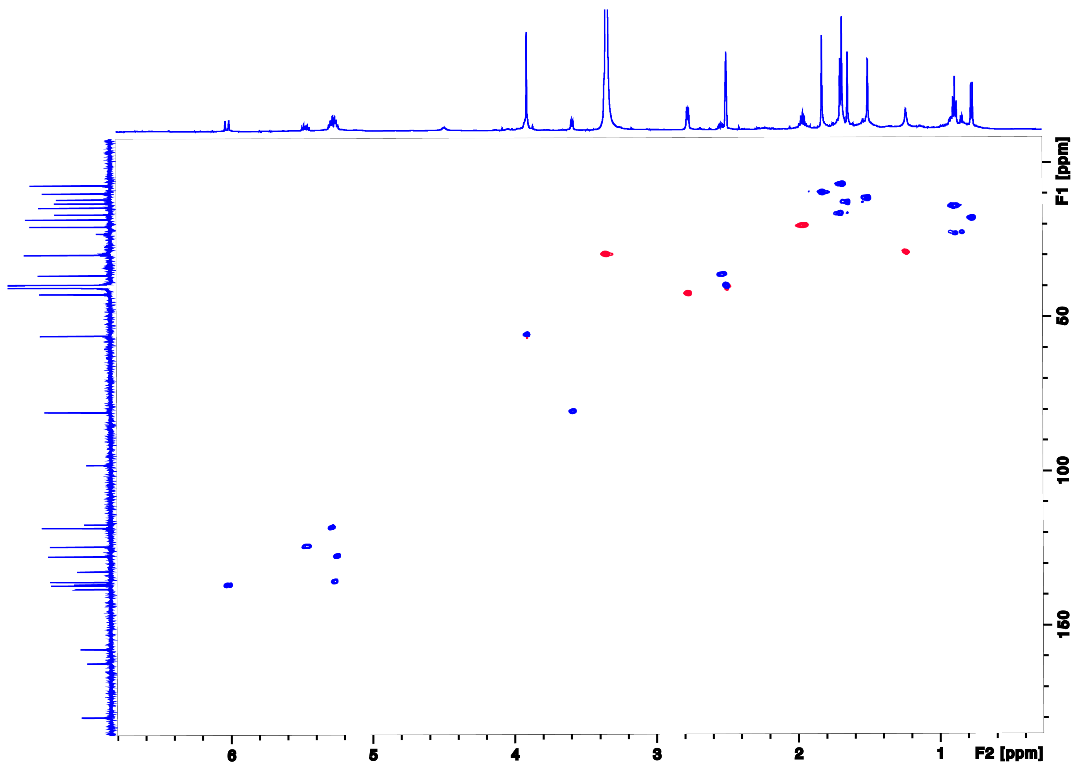

Figure S7. HSQC NMR (DMSO-d_6) spectrum of goondapyrone A (**1**).

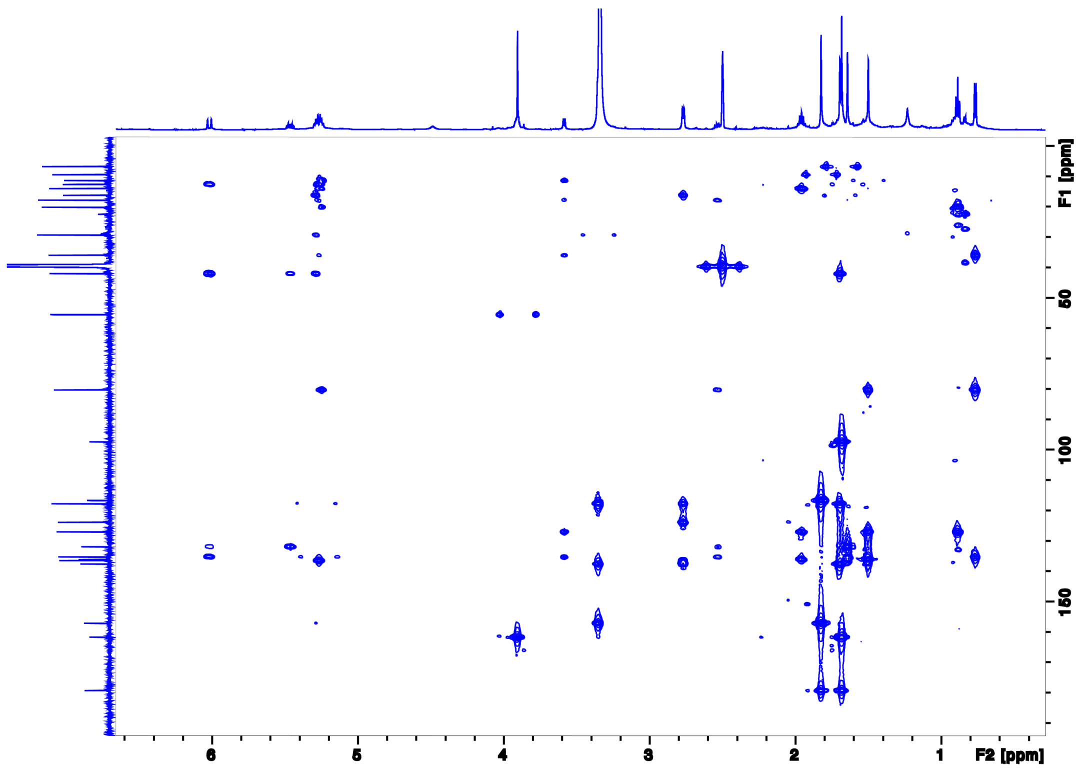

Figure S8. HMBC NMR (DMSO-d_6) spectrum of goondapyrone A (**1**).

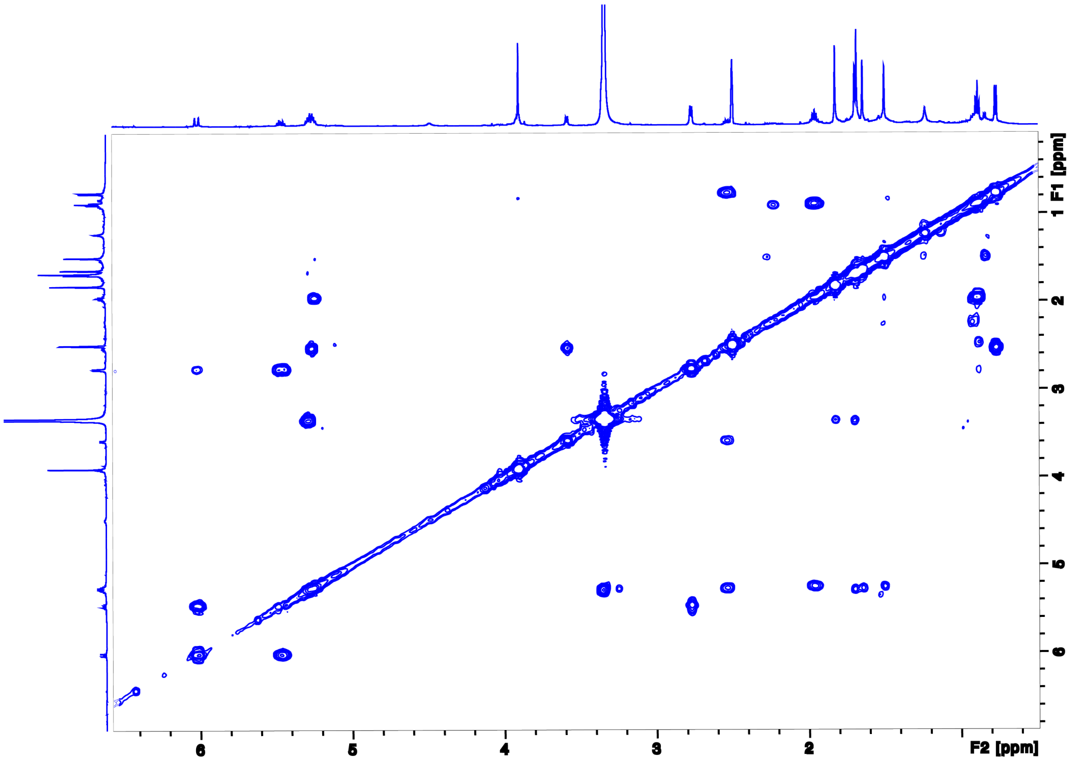

Figure S9. COSY NMR (DMSO-d_6) spectrum of goondapyrone A (**1**).

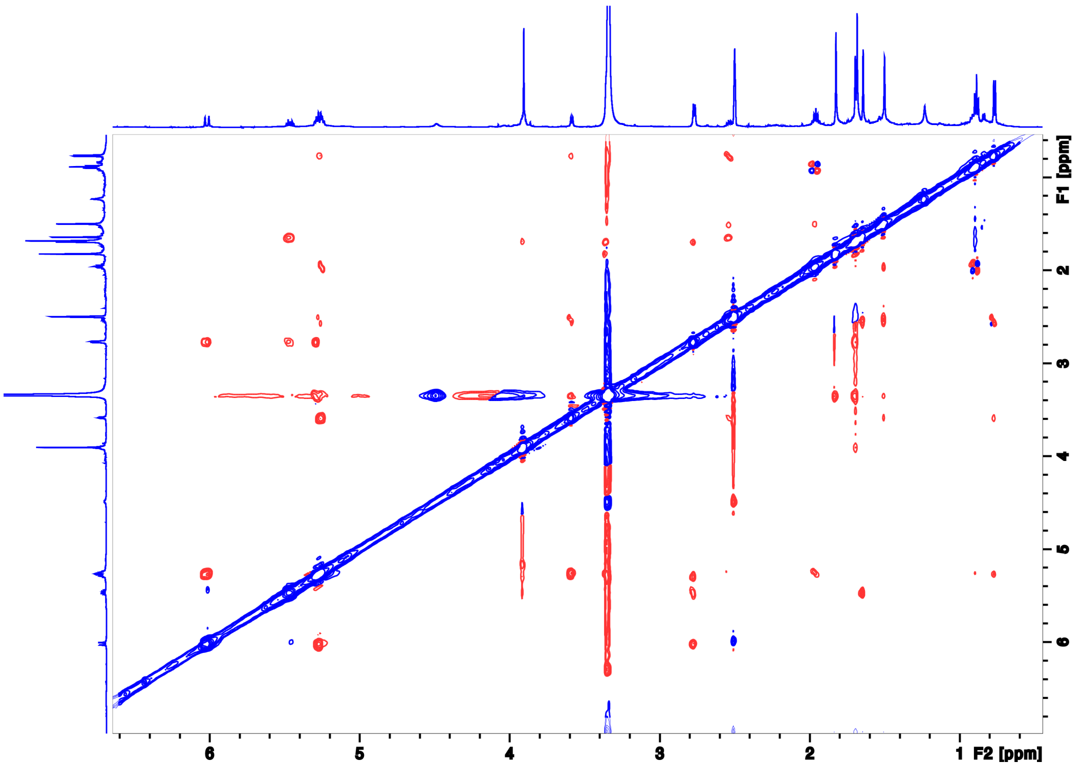

Figure S10. ROESY NMR (DMSO-d_6) spectrum of goondapyrone A (**1**).

Mass Spectrum Molecular Formula Report

Analysis Info

Analysis Name	D:\Data\Shengbin\Shengbin_S4S196A10_30.d	Acquisition Date	2/27/2023 1:34:33 PM
Method	tune-medhigh_AP.m	Operator	a.salim
Sample Name	Shengbin_S4S196A10_30	Instrument / Ser#	micrOTOF 213750.00232
Comment			

Acquisition Parameter

Source Type	ESI	Ion Polarity	Positive	Set Nebulizer	0.5 Bar
Focus	Not active			Set Dry Heater	180 °C
Scan Begin	100 m/z	Set Capillary	4500 V	Set Dry Gas	5.0 l/min
Scan End	1500 m/z	Set End Plate Offset	-500 V	Set Divert Valve	Source

Generate Molecular Formula Parameter

Formula, min.		
Formula, max.		
Measured m/z	Tolerance	Charge
Check Valence	Minimum	Maximum
Nirogen Rule	Electron Configuration	
Filter H/C Ratio	Minimum	Maximum
Estimate Carbon		

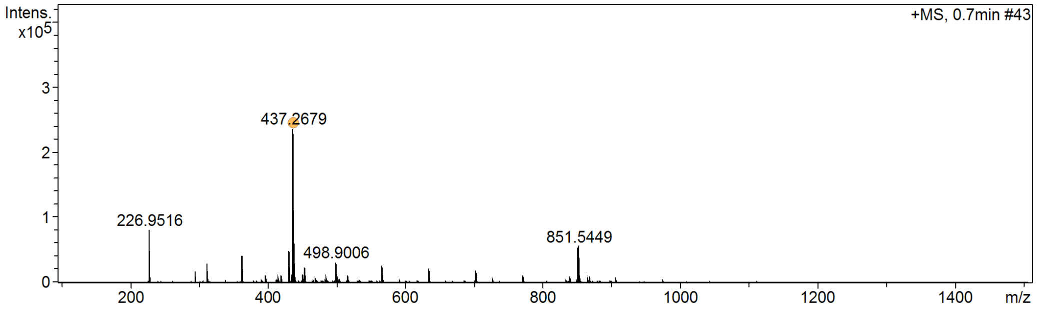

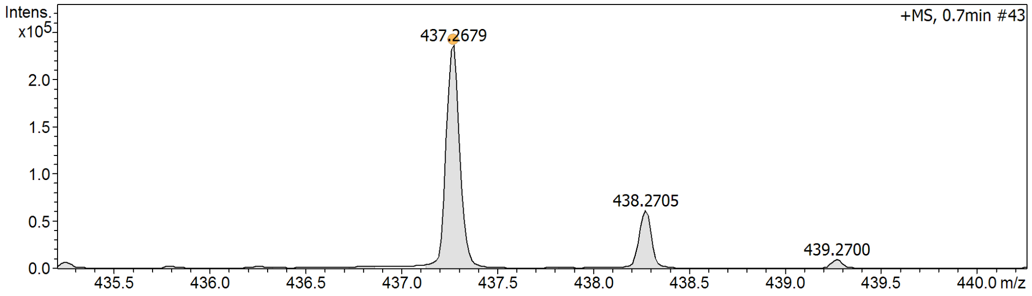

Meas. m/z	#	Ion Formula	m/z	err [ppm]	mSigma	# Sigma	Score	rdb	e⁻ Conf	N-Rule
437.2679	1	C26H38NaO4	437.2662	-3.7	14.1	1	59.65	7.5	even	ok

Bruker Compass DataAnalysis 4.1 printed: 3/27/2023 10:17:11 AM Page 1 of 1

Figure S11. HRESIMS spectrum for goondapyrone A (**1**).

2.2 Goondapyrone B (2)

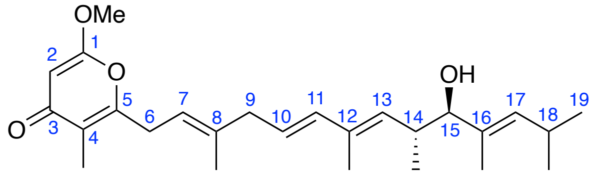

Table S3. 1D and 2D NMR (DMSO-d_6) data for goondapyrone B (**2**).

**Pos.**	δ_C	δ_H, mult (J in Hz)	COSY	^1H-^{13}C HMBC	ROESY
1	167.0				
2	87.7	5.52, s		1, 3, 4	1-OMe
3	180.0				
4	117.4				
5	158.9				
6	29.5	3.30, d *(7.3)*	7, 8-CH_3	5, 7, 8	7, 4-CH_3, 8-CH_3
7	117.9	5.22, t *(7.3)*	6, 8-CH_3	6, 9, 8-CH_3	6, 9
8	137.7				
9	42.1	2.76, d *(7.4)*	10	7, 8, 10, 8-CH_3	7, 10, 11, 8-CH_3
10	123.9	5.44, dt *(15.6, 7.5)*	9, 11	8, 9, 12	9, 12-CH_3
11	136.6	6.02, d *(15.6)*	9, 10	9, 12, 13, 12-CH_3	9, 13
12	132.0				
13	135.3	5.26, d *(9.4)*	14, 12-CH_3	11, 14, 15, 12-CH_3, 14-CH_3	11, 14, 14-CH_3, 15
14	36.1	2.53[A], m	13, 15, 14-CH_3	12, 13, 15, 14-CH_3	13, 15, 12-CH_3, 14-CH_3, 16-CH_3
15	80.2	3.56, dd *(7.2, 4.1)*	14, 15-OH	13, 17	13, 14, 17, 14-CH_3, 16-CH_3, 15-OH
16	134.4				
17	133.0	5.07, d *(9.1)*	18, 16-CH_3	15, 18, 19, 16-CH_3, 18-CH_3	15, 18, 19, 18-CH_3
18	26.2	2.47[A], m	17, 19, 18-CH_3	16, 17, 19, 18-CH_3	17, 19, 16-CH_3, 18-CH_3
19	23.0	0.89, d *(5.9)*	18	17, 18, 18-CH_3	17, 18
15-OH		4.49, d (*4.1*)	15	14	13, 14, 15, 17, 14-CH_3, 16-CH_3
4-CH_3	9.1	1.80, s	6	3, 4, 5	6
8-CH_3	16.3	1.66, s	6, 7	7, 8, 9	9
12-CH_3	12.7	1.64, s	13	11, 12, 13	10, 14
14-CH_3	17.9	0.78, d *(6.6)*	14	13, 14, 15	13, 14, 15
16-CH_3	11.6	1.51, s	17	15, 16, 17	14, 15, 18, 15-OH
18-CH_3	22.8	0.87, d *(6.1)*	18	17, 18, 19	17, 18
1-OCH_3	56.4	3.83, s		1	2

[A] overlapping signals

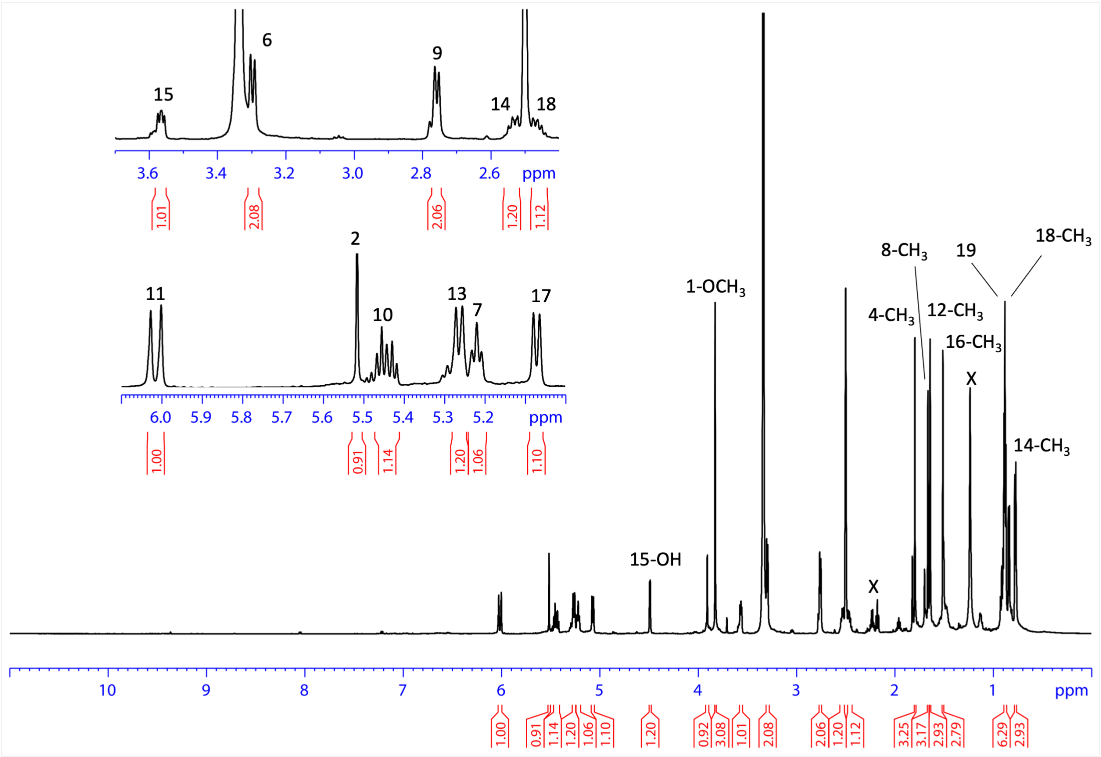

Figure S12. ^{1}H NMR (DMSO-d_6) spectrum of goondapyrone B (**2**).

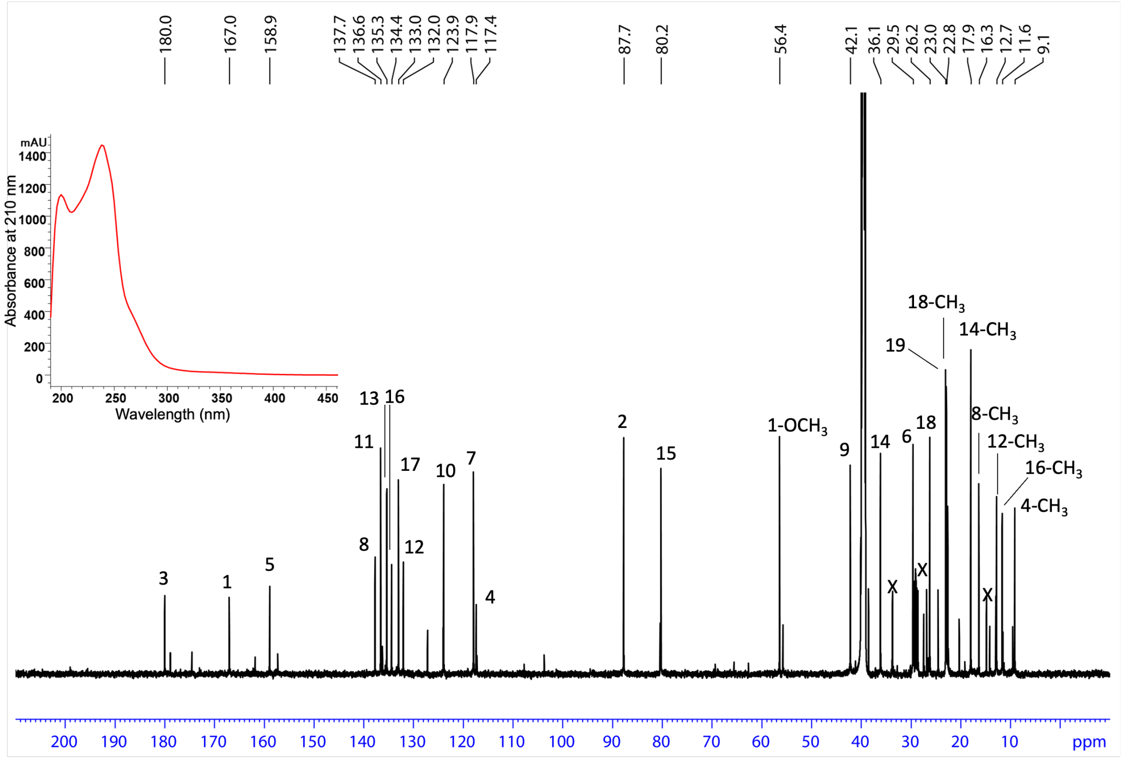

Figure S13. ^{13}C NMR (DMSO-d_6) and UV-vis (inset) spectra of goondapyrone B (**2**).

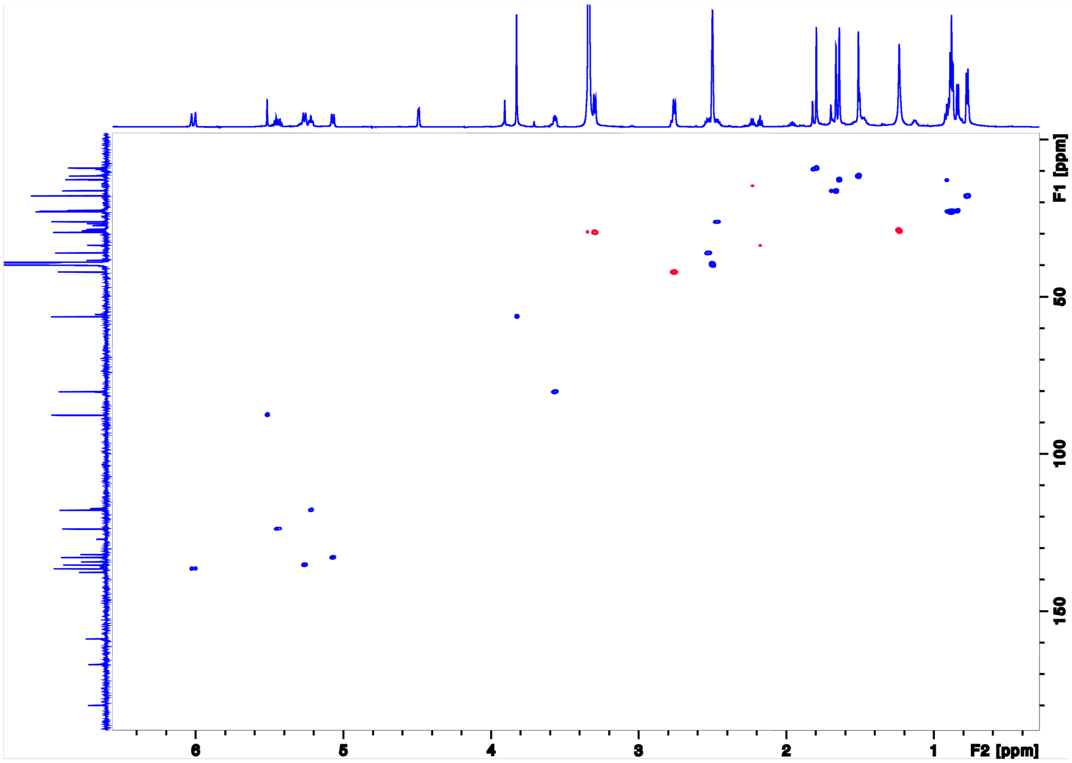

Figure S14. HSQC NMR (DMSO-d_6) spectrum of goondapyrone B (**2**).

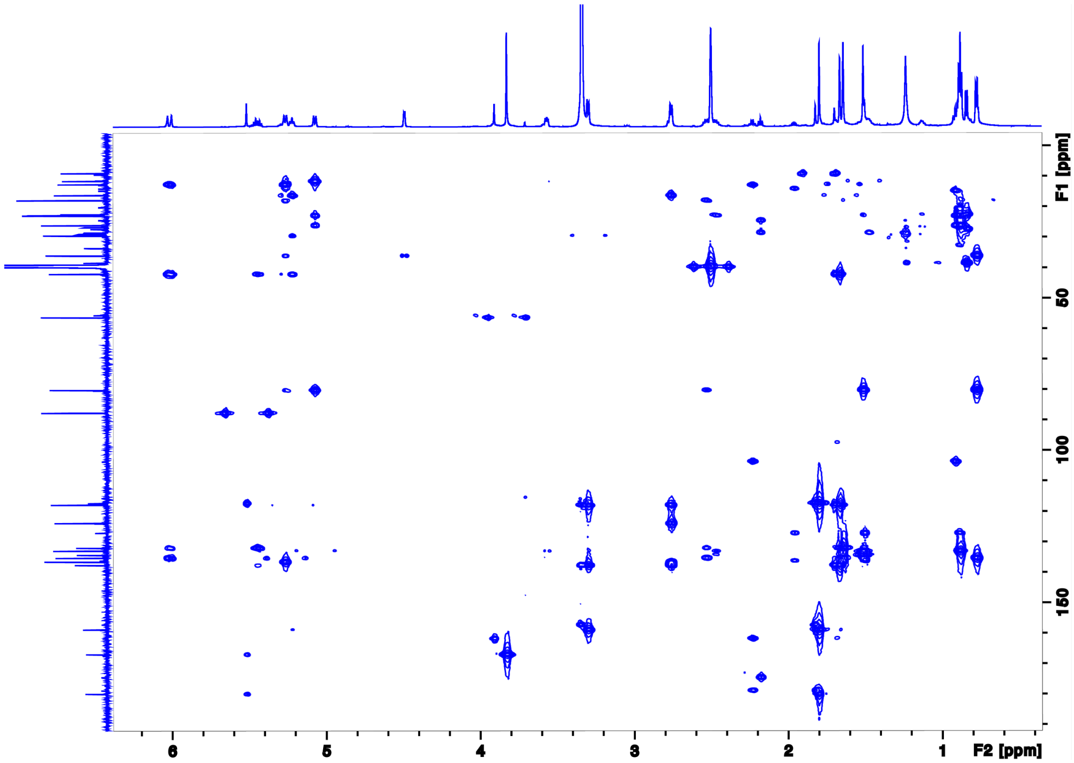

Figure S15. HMBC NMR (DMSO-d_6) spectrum of goondapyrone B (**2**).

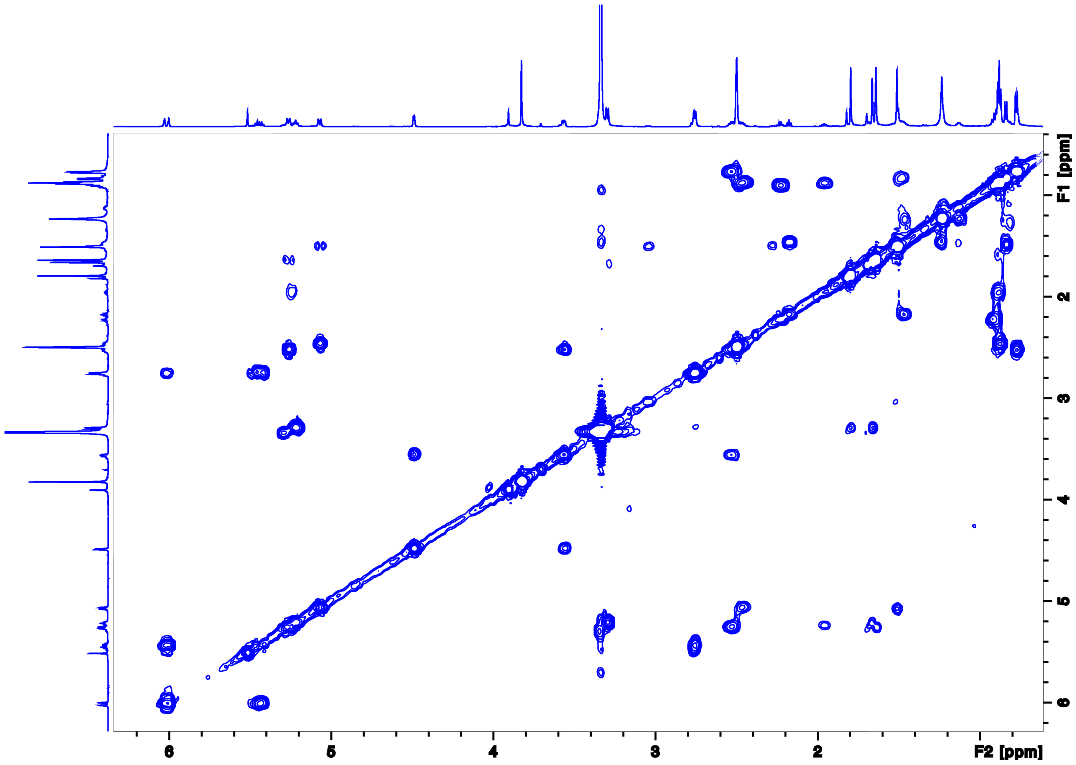

Figure S16. COSY NMR (DMSO-d_6) spectrum of goondapyrone B (**2**).

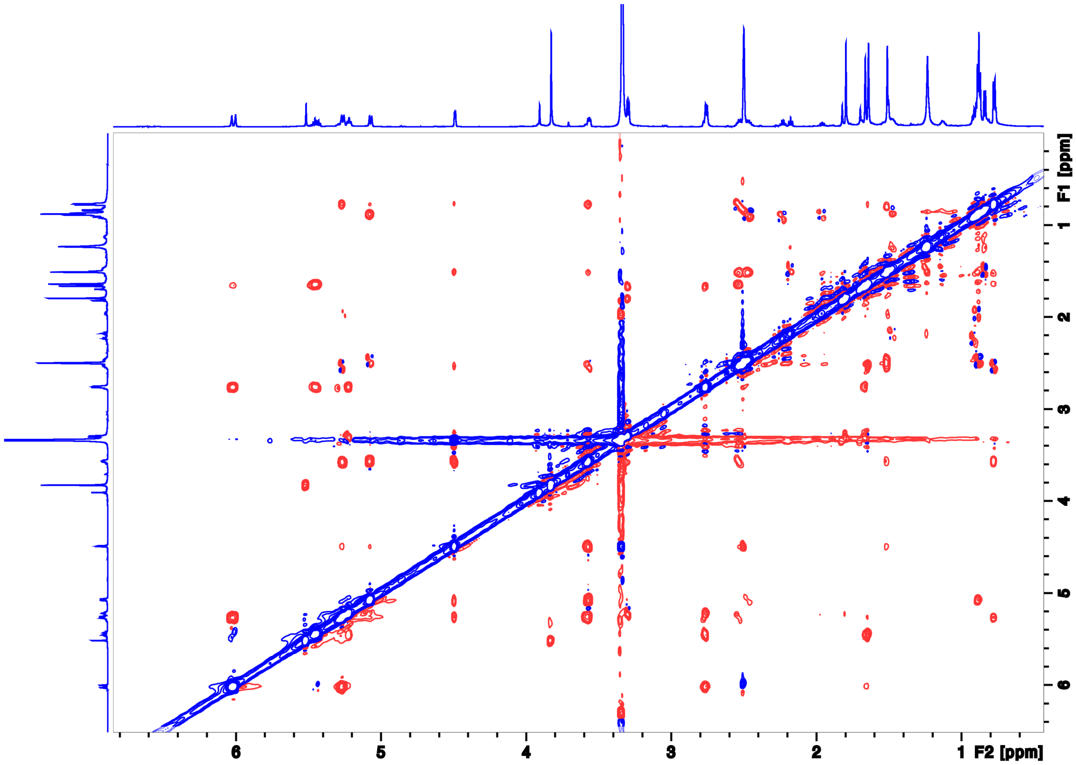

Figure S17. ROESY NMR (DMSO-d_6) spectrum of goondapyrone B (**2**).

Mass Spectrum Molecular Formula Report

Analysis Info

Analysis Name	D:\Data\Shengbin\Shengbin_S4S196A10_31.d	Acquisition Date	2/27/2023 1:37:52 PM
Method	tune-medhigh_AP.m	Operator	a.salim
Sample Name	Shengbin_S4S196A10_31	Instrument / Ser#	micrOTOF 213750.00232
Comment			

Acquisition Parameter

Source Type	ESI	Ion Polarity	Positive	Set Nebulizer	0.5 Bar
Focus	Not active			Set Dry Heater	180 °C
Scan Begin	100 m/z	Set Capillary	4500 V	Set Dry Gas	5.0 l/min
Scan End	1500 m/z	Set End Plate Offset	-500 V	Set Divert Valve	Source

Generate Molecular Formula Parameter

Formula, min.		
Formula, max.		
Measured m/z	Tolerance	Charge
Check Valence	Minimum	Maximum
Nirogen Rule	Electron Configuration	
Filter H/C Ratio	Minimum	Maximum
Estimate Carbon		

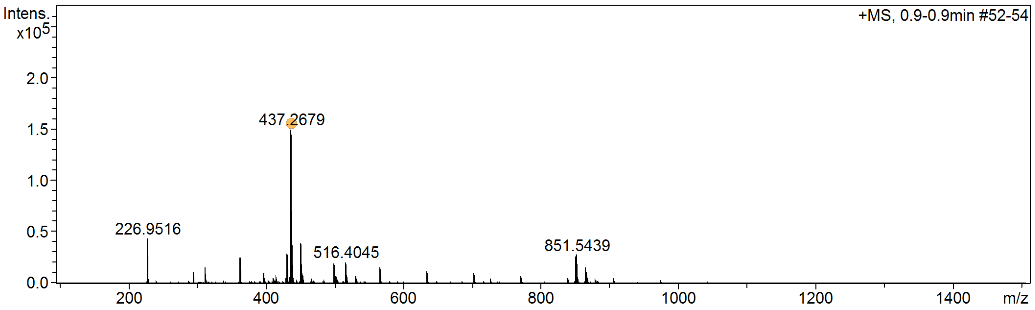

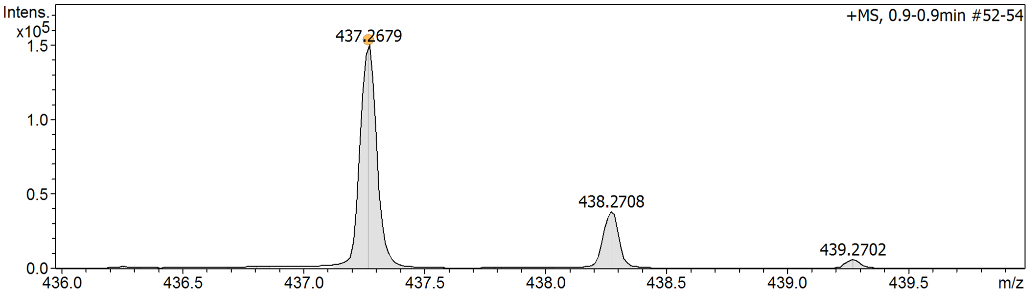

Meas. m/z	#	Ion Formula	m/z	err [ppm]	mSigma	# Sigma	Score	rdb	e⁻ Conf	N-Rule
437.2679	1	C26H38NaO4	437.2662	3.8	15.4	1	59.86	7.5	even	ok

Bruker Compass DataAnalysis 4.1 printed: 3/27/2023 10:33:15 AM Page 1 of 1

Figure S18. HRESIMS spectrum for goondapyrone B (**2**).

2.3 Goondapyrone C (3)

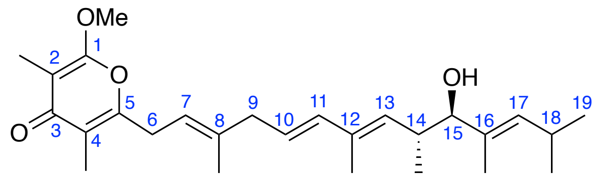

Table S4. 1D and 2D NMR (DMSO-d_6) data for goondapyrone C (**3**).

**Pos.**	δ_C	δ_H, mult (*J* in Hz)	COSY	^{1}H-^{13}C HMBC	ROESY
1	161.8				
2	97.5				
3	179.4				
4	116.8				
5	157.2				
6	29.4	3.35, d *(7.1)*	7, 8-CH_3	5, 7, 8	7, 4-CH_3, 8-CH_3
7	117.8	5.28, t *(7.0)*	6	6, 9, 24	6, 9
8	137.7				
9	42.1	2.77, d *(6.9)*	10	7, 8, 10, 8-CH_3	7, 10, 11, 8-CH_3
10	123.9	5.46, dt *(15.5, 6.9)*	9, 11	9, 12, 13	9, 11, 12-CH_3
11	136.6	6.01, d *(15.5)*	9, 10	9, 12, 13, 12-CH_3	9, 10, 13
12	132.0				
13	135.3	5.25, d *(9.3)*	14	11, 15, 12-CH_3	11, 15
14	36.1	2.54^A, m	13, 15, 14-CH_3	12, 13, 15, 14-CH_3	15, 12-CH_3, 14-CH_3, 16-CH_3
15	80.2	3.56, d *(6.9)*	14	14, 16, 17, 14-CH_3, 16-CH_3	13, 14, 17, 14-CH_3, 16-CH_3
16	134.4				
17	133.0	5.07, d *(9.1)*	18, 16-CH_3	15, 18, 19, 16-CH_3, 18-CH_3	15, 19, 18-CH_3
18	26.1	2.46^A, m	17, 19, 18-CH_3	16, 17, 19, 18-CH_3	19, 16-CH_3, 18-CH_3
19	23.0	0.88, d *(6.2)*	18	17, 18, 18-CH_3	17, 18
15-OH					
2-CH_3	6.8	1.68, s		1, 2, 3	
4-CH_3	9.5	1.82, s	6	3, 4, 5	6
8-CH_3	16.3	1.69, s	6, 7	7, 8, 9	6, 9
12-CH_3	12.7	1.64, s	13	11, 12, 13	10, 14
14-CH_3	17.9	0.77, d *(6.9)*	14	13, 14, 15	14, 15
16-CH_3	11.6	1.51, s	17	15, 16, 17	14, 15, 18
18-CH_3	22.8	0.87, d *(6.2)*	18	17, 18, 19	17, 18
1-OCH_3	55.6	3.91, s		1	

^{A} overlapping signals

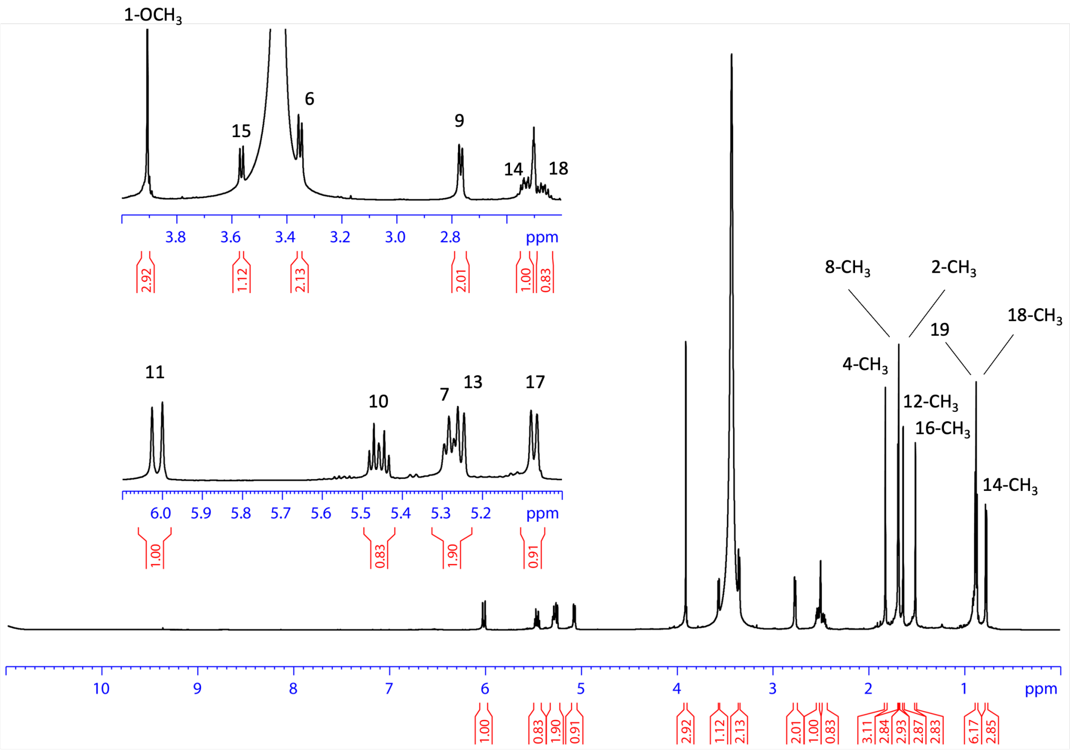

Figure S19. ^{1}H NMR (DMSO-d_6) spectrum of goondapyrone C (**3**).

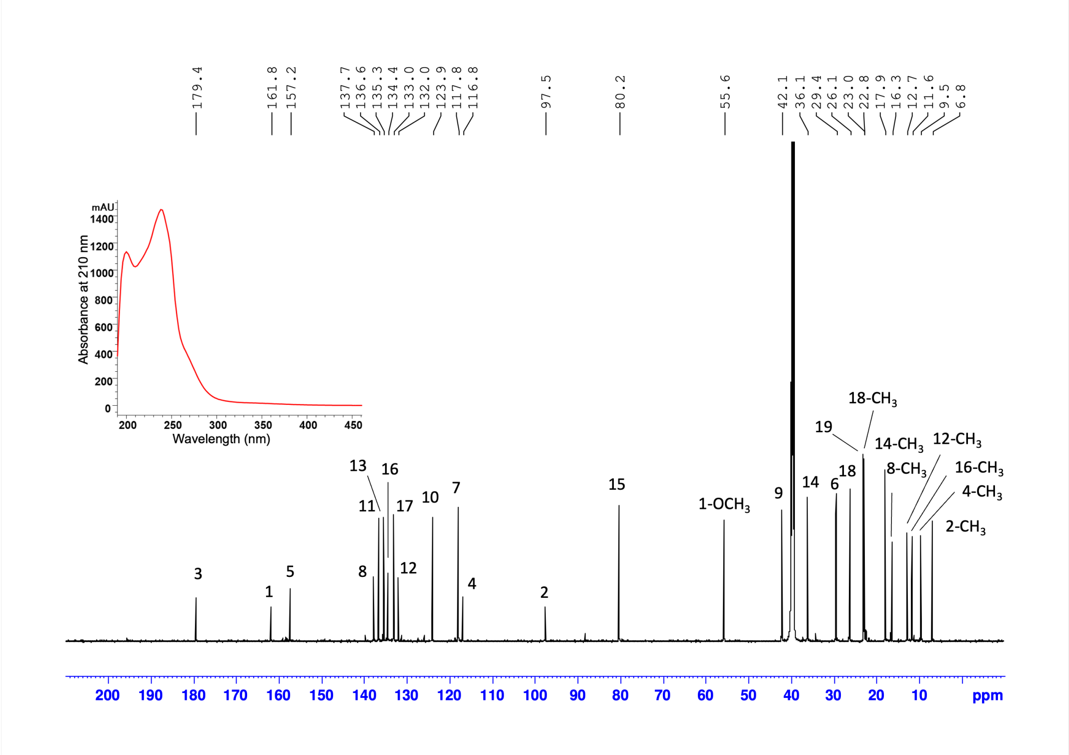

Figure S20. ^{13}C NMR (DMSO-d_6) and UV-vis (inset) spectra of goondapyrone C (**3**).

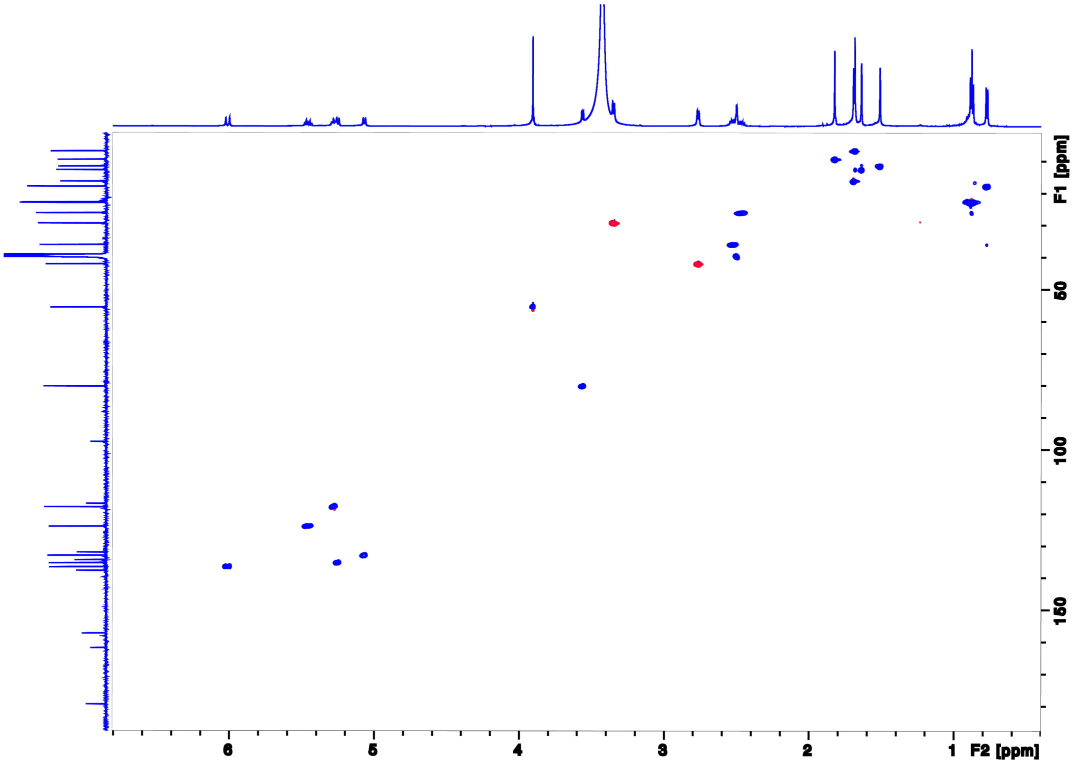

Figure S21. HSQC NMR (DMSO-d_6) spectrum of goondapyrone C (**3**).

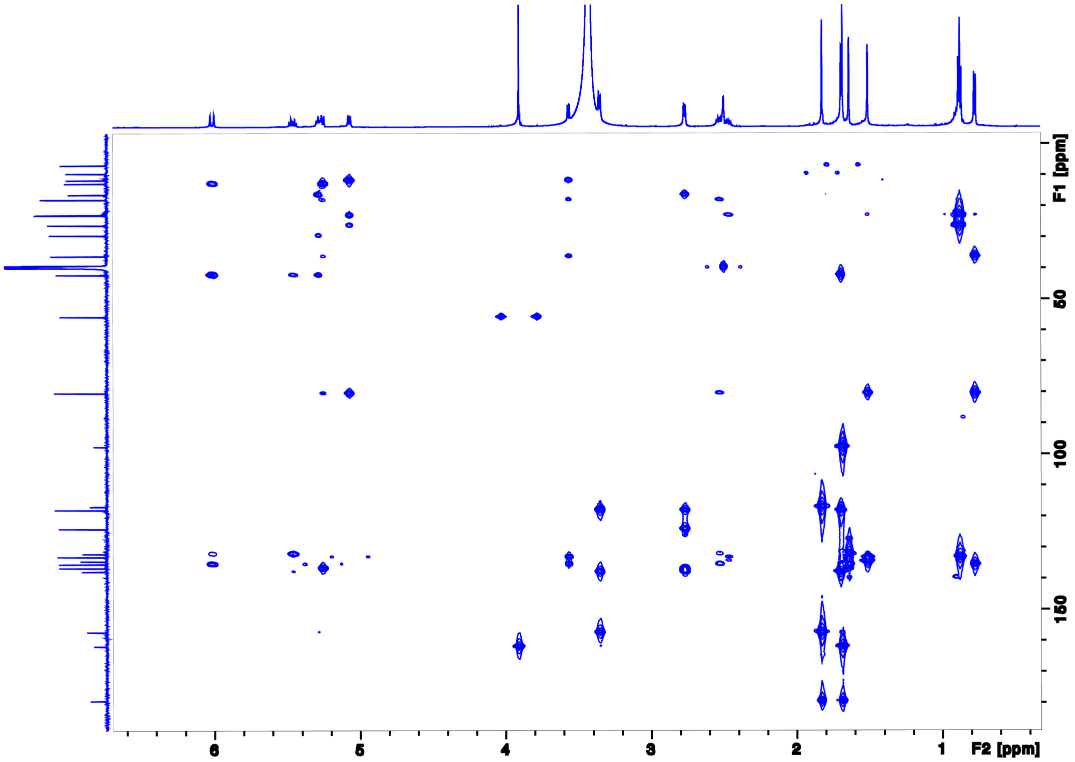

Figure S22. HMBC NMR (DMSO-d_6) spectrum of goondapyrone C (**3**).

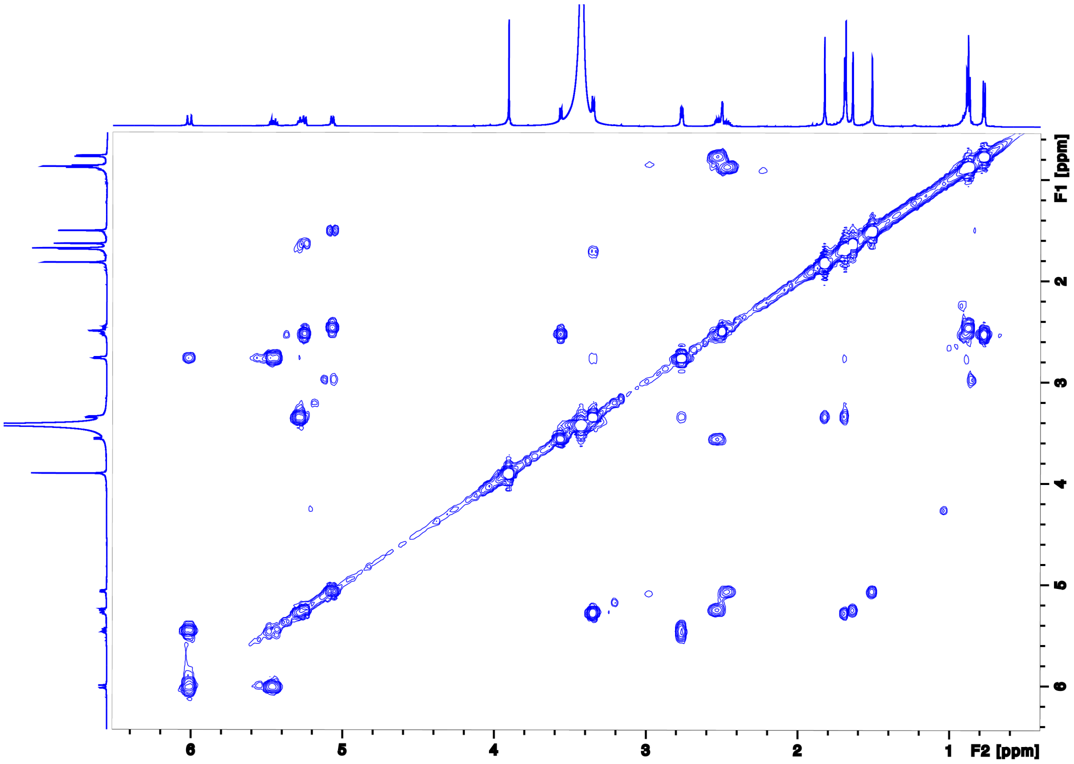

Figure S23. COSY NMR (DMSO-d_6) spectrum of goondapyrone C (**3**).

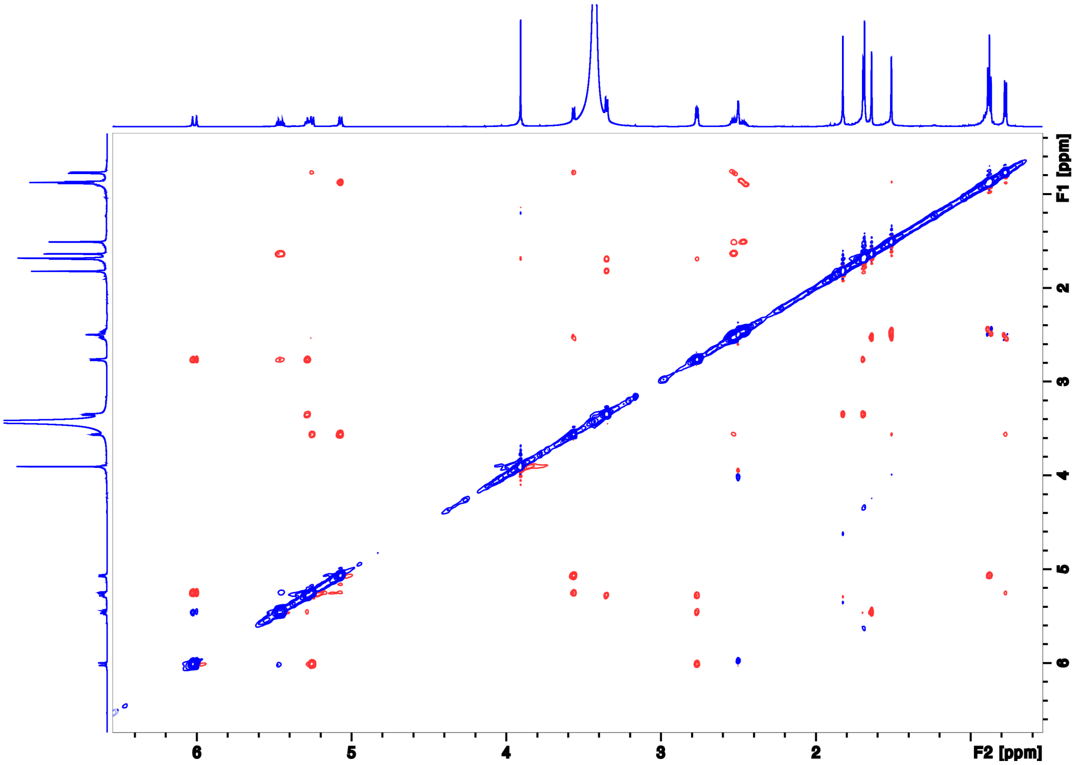

Figure S24. ROESY NMR (DMSO-d_6) spectrum of goondapyrone C (**3**).

Mass Spectrum Molecular Formula Report

Analysis Info

Analysis Name	D:\Data\Shengbin\Shengbin_S4S-00196A10_8-4.d	Acquisition Date	1/24/2022 9:42:41 AM
Method	tune-medhigh_AP.m	Operator	a.salim
Sample Name	Shengbin_S4S-00196A10_8-4	Instrument / Ser#	micrOTOF 213750.00232
Comment			

Acquisition Parameter

Source Type	ESI	Ion Polarity	Positive	Set Nebulizer	0.5 Bar
Focus	Not active			Set Dry Heater	180 °C
Scan Begin	100 m/z	Set Capillary	4500 V	Set Dry Gas	5.0 l/min
Scan End	1500 m/z	Set End Plate Offset	-500 V	Set Divert Valve	Source

Generate Molecular Formula Parameter

Formula, min.		
Formula, max.		
Measured m/z	Tolerance	Charge
Check Valence	Minimum	Maximum
Nirogen Rule	Electron Configuration	
Filter H/C Ratio	Minimum	Maximum
Estimate Carbon		

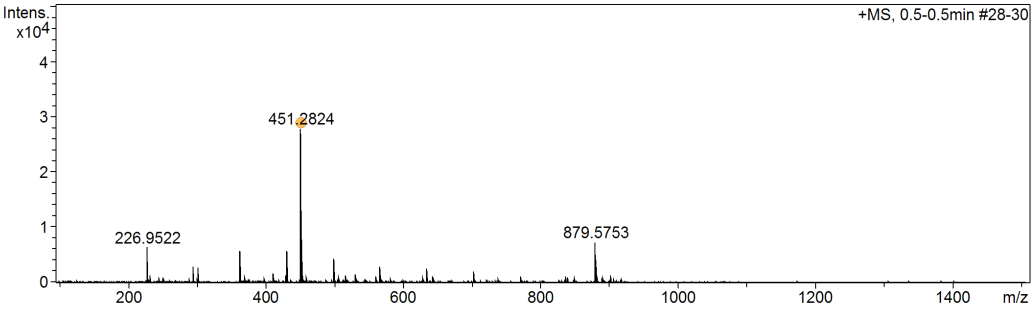

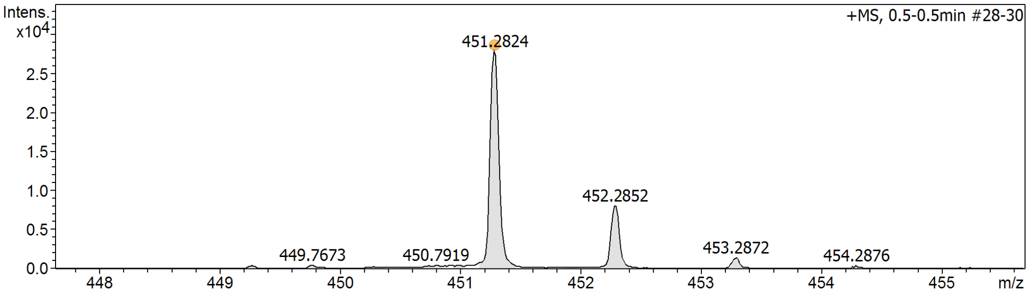

Meas. m/z	#	Ion Formula	m/z	err [ppm]	mSigma	# Sigma	Score	rdb	e¯ Conf	N-Rule
451.2824	1	C27H40NaO4	451.2819	-1.1	5.5	1	100.00	7.5	even	ok

Bruker Compass DataAnalysis 4.1 printed: 3/27/2023 11:08:39 AM Page 1 of 1

Figure S25. HRESIMS spectrum for goondapyrone C (**3**).

2.4 Goondapyrone D (4)

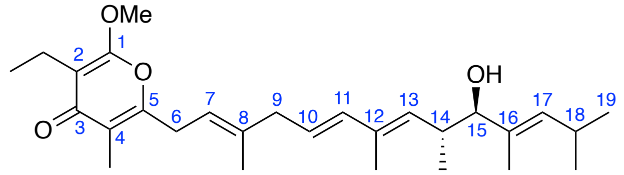

Table S5. 1D and 2D NMR (600 MHz, DMSO-d_6) data for goondapyrone D (**4**).

**Pos.**	δ_C	δ_H, mult (*J* in Hz)	COSY	^{1}H-^{13}C HMBC	ROESY
1	161.8				
2	103.7				
3	178.8				
4	117.2				
5	157.3				
6	29.4	3.34, d *(7.3)*	7, 4-CH_3, 8-CH_3	5, 7, 8	
7	117.8	5.28, t *(7.3)*	6, 8-CH_3	6, 9, 8-CH_3	9
8	137.7				
9	42.1	2.76, d *(7.0)*	10	7, 8, 10, 8-CH_3	7, 10, 11, 8-CH_3
10	124.0	5.46, dt *(15.5, 7.0)*	9, 11	9, 12	9, 12-CH_3
11	136.6	6.01, d *(15.5)*	9, 10	9, 12, 13, 12-CH_3	9, 10
12	132.0				
13	135.3	5.25, d *(9.5)*	14, 12-CH_3	11, 15, 12-CH_3, 14-CH_3	11, 14, 15, 14-CH_3
14	36.1	2.53[A], m	13, 15, 14-CH_3	12, 13, 15, 14-CH_3	15, 16-CH_3
15	80.2	3.56, dd *(6.8, 4.0)*	14, 15-OH	13, 14, 17, 16-CH_3	13, 14, 17, 14-CH_3, 16-CH_3
16	134.4				
17	133.0	5.07, d *(9.1)*	18, 16-CH_3	18, 19, 20, 16-CH_3	15, 18, 19, 18-CH_3
18	26.2	2.46[A], m	17, 19, 18-CH_3	16, 17, 19, 18-CH_3	19, 18-CH_3
19	23.0	0.88, d *(6.3)*	18	17, 18, 18-CH_3	17, 18
15-OH		4.50, d *(4.0)*	15	14	15
4-CH_3	9.5	1.82, s	6	3, 4, 5	
8-CH_3	16.3	1.69, s	6, 7	7, 8, 9	9
12-CH_3	12.7	1.64, s	13	11, 12, 13	10, 14
14-CH_3	17.9	0.77, d *(6.7)*	14	13, 14, 15	13, 14, 15
16-CH_3	11.6	1.51, s	17	15, 16, 17	18
18-CH_3	22.8	0.87, d *(6.3)*	18	17, 18, 19	17, 18
1-OCH_3	55.7	3.90, s		1	
2-$\underline{CH_2}CH_3$	14.8	2.23, q *(7.5)*	2-$CH_2C\underline{H_3}$	1, 2, 3, 2-$CH_2\underline{C}H_3$	2-$CH_2C\underline{H_3}$
2-$CH_2\underline{CH_3}$	12.9	0.91, t *(7.5)*	2-$C\underline{H_2}CH_3$	2, 2-$\underline{C}H_2CH_3$	2-$C\underline{H_2}CH_3$

[A] overlapping signals

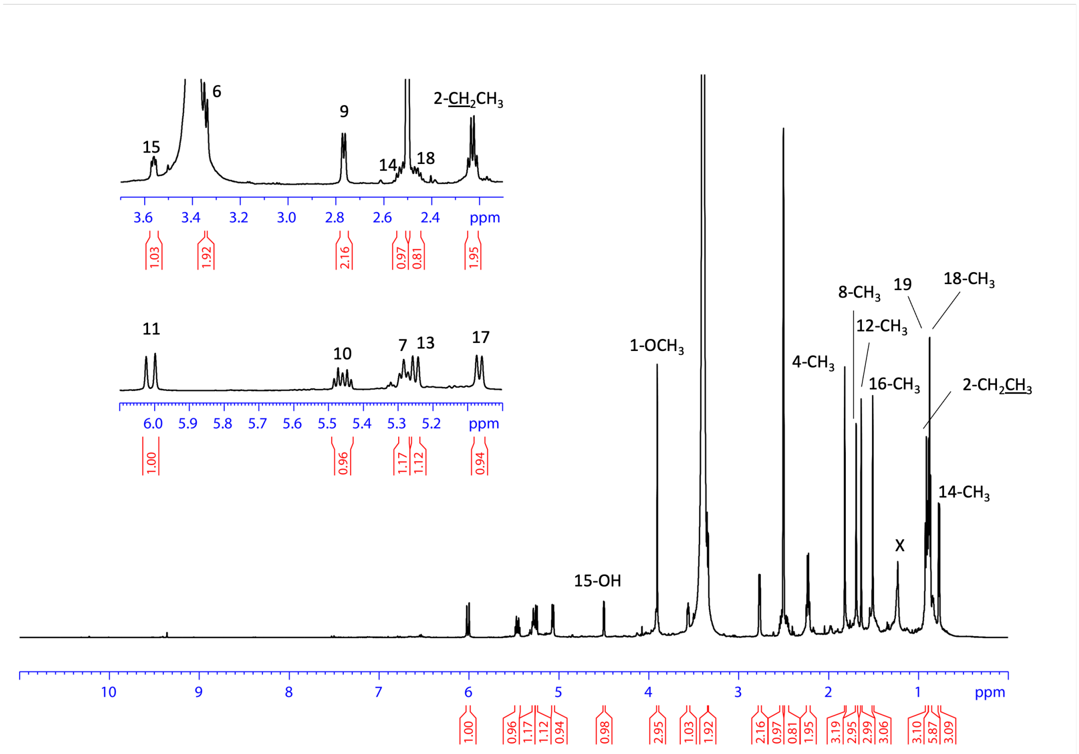

Figure S26. ^{1}H NMR (DMSO-d_6) spectrum of goondapyrone D (**4**).

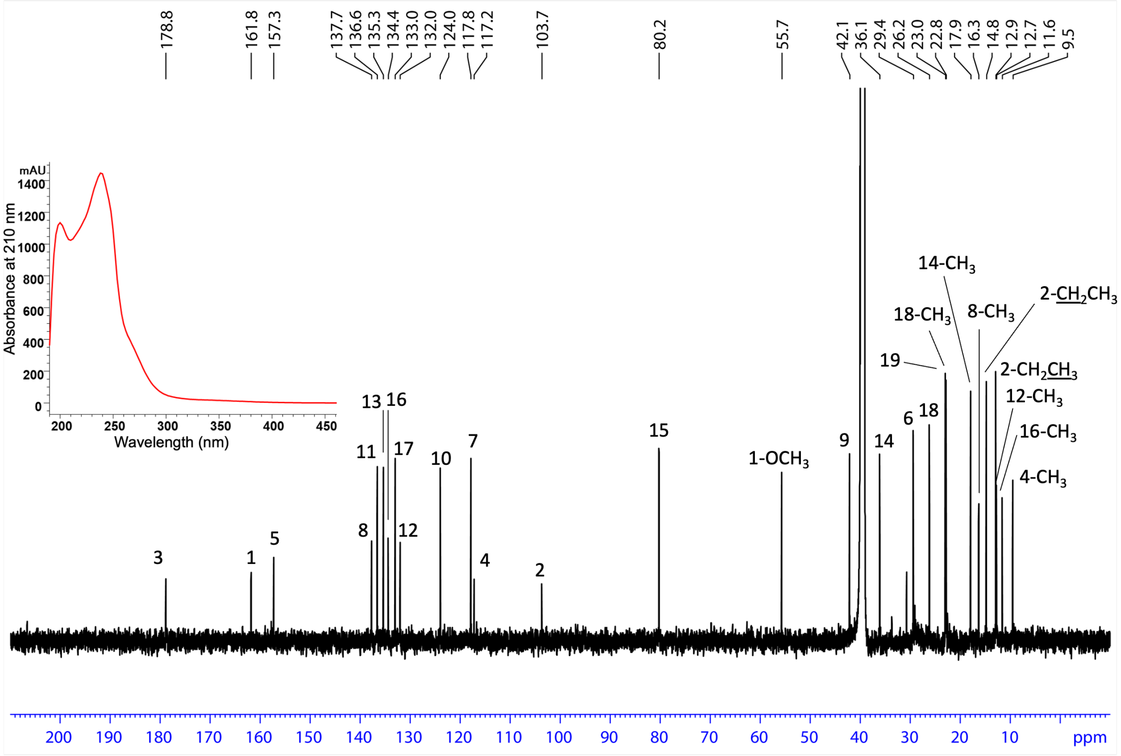

Figure S27. ^{13}C NMR (DMSO-d_6) and UV-vis (inset) spectra of goondapyrone D (**4**).

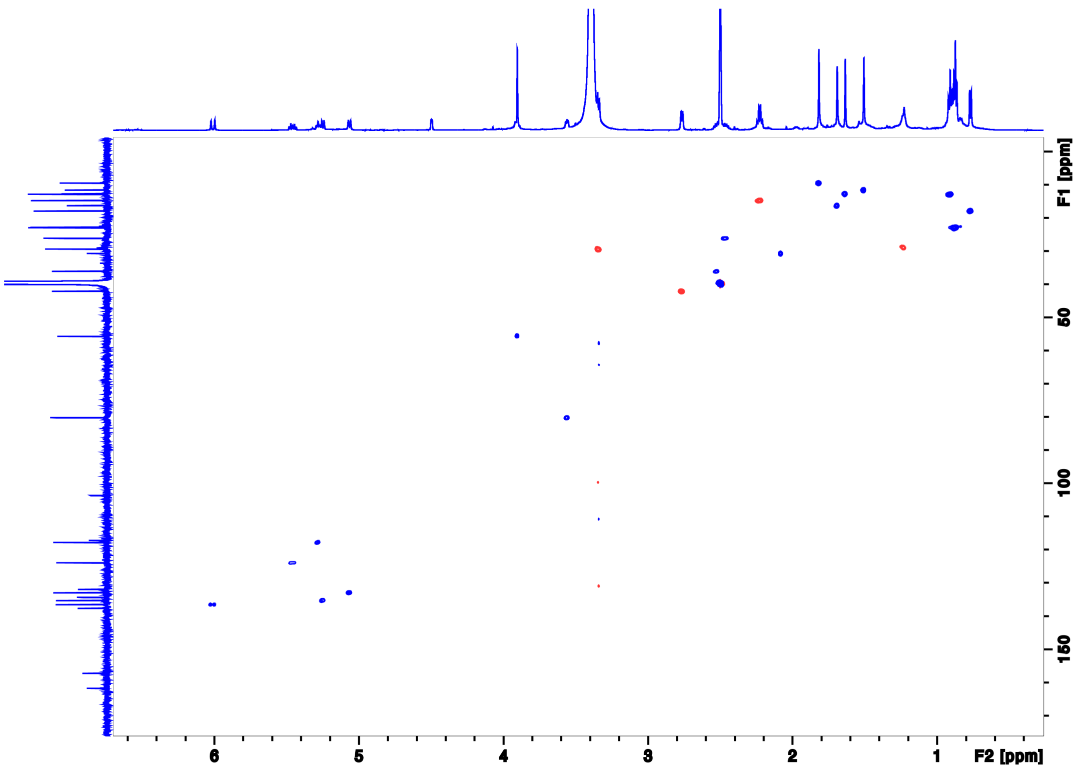

Figure S28. HSQC NMR (DMSO-d_6) spectrum of goondapyrone D (**4**).

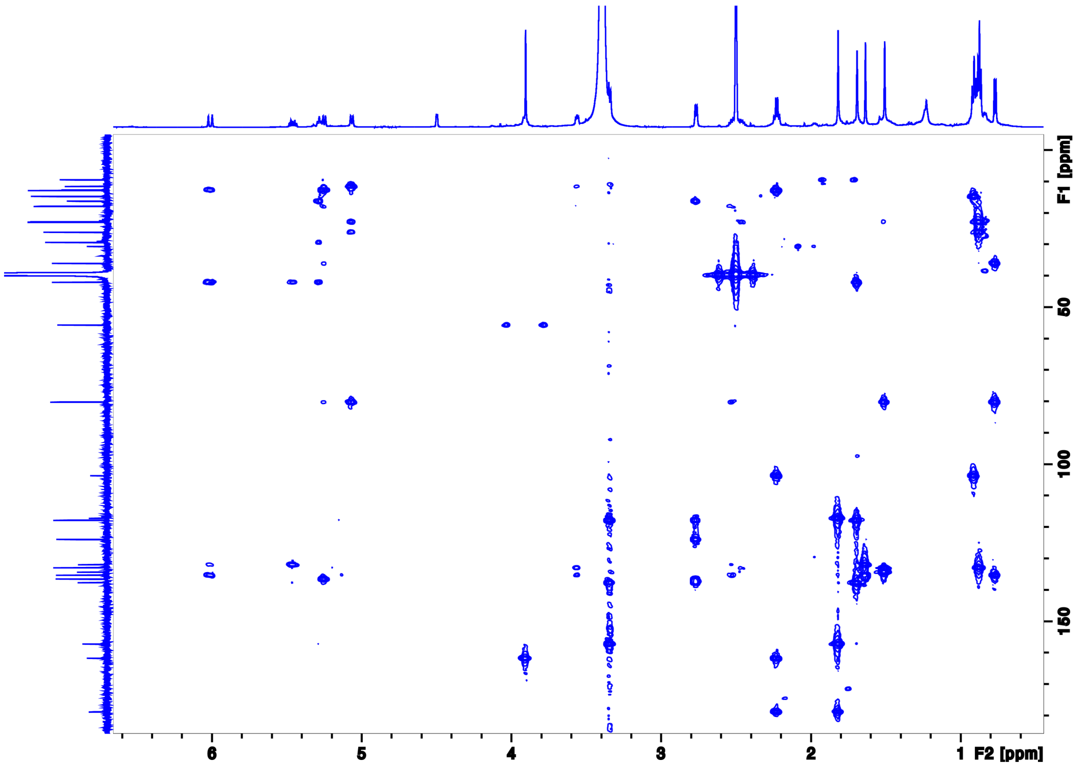

Figure S29. HMBC NMR (DMSO-d_6) spectrum of goondapyrone D (**4**).

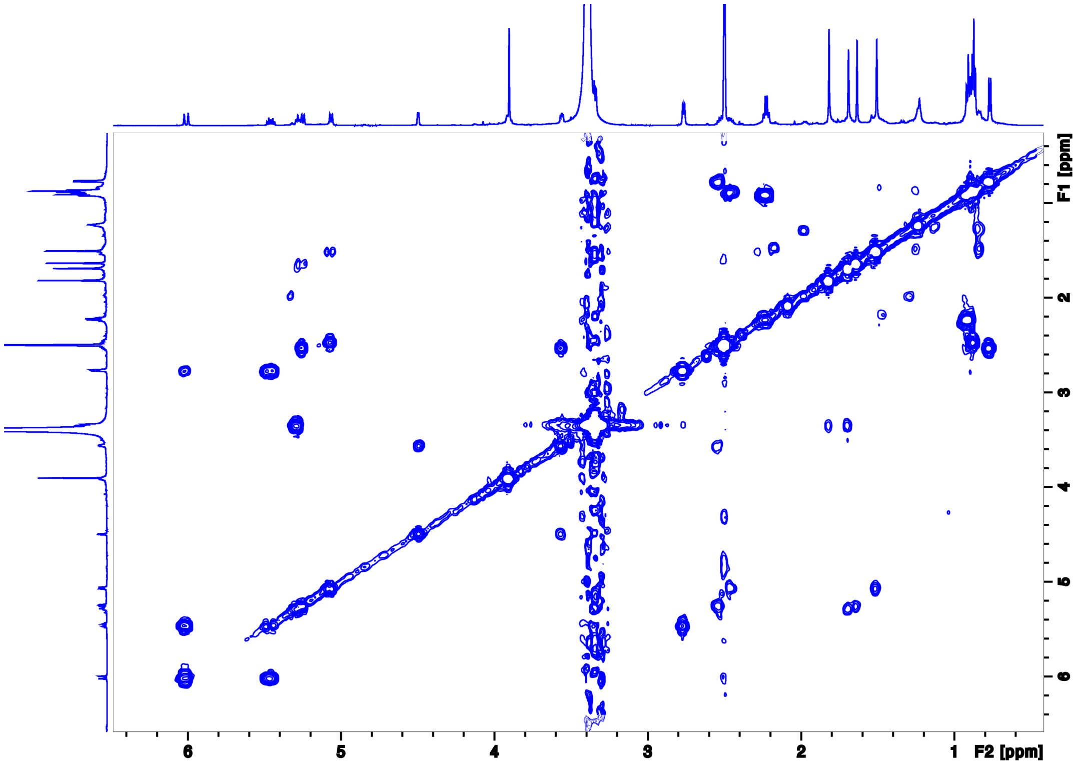

Figure S30. COSY NMR (DMSO-d_6) spectrum of goondapyrone D (**4**).

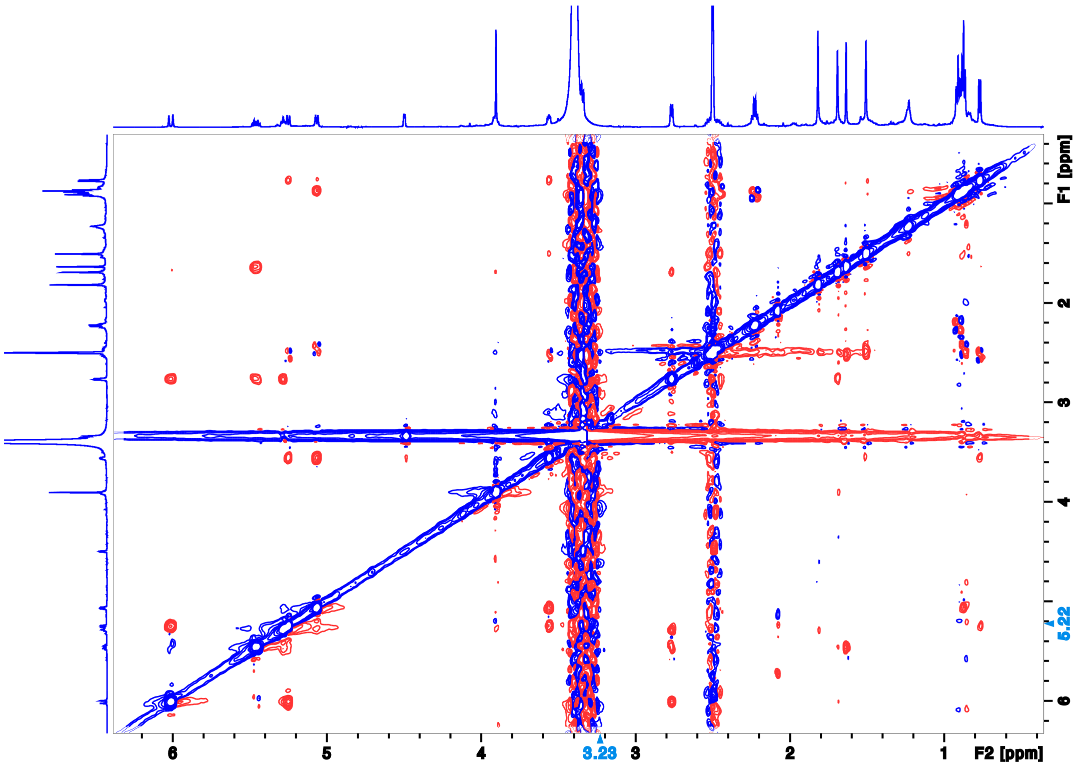

Figure S31. ROESY NMR (DMSO-d_6) spectrum of goondapyrone D (**4**).

Mass Spectrum Molecular Formula Report

Analysis Info

Analysis Name	D:\Data\Shengbin\Shengbin_S4S-00196A10_12.d	Acquisition Date	1/24/2022 11:27:12 AM
Method	tune-medhigh_AP.m	Operator	a.salim
Sample Name	Shengbin_S4S-00196A10_12	Instrument / Ser#	micrOTOF 213750.00232
Comment			

Acquisition Parameter

Source Type	ESI	Ion Polarity	Positive	Set Nebulizer	0.5 Bar
Focus	Not active			Set Dry Heater	180 °C
Scan Begin	100 m/z	Set Capillary	4500 V	Set Dry Gas	5.0 l/min
Scan End	1500 m/z	Set End Plate Offset	-500 V	Set Divert Valve	Source

Generate Molecular Formula Parameter

Formula, min.		
Formula, max.		
Measured m/z	Tolerance	Charge
Check Valence	Minimum	Maximum
Nirogen Rule	Electron Configuration	
Filter H/C Ratio	Minimum	Maximum
Estimate Carbon		

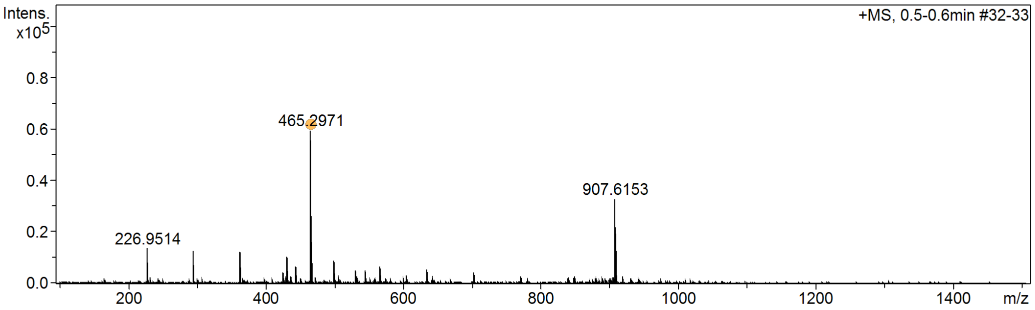

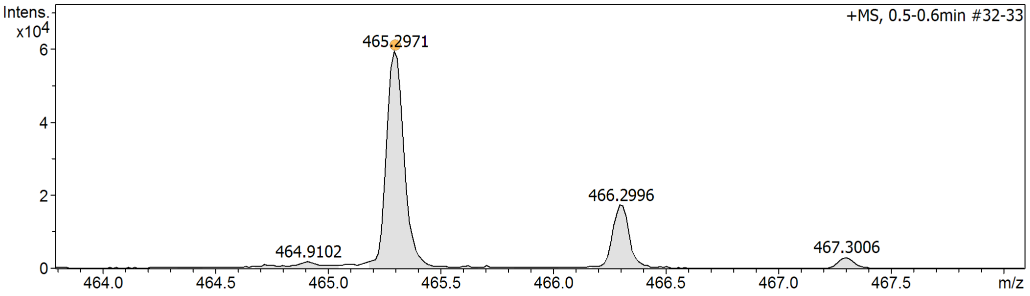

Meas. m/z	#	Ion Formula	m/z	err [ppm]	mSigma	# Sigma	Score	rdb	e⁻ Conf	N-Rule
465.2971	1	C28H42NaO4	465.2975	0.9	9.1	1	100.00	7.5	even	ok

Bruker Compass DataAnalysis 4.1 printed: 3/27/2023 11:22:28 AM Page 1 of 1

Figure S32. HRESIMS spectrum for goondapyrone D (**4**).

2.5 Goondapyrone E (5)

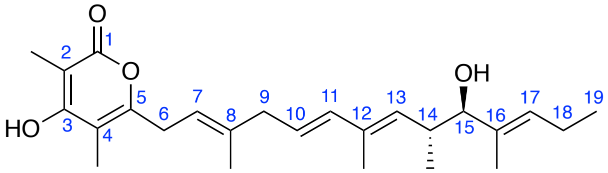

Table S6. 1D and 2D NMR (DMSO-d_6) data for goondapyrone E (**5**).

**Pos.**	δ_C	δ_H, mult (J in Hz)	COSY	^{1}H-^{13}C HMBC	ROESY
1	164.4				
2	97.3				
3	164.7				
4	106.4				
5	156.7				
6	29.7	3.21, d *(6.9)*	7, 8-CH_3	4, 5, 7, 8	7, 4-CH_3, 8-CH_3
7	118.6	5.19, t *(6.9)*	6, 8-CH_3	6, 9, 8-CH_3	6, 9
8	136.9				
9	42.2	2.75, d *(6.9)*	10	8, 10, 11, 8-CH_3	7, 10, 11, 8-CH_3
10	124.0	5.44, dt *(15.5, 6.9)*	9, 11	8, 12	9, 11, 12-CH_3
11	136.6	6.02, d *(15.5)*	9, 10	9, 12, 13, 12-CH_3	9, 10, 13, 12-CH_3
12	132.1				
13	135.4	5.28, d *(9.3)*	14	11, 14, 12-CH_3, 14-CH_3	11, 14, 14-CH_3
14	36.1	2.54, m	13, 15, 14-CH_3	12, 13, 15, 14-CH_3	15, 12-CH_3, 14-CH_3, 16-CH_3
15	80.4	3.58, d *(7.1)*	14	13, 14, 16, 14-CH_3, 16-CH_3	13, 14, 14-CH_3, 16-CH_3
16	136.2				
17	127.1	5.25, t *(7.1)*	18, 16-CH_3	15, 18, 16-CH_3	15, 18
18	20.2	1.96, m	17, 19	16, 17, 19	17, 19
19	14.1	0.89, t *(7.3)*	18	17, 18	17, 18
3-OH		10.48, s		2, 4	2-CH_3
15-OH					
2-CH_3	9.2	1.82, s		1, 2, 3	
4-CH_3	9.8	1.88, s	6	3, 4, 5	6
8-CH_3	16.3	1.66, s	6, 7	7, 8, 9	6, 9
12-CH_3	12.8	1.65, s	13	12, 13	10, 14
14-CH_3	17.9	0.77, d *(6.8)*	14	13, 14, 15	13, 14, 15
16-CH_3	11.4	1.50, s	17, 18	15, 16, 17	14, 18

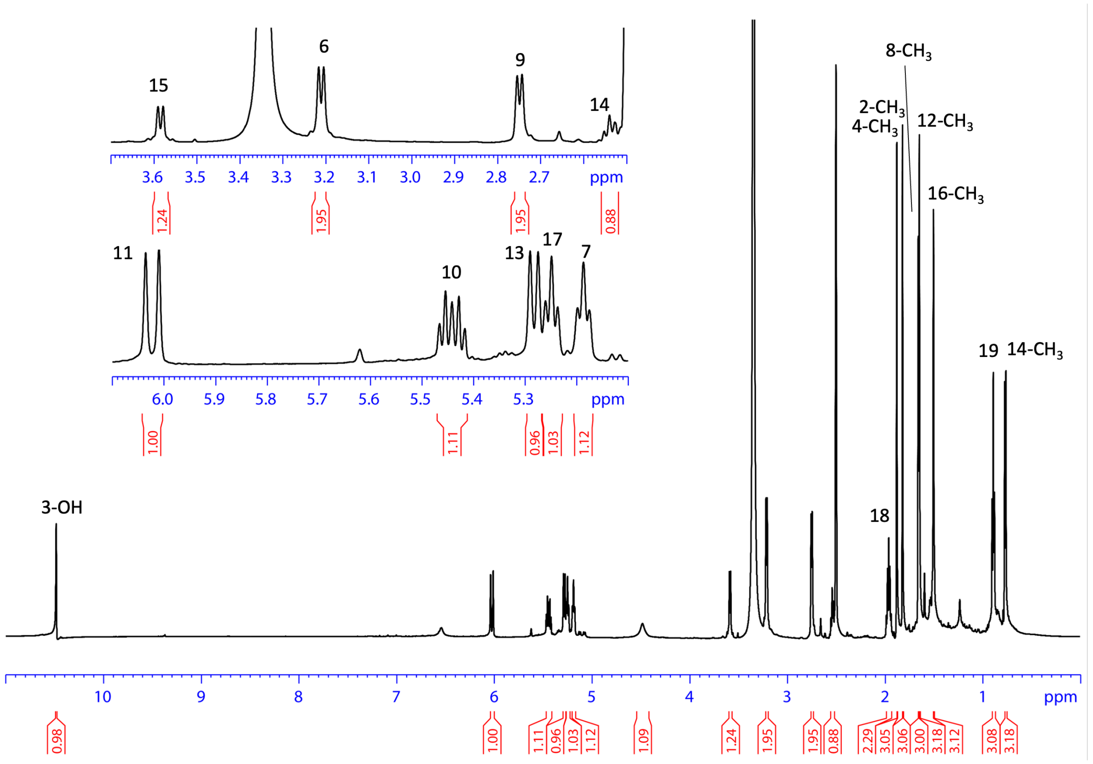

Figure S33. ^{1}H NMR (DMSO-d_6) spectrum of goondapyrone E (**5**).

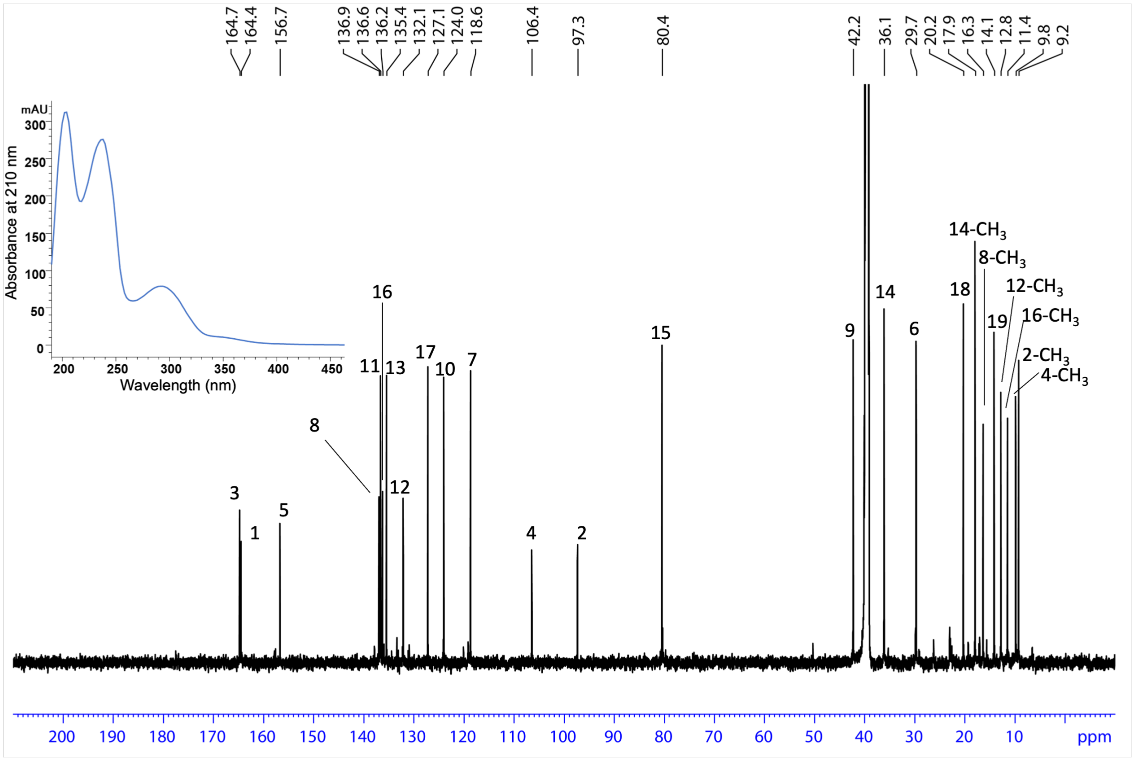

Figure S34. ^{13}C NMR (DMSO-d_6) and UV-vis (inset) spectra of goondapyrone E (**5**).

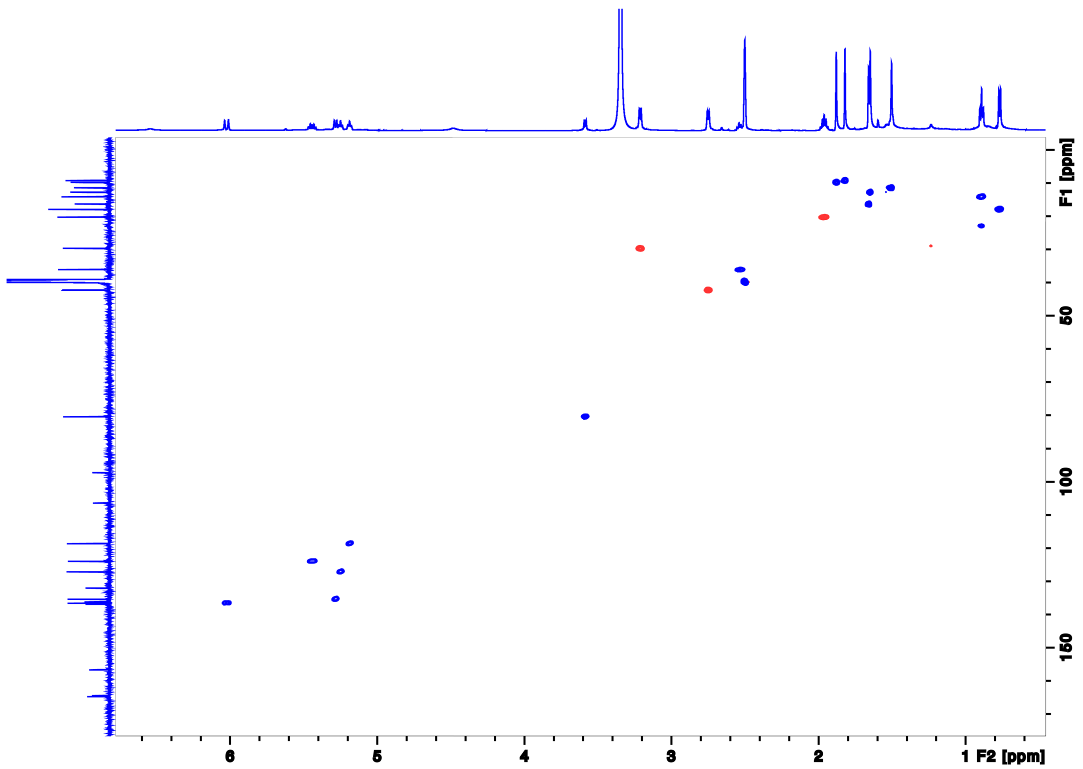

Figure S35. HSQC NMR (DMSO-d_6) spectrum of goondapyrone E (**5**).

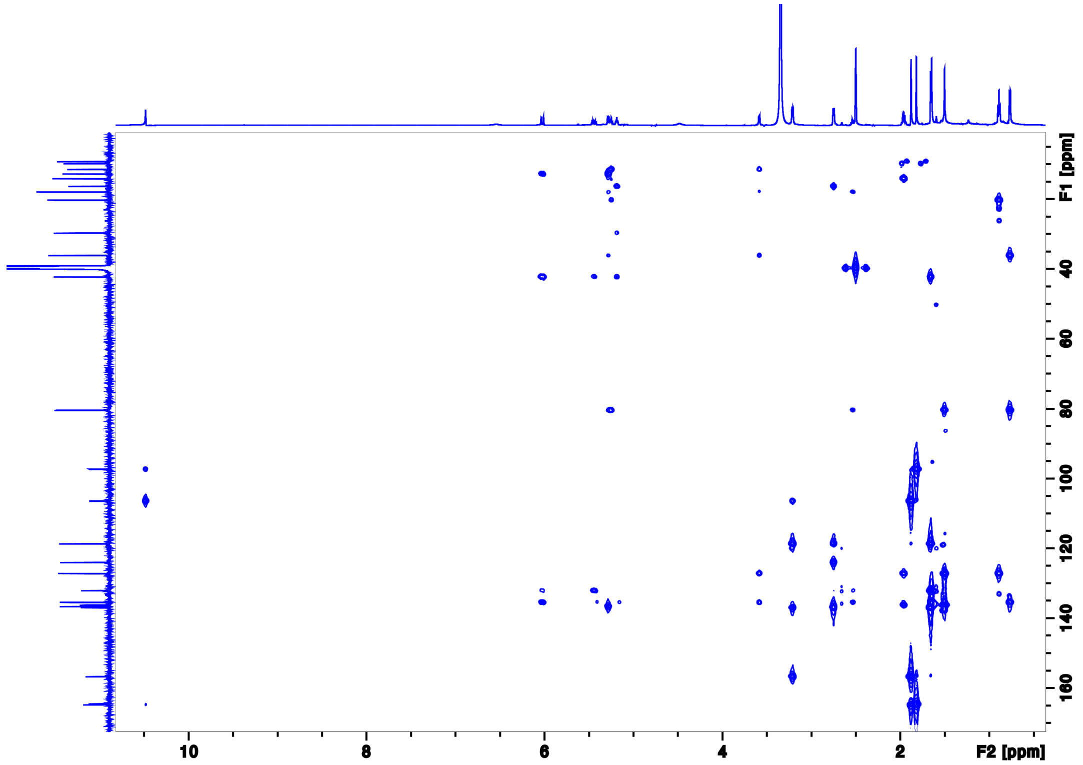

Figure S36. HMBC NMR (DMSO-d_6) spectrum of goondapyrone E (**5**).

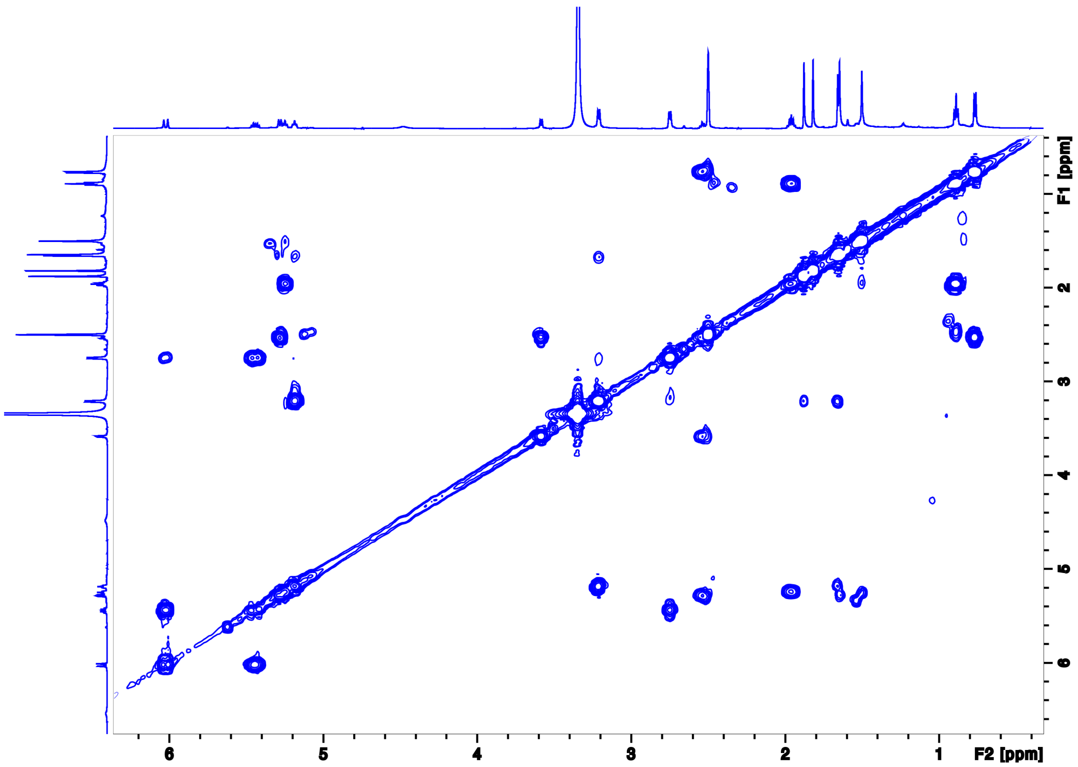

Figure S37. COSY NMR (DMSO-d_6) spectrum of goondapyrone E (**5**).

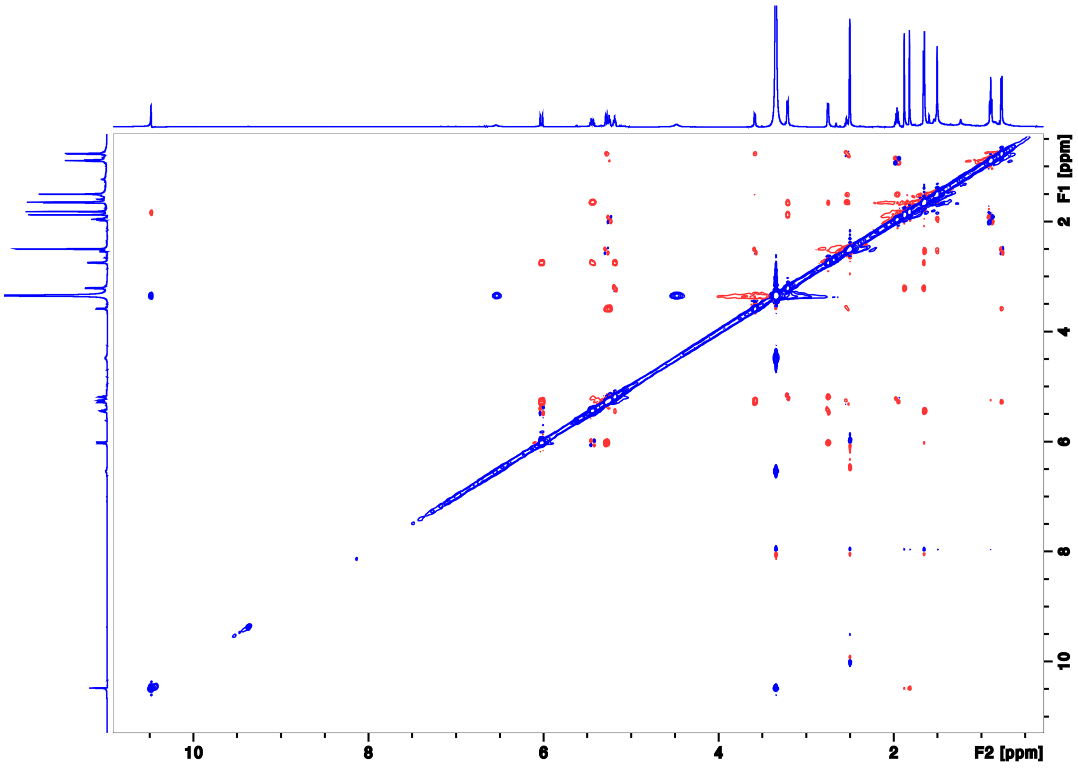

Figure S38. ROESY NMR (DMSO-d_6) spectrum of goondapyrone E (**5**).

Mass Spectrum Molecular Formula Report

Analysis Info

Analysis Name	D:\Data\Shengbin\Shengbin_S4S196A10_F30-32D_3-2.d	Acquisition Date	9/21/2022 3:48:07 PM
Method	tune-medhigh_AP.m	Operator	a.salim
Sample Name	Shengbin_S4S196A10_F30-32D_3-2	Instrument / Ser#	micrOTOF 213750.00232
Comment			

Acquisition Parameter

Source Type	ESI	Ion Polarity	Positive	Set Nebulizer	0.5 Bar
Focus	Not active			Set Dry Heater	180 °C
Scan Begin	100 m/z	Set Capillary	4500 V	Set Dry Gas	5.0 l/min
Scan End	1500 m/z	Set End Plate Offset	-500 V	Set Divert Valve	Source

Generate Molecular Formula Parameter

Formula, min.		
Formula, max.		
Measured m/z	Tolerance	Charge
Check Valence	Minimum	Maximum
Nirogen Rule	Electron Configuration	
Filter H/C Ratio	Minimum	Maximum
Estimate Carbon		

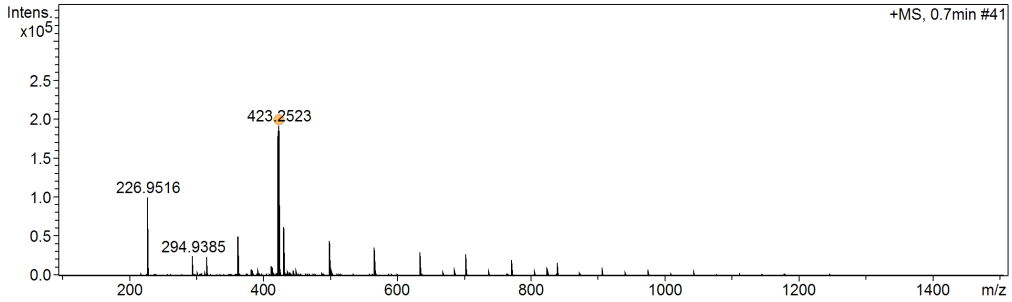

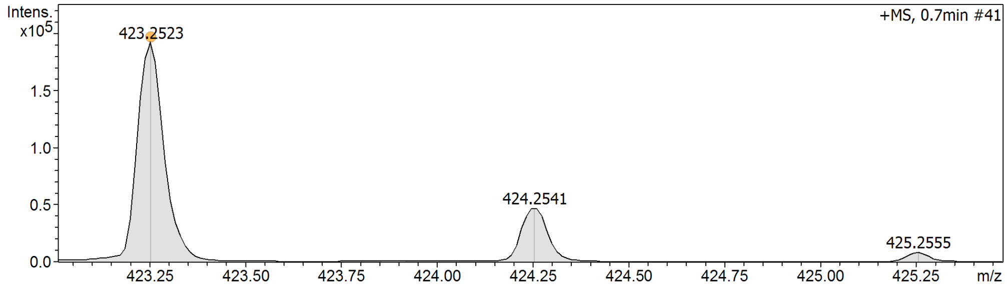

Meas. m/z	#	Ion Formula	m/z	err [ppm]	mSigma	# Sigma	Score	rdb	e⁻ Conf	N-Rule
423.2523	1	C25H36NaO4	423.2506	4.1	15.5	1	59.34	7.5	even	ok

Bruker Compass DataAnalysis 4.1 printed: 10/25/2023 4:28:15 PM Page 1 of 1

Figure S39. HRESIMS spectrum for goondapyrone E (**5**).

2.6 Goondapyrone F (6)

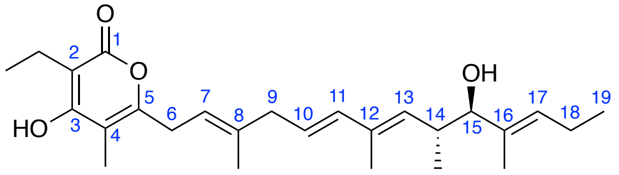

Table S7. 1D and 2D NMR (DMSO-d_6) data for goondapyrone F (**6**).

**Pos.**	δ_C	δ_H, mult (*J* in Hz)	COSY	^{1}H-^{13}C HMBC	ROESY
1	164.0				
2	103.5				
3	164.2				
4	106.4				
5	156.9				
6	29.7	3.21, d *(6.9)*	7, 8-CH_3	4, 5, 7, 8	7, 4-CH_3, 8-CH_3
7	118.6	5.19, t *(6.8)*	6	6, 9, 8-CH_3	6, 9
8	136.9				
9	42.2	2.75, d *(6.9)*	10	7, 8, 10, 8-CH_3	7, 10, 11, 8-CH_3
10	124.0	5.44, dt *(15.5, 7.0)*	9, 11	9, 12	9, 12-CH_3
11	136.6	6.02, d *(15.5)*	9, 10	9, 13, 12-CH_3	9, 13
12	132.1				
13	135.4	5.28, d *(9.3)*	14-CH_3	11, 14, 12-CH_3, 14-CH_3	11, 14, 14-CH_3
14	36.1	2.53, m	13, 15, 14-CH_3	12, 13, 15, 14-CH_3	15, 12-CH_3, 14-CH_3, 16-CH_3
15	80.4	3.58, d *(7.2)*	14	13, 14, 17, 14-CH_3, 16-CH_3	13, 14, 14-CH_3
16	136.2				
17	127.1	5.25, t *(7.2)*	18	15, 18, 16-CH_3	15, 18, 19
18	20.2	1.96, m	17, 19	16, 17, 19	17, 19
19	14.1	0.89, t *(7.5)*	18	17, 18	18
3-OH		10.44, s		2, 4	4-CH_3, 2-$\underline{CH_2}CH_3$
15-OH					
4-CH_3	9.8	1.88, s	6	3, 4, 5	6
8-CH_3	16.3	1.66, s	6, 7	7, 8, 9	6, 9
12-CH_3	12.8	1.65, s	13	12, 13	10, 14
14-CH_3	17.9	0.77, d *(6.8)*	14	13, 14, 15	14
16-CH_3	11.4	1.50, s	17	15, 16, 17	14, 18
2-$\underline{CH_2}CH_3$	16.5	2.35, q *(7.4)*	2-$CH_2\underline{CH_3}$	1, 2, 3, 2-$CH_2\underline{CH_3}$	2-$CH_2\underline{CH_3}$
2-$CH_2\underline{CH_3}$	12.8	0.93, t *(7.4)*	2-$\underline{CH_2}CH_3$	2, 2-$\underline{CH_2}CH_3$	2-$\underline{CH_2}CH_3$

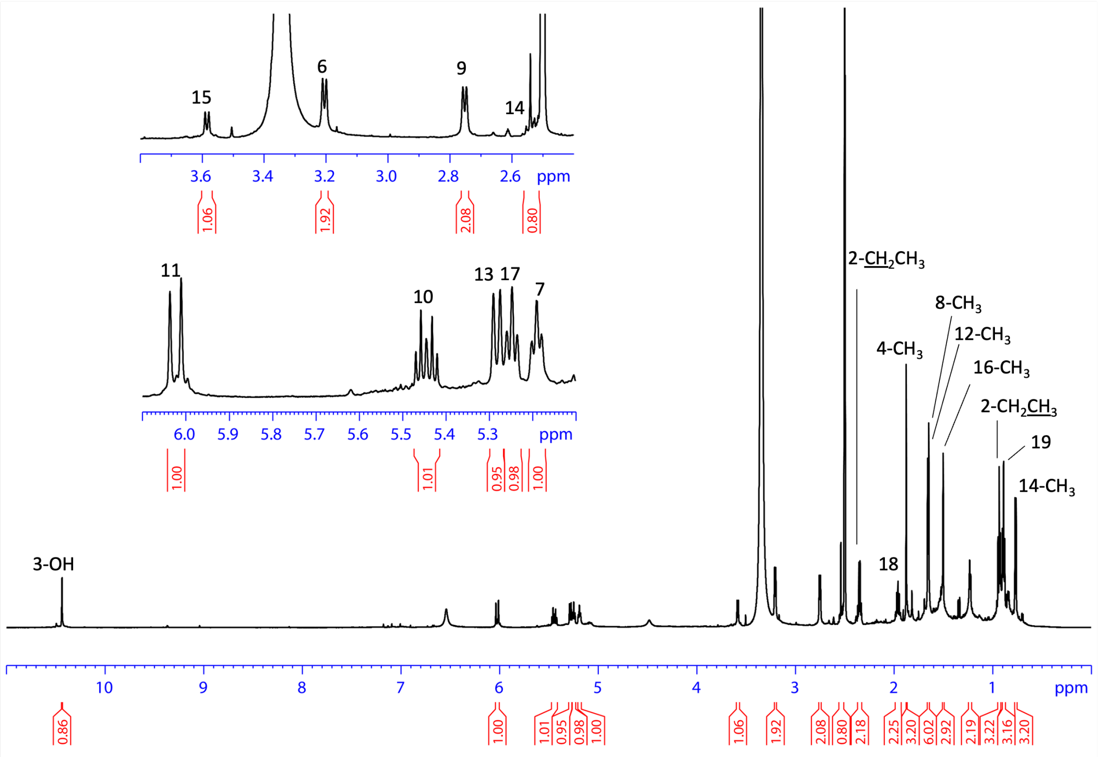

Figure S40. ^{1}H NMR (DMSO-d_6) spectrum of goondapyrone F (**6**).

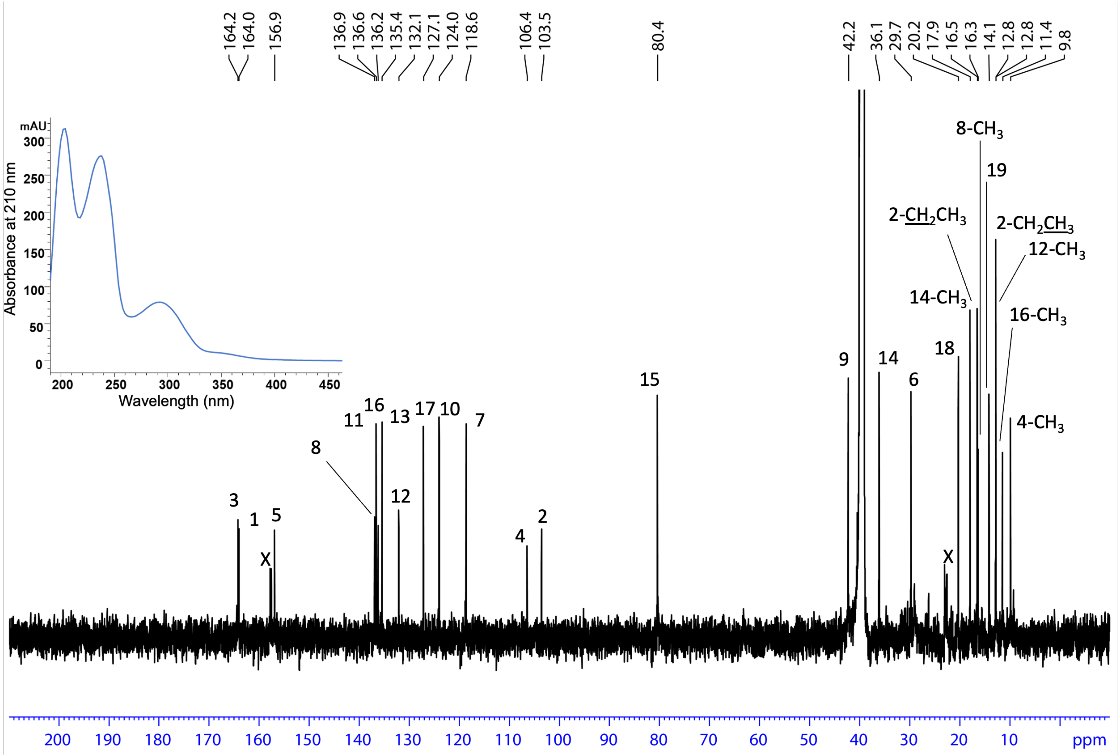

Figure S41. ^{13}C NMR (DMSO-d_6) and UV-vis (inset) spectra of goondapyrone F (**6**).

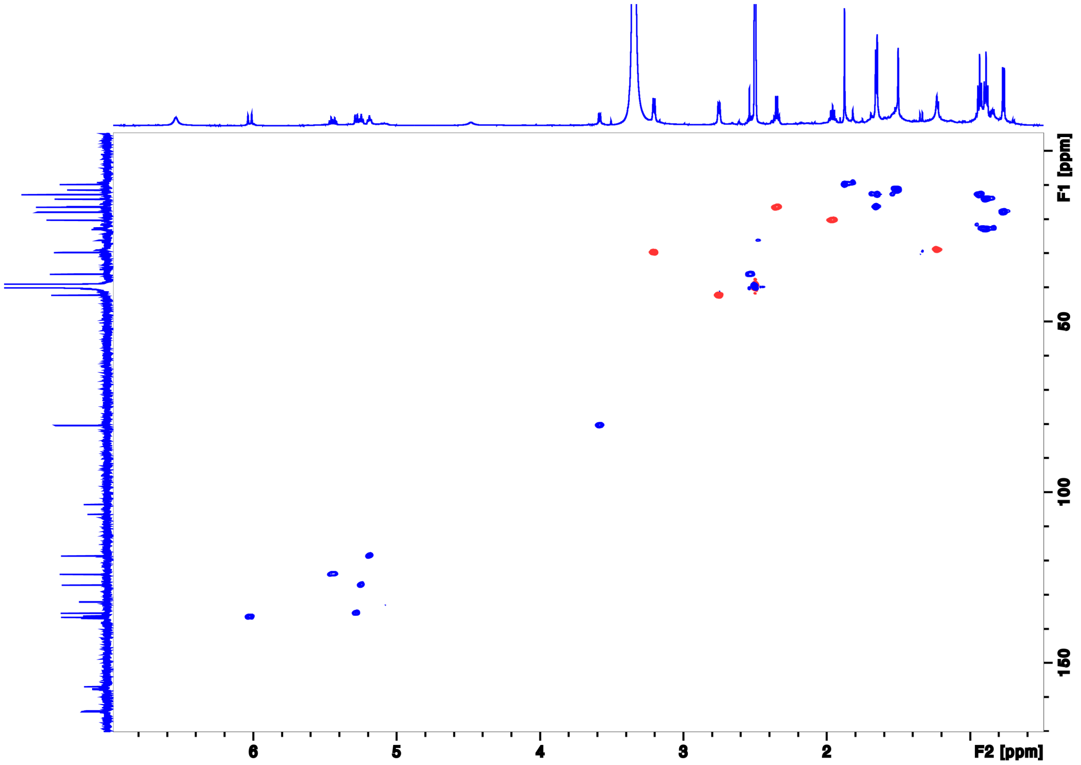

Figure S42. HSQC NMR (DMSO-d_6) spectrum of goondapyrone F (**6**).

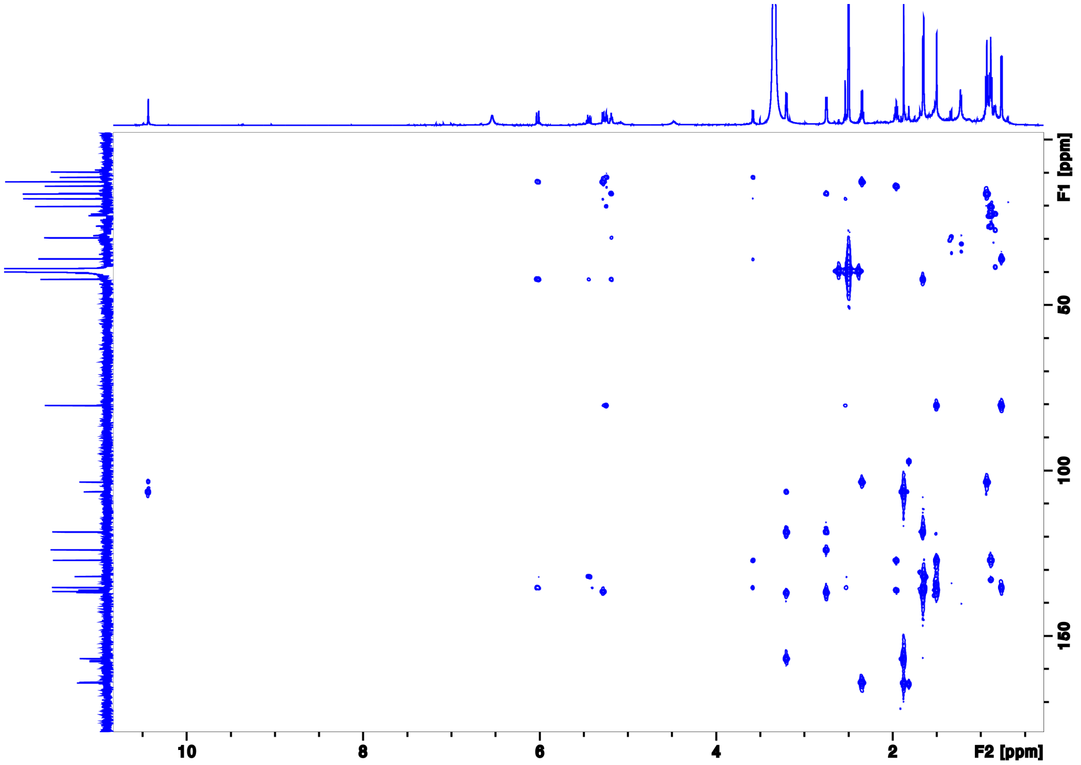

Figure S43. HMBC NMR (DMSO-d_6) spectrum of goondapyrone F (**6**).

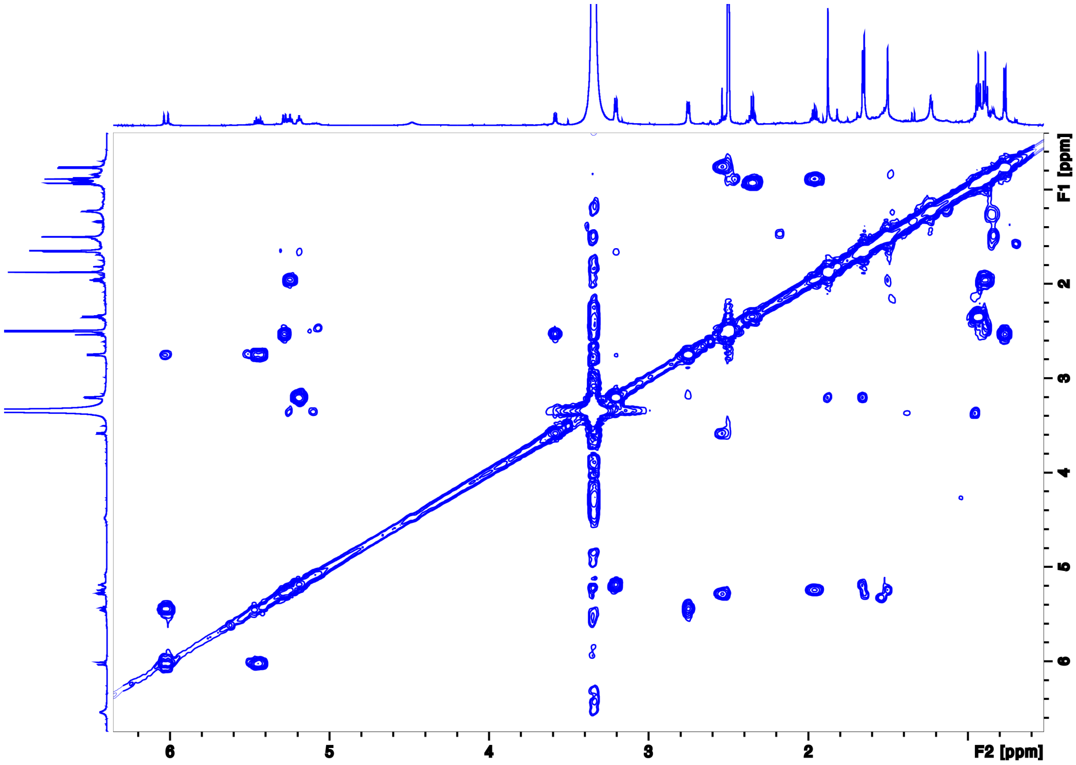

Figure S44. COSY NMR (DMSO-d_6) spectrum of goondapyrone F (**6**).

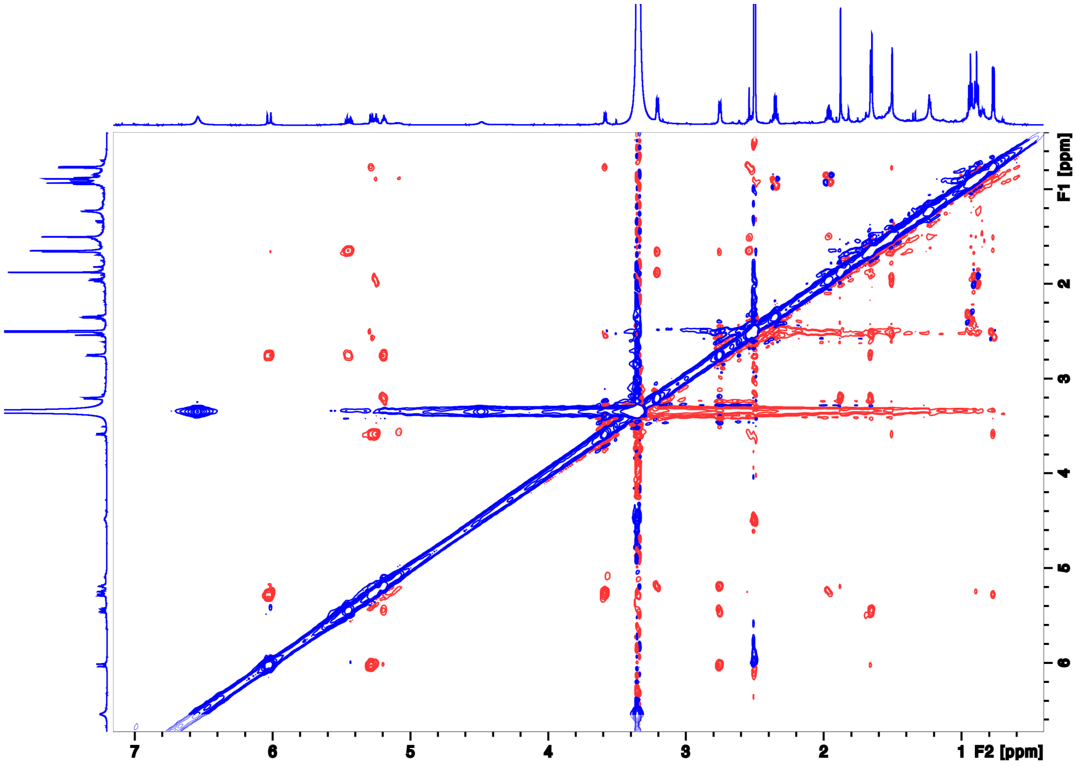

Figure S45. ROESY NMR (DMSO-d_6) spectrum of goondapyrone F (**6**).

Mass Spectrum Molecular Formula Report

Analysis Info

Analysis Name	D:\Data\Shengbin\Shengbin_S4S196A10_F30-32D_5-2.d	Acquisition Date	9/21/2022 4:00:55 PM
Method	tune-medhigh_AP.m	Operator	a.salim
Sample Name	Shengbin_S4S196A10_F30-32D_5-2	Instrument / Ser#	micrOTOF 213750.00232
Comment			

Acquisition Parameter

Source Type	ESI	Ion Polarity	Positive	Set Nebulizer	0.5 Bar
Focus	Not active			Set Dry Heater	180 °C
Scan Begin	100 m/z	Set Capillary	4500 V	Set Dry Gas	5.0 l/min
Scan End	1500 m/z	Set End Plate Offset	-500 V	Set Divert Valve	Source

Generate Molecular Formula Parameter

Formula, min.		
Formula, max.		
Measured m/z	Tolerance	Charge
Check Valence	Minimum	Maximum
Nirogen Rule	Electron Configuration	
Filter H/C Ratio	Minimum	Maximum
Estimate Carbon		

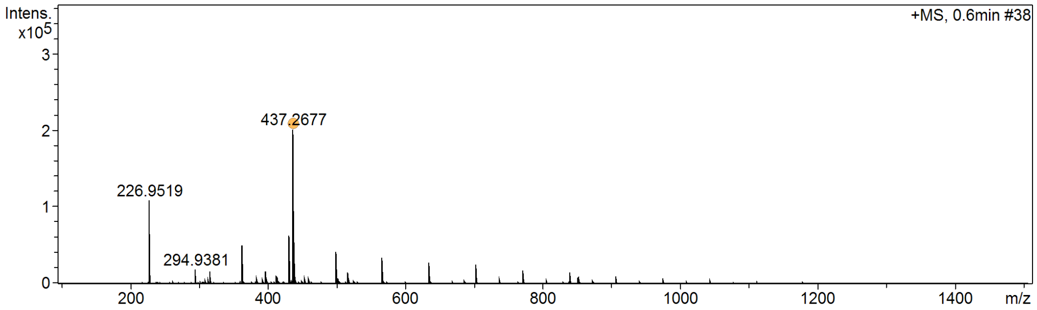

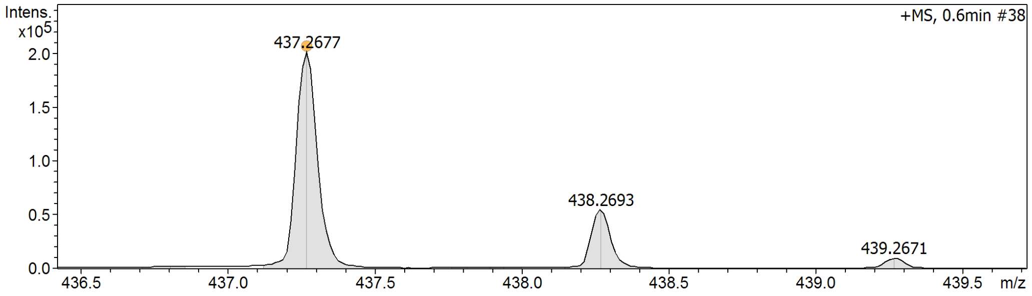

Meas. m/z	#	Ion Formula	m/z	err [ppm]	mSigma	# Sigma	Score	rdb	e⁻ Conf	N-Rule
437.2677	1	C26H38NaO4	437.2662	-3.4	7.9	1	60.97	7.5	even	ok

Bruker Compass DataAnalysis 4.1 printed: 3/27/2023 10:57:03 AM Page 1 of 1

Figure S46. HRESIMS spectrum for goondapyrone F (**6**).

2.7 Goondapyrone G (7)

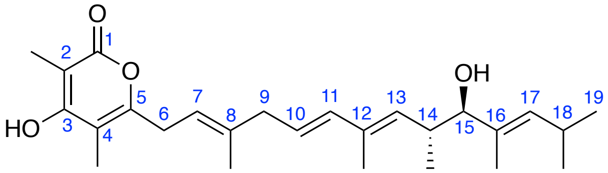

Table S8. 1D and 2D NMR (600 MHz, DMSO-d_6) data for goondapyrone G (**7**).

**Pos.**	δ_C	δ_H, mult (J in Hz)	COSY	^{1}H-^{13}C HMBC	ROESY
1	164.6				
2	97.1				
3	165.2				
4	106.6				
5	156.6				
6	29.7	3.20, d *(7.0)*	7	4, 5, 7, 8	7, 4-CH_3, 8-CH_3
7	118.7	5.19, t *(7.0)*	6, 8-CH_3	6, 9, 8-CH_3	6, 9
8	136.9				
9	42.3	2.75, d *(6.9)*	10	8, 10, 11, 8-CH_3	7, 10, 11, 8-CH_3
10	124.0	5.44, dt *(15.6, 6.9)*	9, 11	8, 9, 12	9, 11, 12-CH_3
11	136.6	6.03, d *(15.6)*	9, 10	9, 12, 13, 12-CH_3	9, 10, 13, 12-CH_3
12	132.1				
13	135.4	5.28, d *(9.4)*	14, 12-CH_3	11, 14, 15, 12-CH_3, 14-CH_3	14, 15, 14-CH_3
14	36.2	2.54^A, m	13, 15, 14-CH_3	12, 13, 15, 14-CH_3	15, 12-CH_3, 14-CH_3, 16-CH_3
15	80.3	3.57, br d *(7.1)*	14, 15-OH	13, 14, 16, 14-CH_3, 16-CH_3	13, 14, 17, 14-CH_3, 16-CH_3
16	134.4				
17	133.1	5.08, d *(9.1)*	18, 16-CH_3	15, 18, 19, 16-CH_3, 18-CH_3	15, 18, 19, 18-CH_3
18	26.2	2.48^A, m	17, 19, 18-CH_3	17, 19, 18-CH_3	19, 16-CH_3, 18-CH_3
19	23.0	0.89, d *(5.3)*	18	17, 18, 18-CH_3	17, 18, 18-CH_3
3-OH					
15-OH		4.48, br s	15		
2-CH_3	9.3	1.81, s		1, 2, 3	
4-CH_3	9.9	1.87, s	6	3, 4, 5	6
8-CH_3	16.4	1.66, s	6, 7	7, 8, 9	6, 9
12-CH_3	12.8	1.65, s	13	12, 13	10, 14
14-CH_3	17.9	0.78, d *(6.8)*	14	13, 14, 15	13, 14, 15
16-CH_3	11.6	1.52, s	17	15, 16, 17	18
18-CH_3	22.9	0.88, d *(5.3)*	18	17, 18, 19	17, 18, 19

^{A} overlapping signals

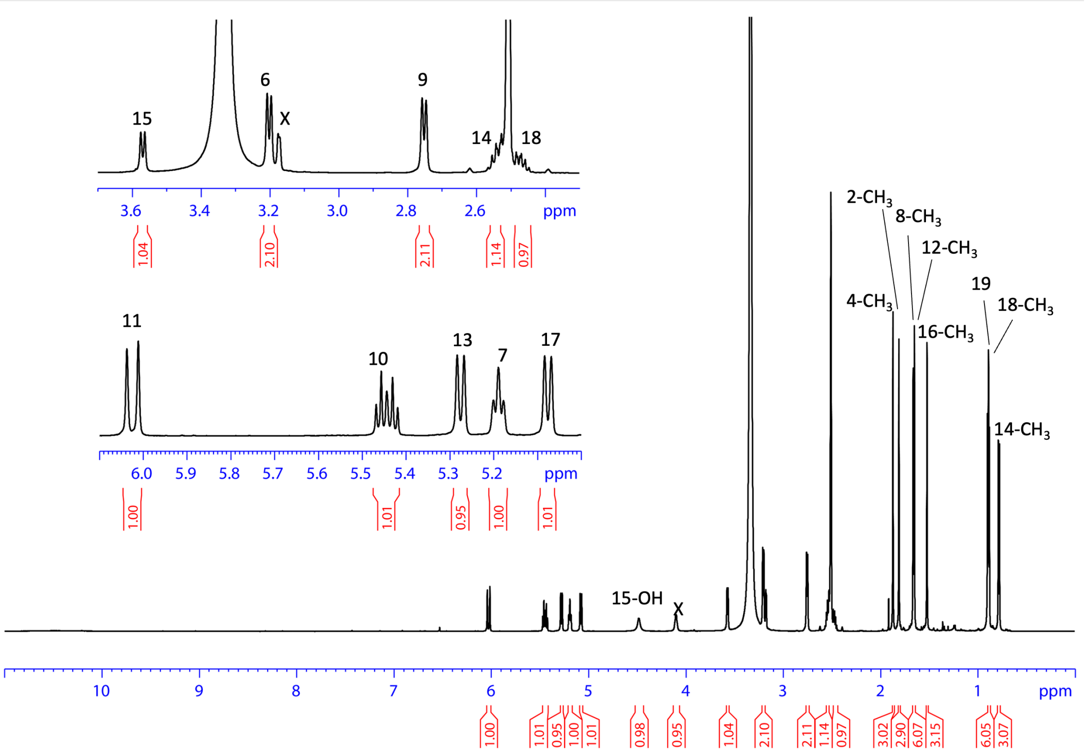

Figure S47. ^{1}H NMR (DMSO-d_6) spectrum of goondapyrone G (**7**).

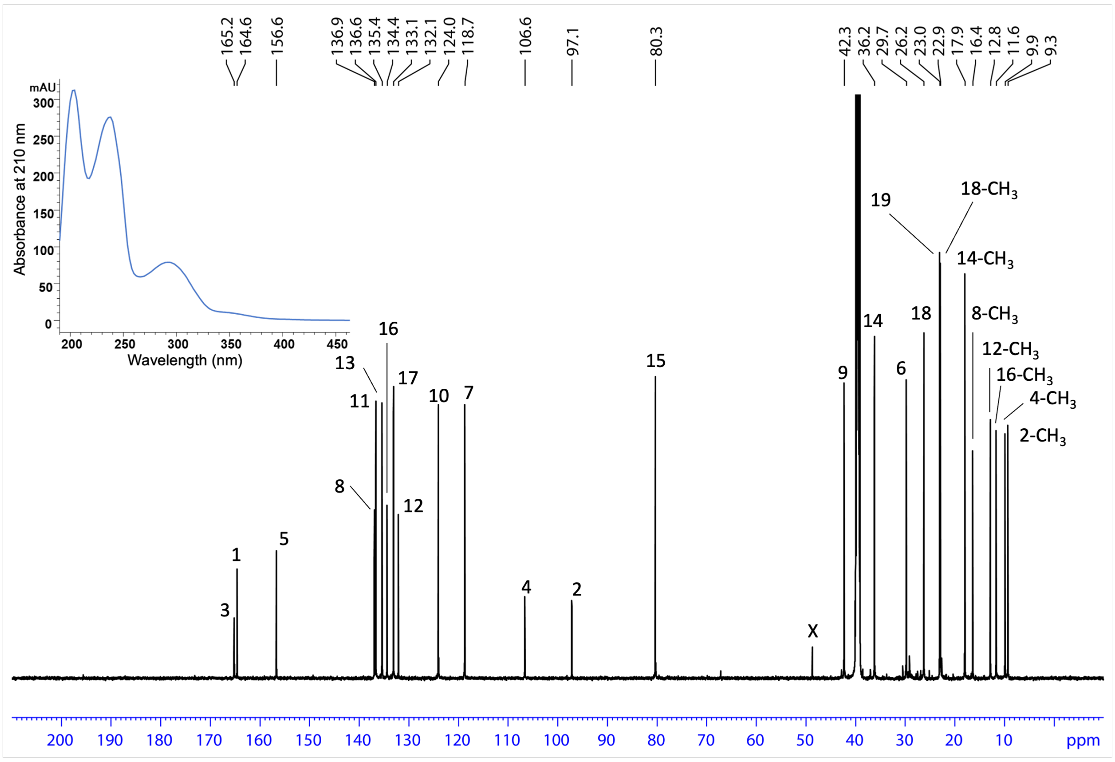

Figure S48. ^{13}C NMR (DMSO-d_6) and UV-vis (inset) spectra of goondapyrone G (**7**).

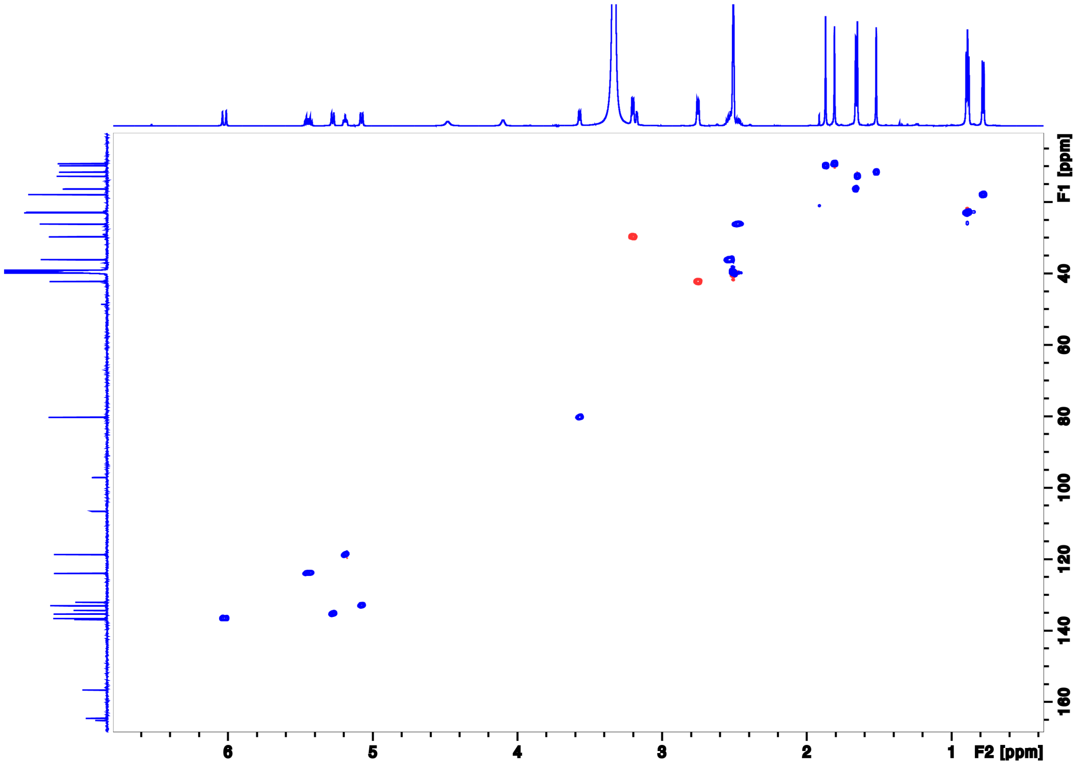

Figure S49. HSQC NMR (DMSO-d_6) spectrum of goondapyrone G (**7**).

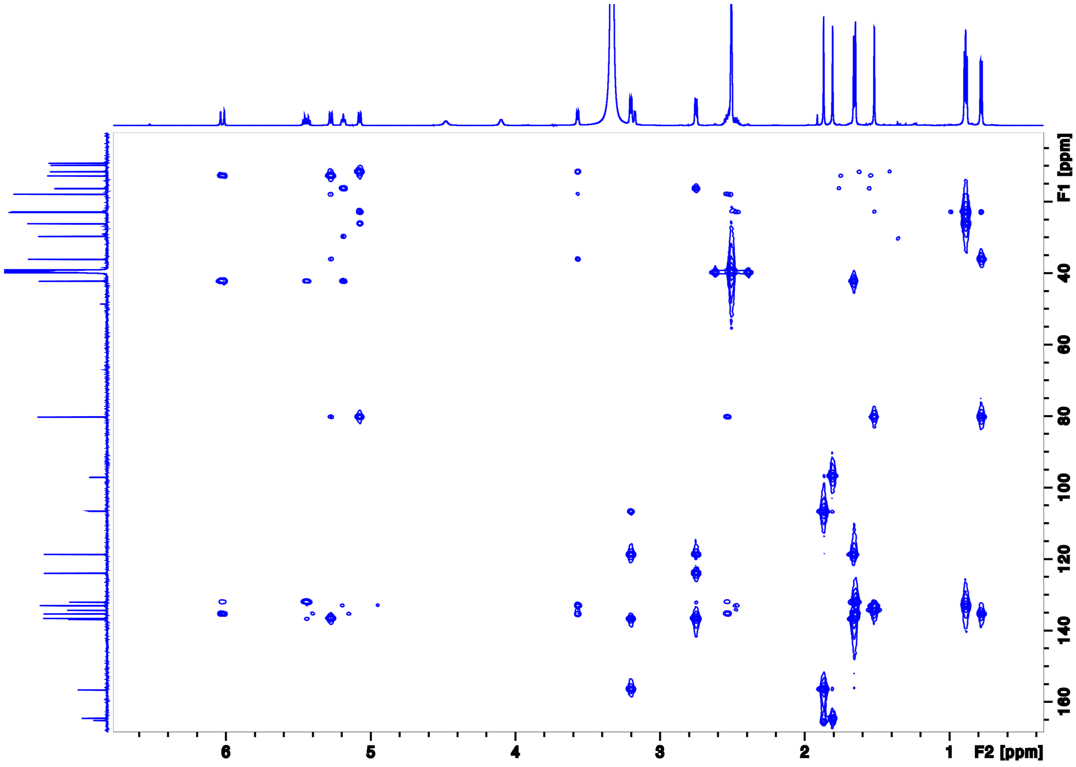

Figure S50. HMBC NMR (DMSO-d_6) spectrum of goondapyrone G (**7**).

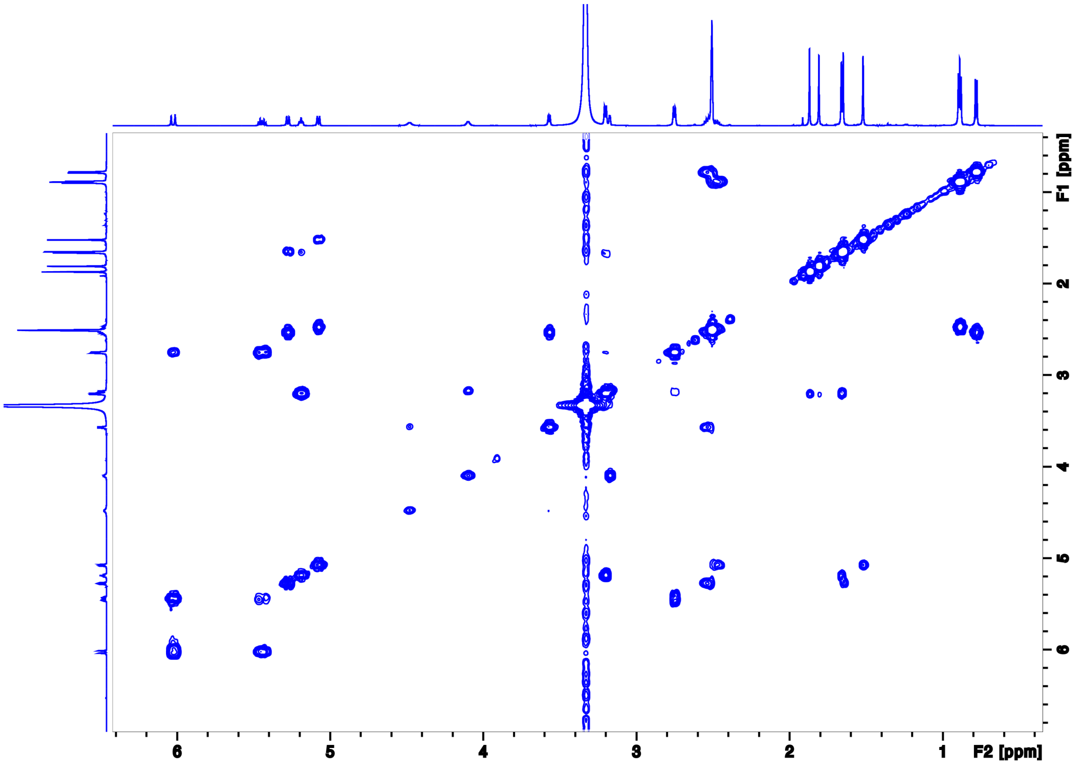

Figure S51. COSY NMR (DMSO-d_6) spectrum of goondapyrone G (**7**).

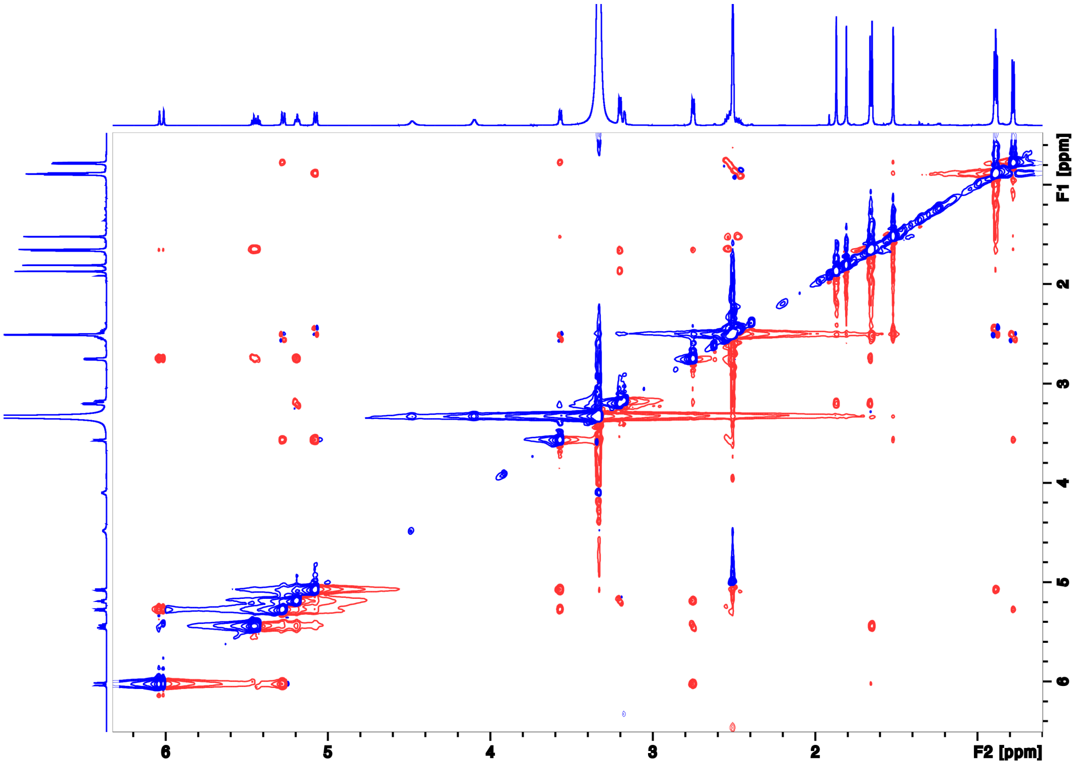

Figure S52. ROESY NMR (DMSO-d_6) spectrum of goondapyrone G (**7**).

Mass Spectrum Molecular Formula Report

Analysis Info

Analysis Name	D:\Data\Shengbin\Shengbin_S4S196A10_F30-32D_4-1.d	Acquisition Date	9/21/2022 3:54:46 PM
Method	tune-medhigh_AP.m	Operator	a.salim
Sample Name	Shengbin_S4S196A10_F30-32D_4-1	Instrument / Ser#	micrOTOF 213750.00232
Comment			

Acquisition Parameter

Source Type	ESI	Ion Polarity	Positive	Set Nebulizer	0.5 Bar
Focus	Not active			Set Dry Heater	180 °C
Scan Begin	100 m/z	Set Capillary	4500 V	Set Dry Gas	5.0 l/min
Scan End	1500 m/z	Set End Plate Offset	-500 V	Set Divert Valve	Source

Generate Molecular Formula Parameter

Formula, min.		
Formula, max.		
Measured m/z	Tolerance	Charge
Check Valence	Minimum	Maximum
Nirogen Rule	Electron Configuration	
Filter H/C Ratio	Minimum	Maximum
Estimate Carbon		

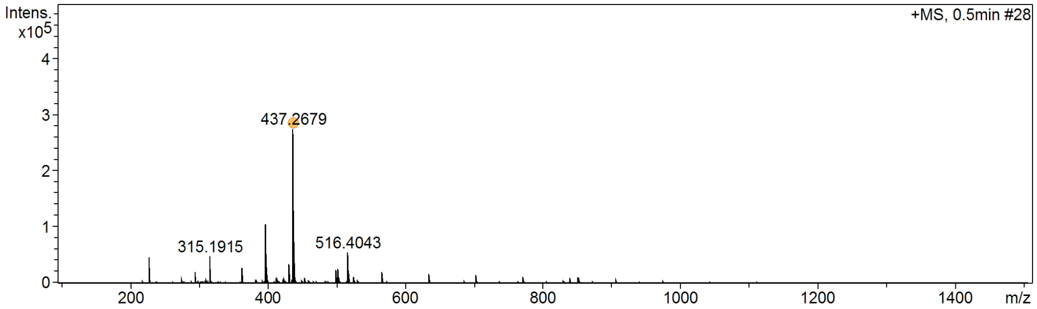

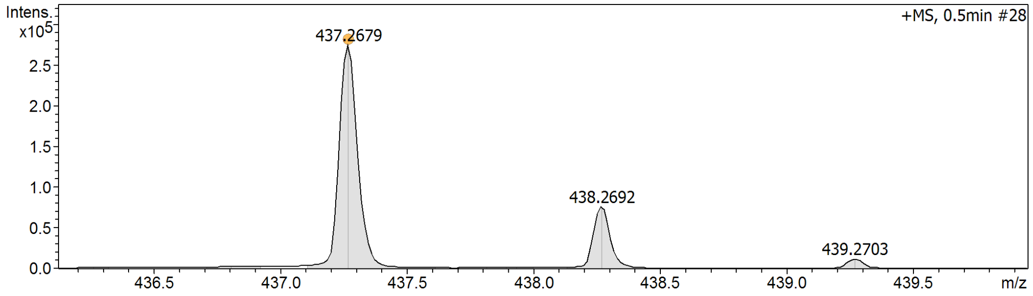

Meas. m/z	#	Ion Formula	m/z	err [ppm]	mSigma	# Sigma	Score	rdb	e⁻ Conf	N-Rule
437.2679	1	C26H38NaO4	437.2662	3.8	6.9	1	57.98	7.5	even	ok

Bruker Compass DataAnalysis 4.1 printed: 3/27/2023 10:53:28 AM Page 1 of 1

Figure S53. HRESIMS spectrum for goondapyrone G (**7**).

2.8 Goondapyrone H (8)

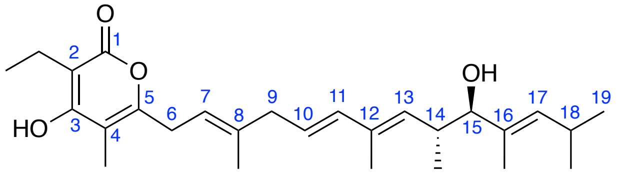

Table S9. 1D and 2D NMR (600 MHz, DMSO-d_6) data for goondapyrone H (**8**).

**Pos.**	δ_C	δ_H, mult (*J* in Hz)	COSY	^{1}H-^{13}C HMBC	ROESY
1	164.0				
2	103.5				
3	164.2				
4	106.4				
5	156.9				
6	29.7	3.20, d *(7.0)*	7, 8-CH_3	4, 5, 7, 8	7, 4-CH_3, 8-CH_3
7	118.6	5.19, t *(7.0)*	6	6, 9, 8-CH_3	6, 9
8	136.9				
9	42.2	2.75, d *(6.9)*	10	7, 8, 10, 8-CH_3	7, 10, 11, 8-CH_3
10	124.0	5.44, dt *(15.6, 6.9)*	9, 11	8, 9, 12	9, 12-CH_3
11	136.6	6.02, d *(15.6)*	9, 10	9, 12, 13, 12-CH_3	9, 13, 12-CH_3
12	132.0				
13	135.3	5.27, d *(9.3)*	14	11, 14, 15, 12-CH_3, 14-CH_3	11, 14, 15, 14-CH_3
14	36.1	2.53^A, m	13, 15, 14-CH_3	12, 13, 15, 14-CH_3	15, 12-CH_3, 14-CH_3, 16-CH_3
15	80.2	3.56, dd *(7.1, 4.3)*	14, 15-OH		13, 14, 17, 14-CH_3, 15-OH
16	134.4				
17	133.0	5.07, t *(9.0)*	18, 16-CH_3	18, 19, 16-CH_3, 18-CH_3	15, 18, 19, 18-CH_3
18	26.1	2.47^A, m	17, 19, 18-CH_3	17, 19, 18-CH_3	17, 19, 16-CH_3, 18-CH_3
19	23.0	0.89, d *(5.5)*	18	17, 18, 18-CH_3	17, 18
3-OH					
15-OH		4.48, d *(4.3)*	15	14	15
4-CH_3	9.8	1.88, s	6	3, 4, 5	6
8-CH_3	16.3	1.66, s	6, 7	7, 8, 9	6, 9
12-CH_3	12.8	1.64, s	13	12, 13	10, 14
14-CH_3	17.9	0.77, d *(6.9)*	14	13, 14, 15	13, 14, 15
16-CH_3	11.6	1.51, s	17	15, 16, 17	14, 18
18-CH_3	22.8	0.88, d *(5.5)*	18	17, 18, 19	17, 18
2-$\underline{CH_2}CH_3$	16.5	2.35, q *(7.4)*	2-$CH_2\underline{CH_3}$	1, 2, 3, 2-$CH_2\underline{C}H_3$	2-$CH_2C\underline{H_3}$
2-$CH_2\underline{CH_3}$	12.8	0.93, t *(7.4)*	2-$C\underline{H_2}CH_3$	2, 2-$\underline{C}H_2CH_3$	2-$C\underline{H_2}CH_3$

^{A} overlapping signals

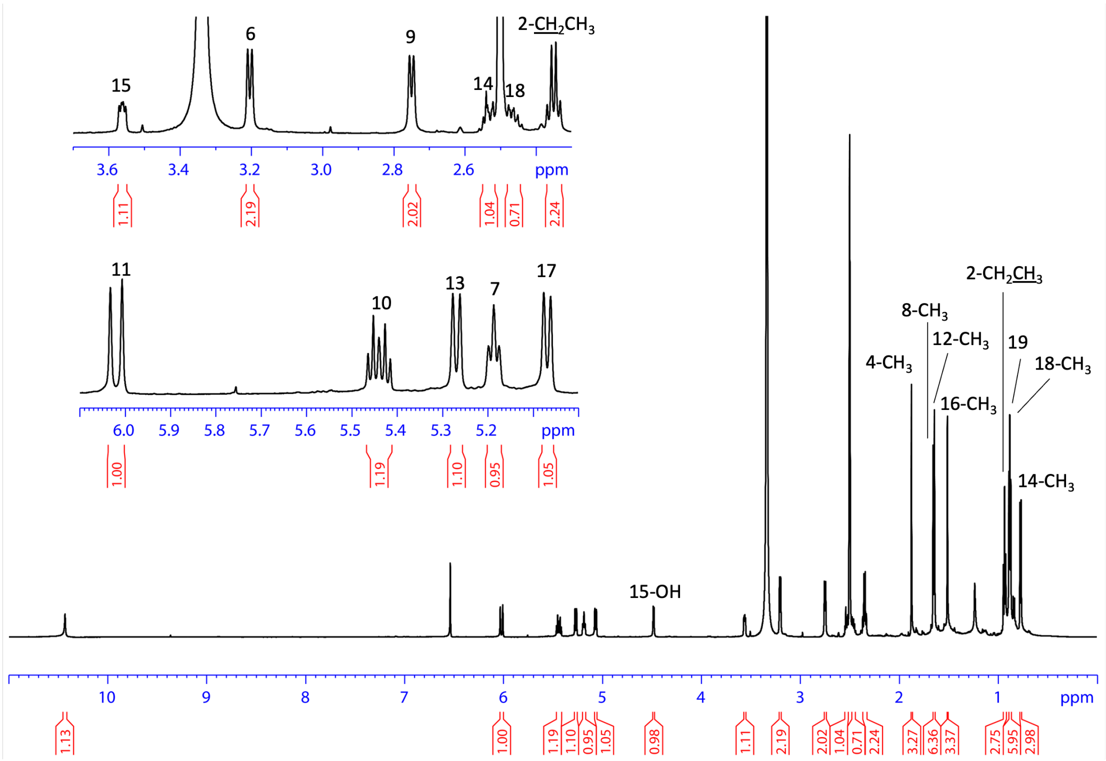

Figure S54. ^{1}H NMR (DMSO-d_6) spectrum of goondapyrone H (**8**).

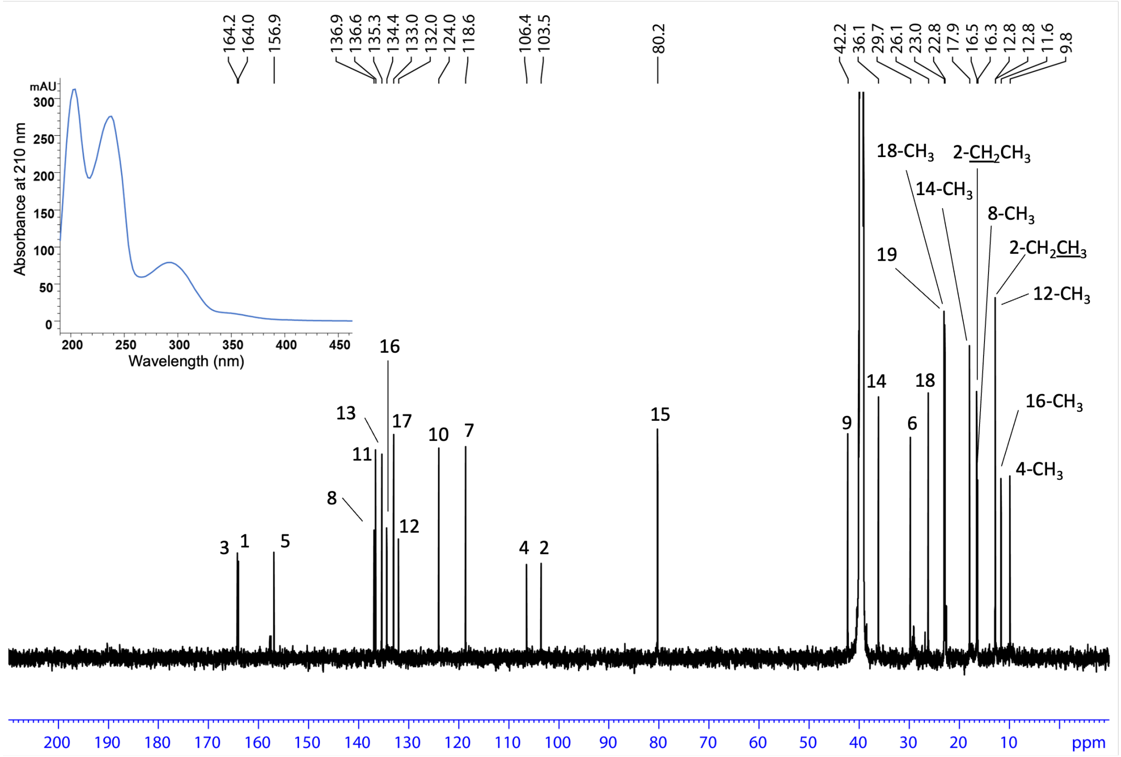

Figure S55. ^{13}C NMR (DMSO-d_6) and UV-vis (inset) spectra of goondapyrone H (**8**).

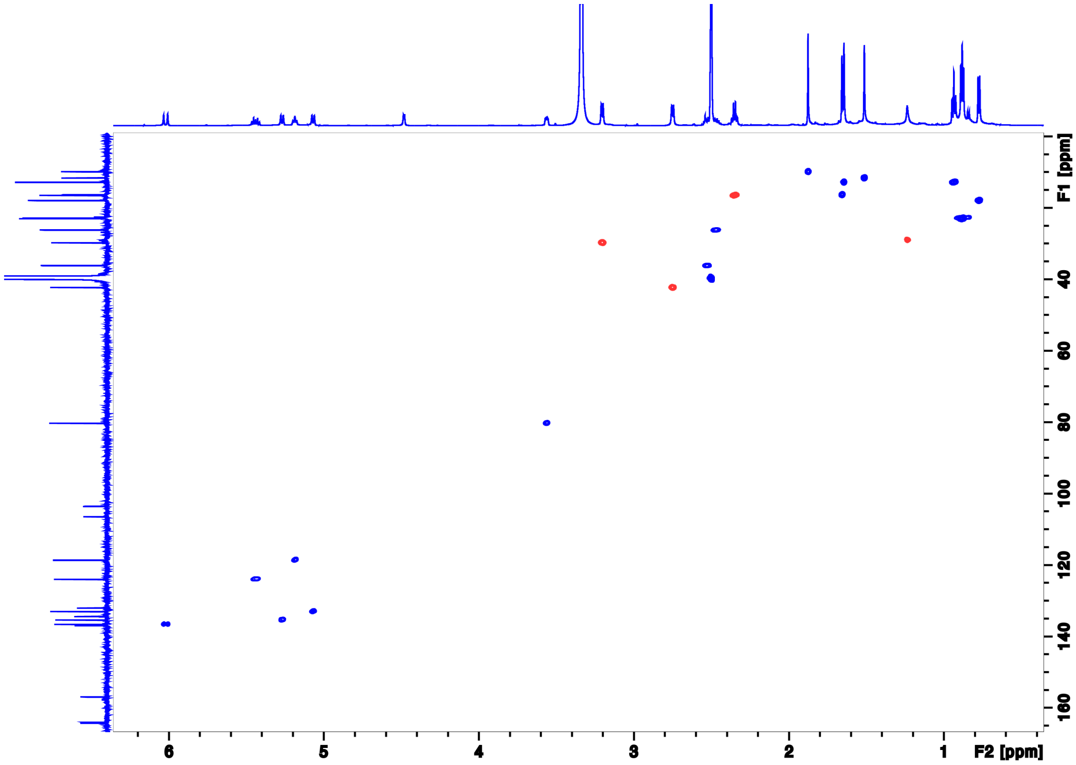

Figure S56. HSQC NMR (DMSO-d_6) spectrum of goondapyrone H (**8**).

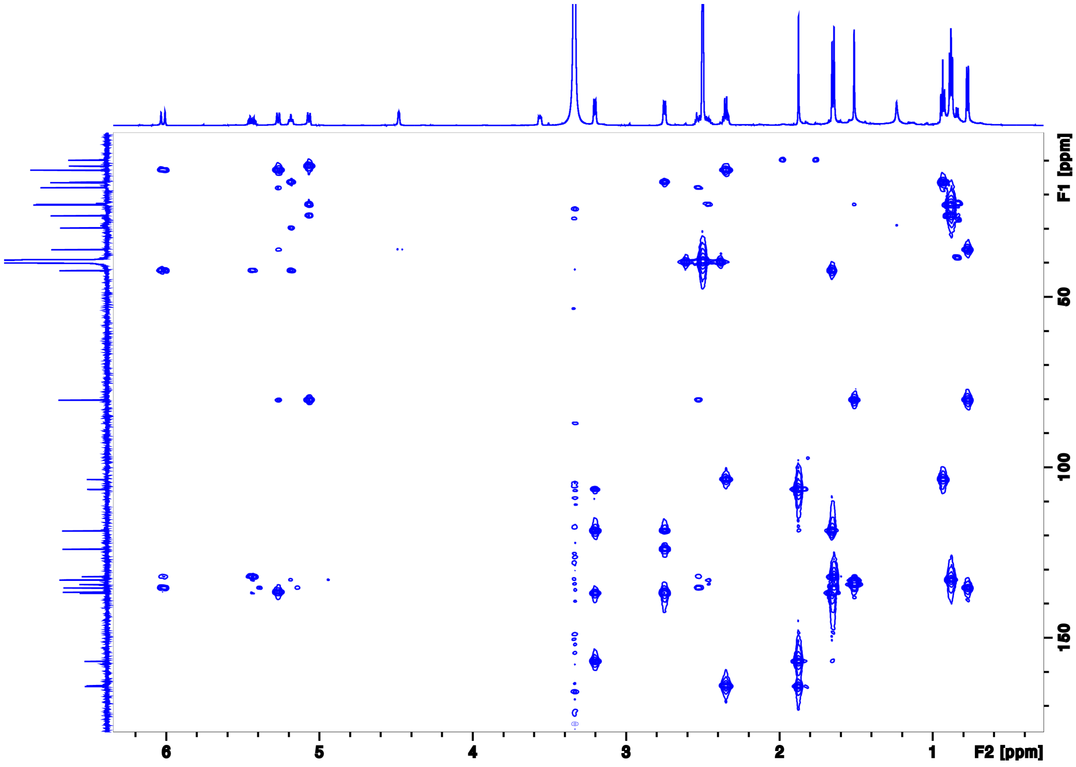

Figure S57. HMBC NMR (DMSO-d_6) spectrum of goondapyrone H (**8**).

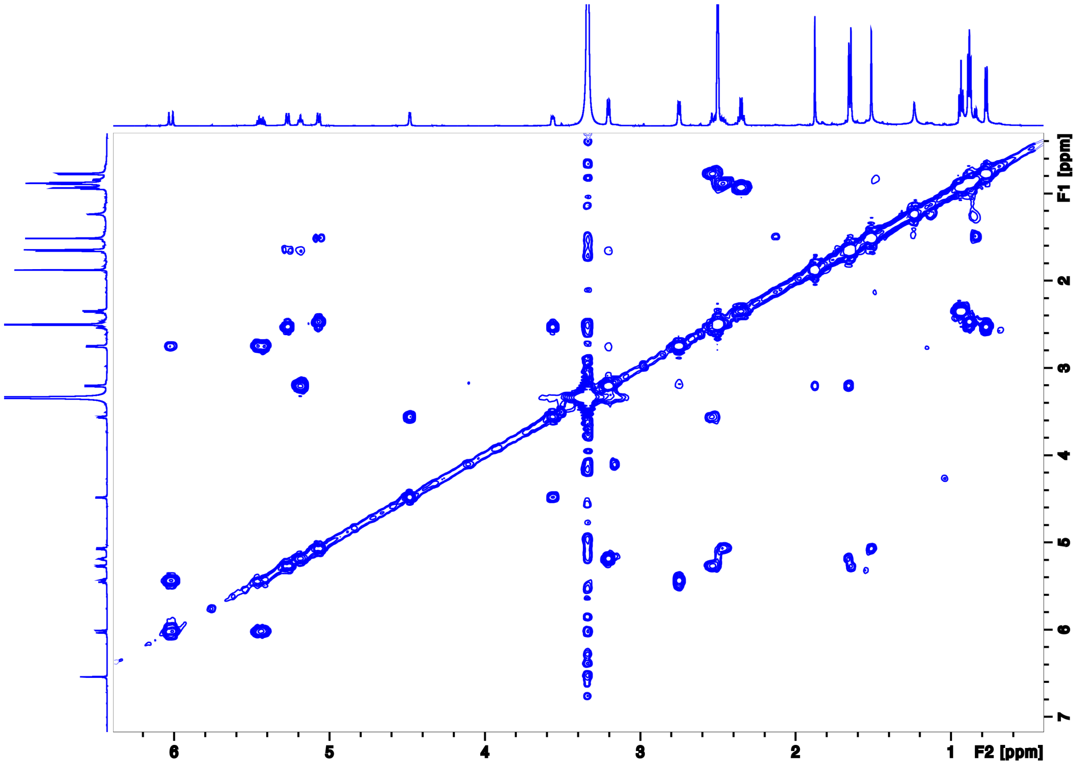

Figure S58. COSY NMR (DMSO-d_6) spectrum of goondapyrone H (**8**).

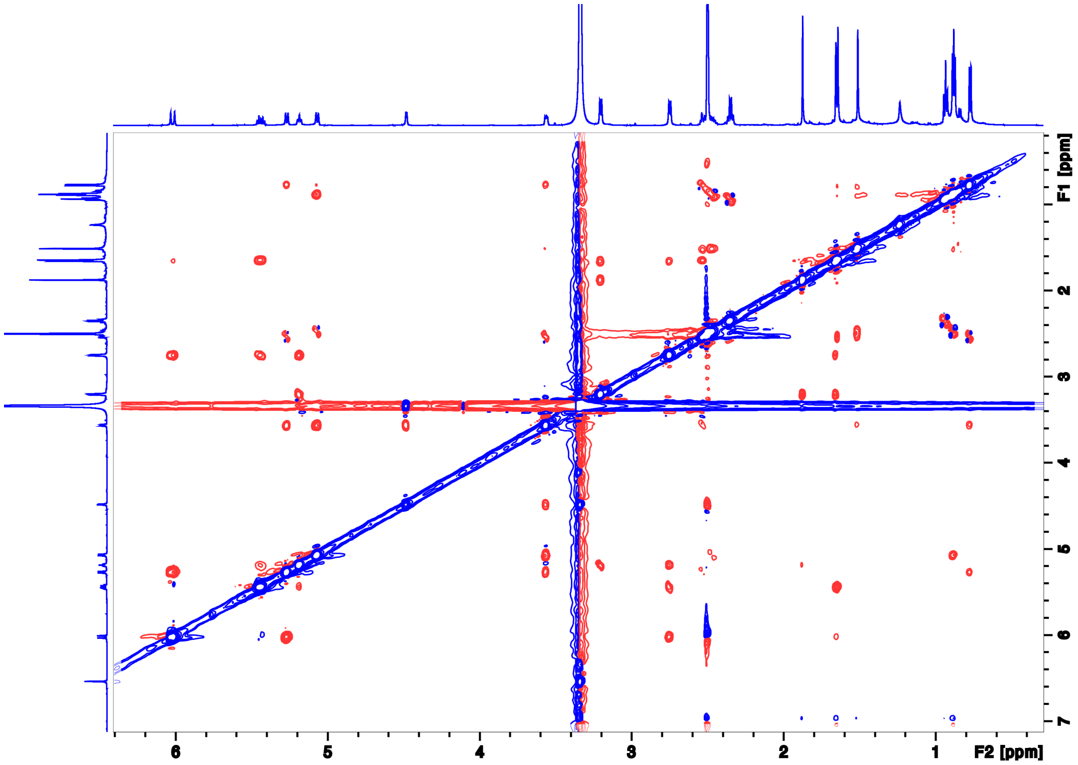

Figure S59. ROESY NMR (DMSO-d_6) spectrum of goondapyrone H (**8**).

Mass Spectrum Molecular Formula Report

Analysis Info

Analysis Name	D:\Data\Shengbin\Shengbin_S4S196A10_F30-32D_6.d	Acquisition Date	9/7/2022 5:20:26 PM
Method	tune-medhigh_AP.m	Operator	a.salim
Sample Name	Shengbin_S4S196A10_F30-32D_6	Instrument / Ser#	micrOTOF 213750.00232
Comment			

Acquisition Parameter

Source Type	ESI	Ion Polarity	Positive	Set Nebulizer	0.5 Bar
Focus	Not active			Set Dry Heater	180 °C
Scan Begin	100 m/z	Set Capillary	4500 V	Set Dry Gas	5.0 l/min
Scan End	1500 m/z	Set End Plate Offset	-500 V	Set Divert Valve	Source

Generate Molecular Formula Parameter

Formula, min.		
Formula, max.		
Measured m/z	Tolerance	Charge
Check Valence	Minimum	Maximum
Nirogen Rule	Electron Configuration	
Filter H/C Ratio	Minimum	Maximum
Estimate Carbon		

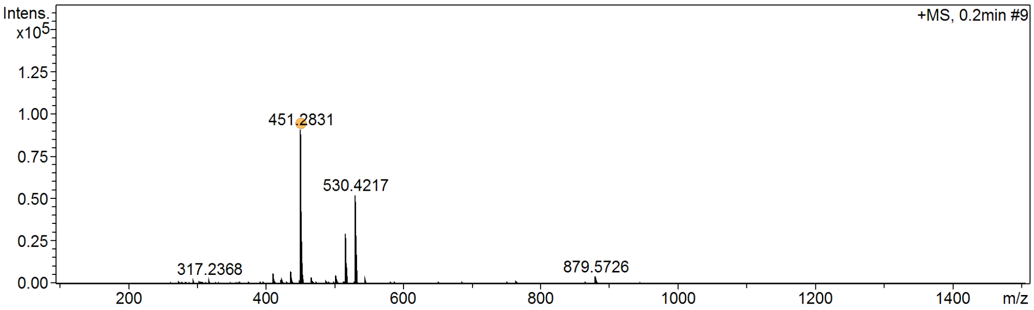

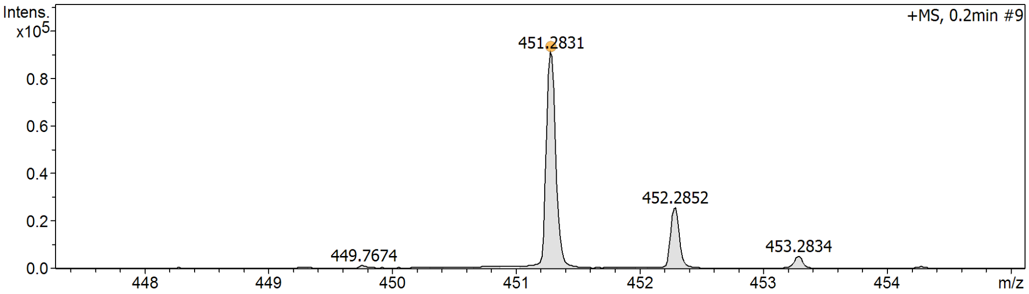

Meas. m/z	#	Ion Formula	m/z	err [ppm]	mSigma	# Sigma	Score	rdb	e⁻ Conf	N-Rule
451.2831	1	C27H40NaO4	451.2819	2.6	8.5	1	73.33	7.5	even	ok

Bruker Compass DataAnalysis 4.1 printed: 3/27/2023 10:59:47 AM Page 1 of 1

Figure S60. HRESIMS spectrum for goondapyrone H (**8**).

2.9 Goondapyrone I (9)

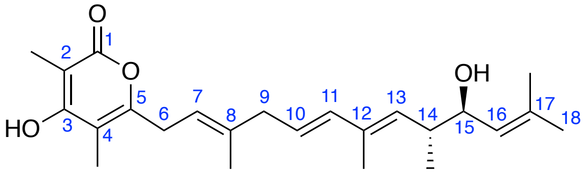

Table S10. 1D and 2D NMR (DMSO-d_6) data for goondapyrone I (**9**).

**Pos.**	δ_C	δ_H, mult (*J* in Hz)	COSY	^{1}H-^{13}C HMBC	ROESY
1	164.4				
2	97.3				
3	164.7				
4	106.4				
5	156.6				
6	29.6	3.34, d *(7.0)*	7, 8-CH_3	4, 5, 7, 8	7, 4- CH_3, 8-CH_3
7	118.6	5.19, t *(7.0)*	6, 8-CH_3	5, 6, 9, 8-CH_3	6, 9
8	136.9				
9	42.2	2.75, d *(6.9)*	10	7, 8, 10, 8-CH_3	7, 10, 11, 8-CH_3
10	124.0	5.45, dt *(15.6, 6.9)*	9, 11	8, 9, 12	9, 11, 12-CH_3
11	136.6	6.02, d *(15.6)*	9, 10	9, 12, 13, 12-CH_3	9, 10, 13, 12-CH_3
12	132.1				
13	135.4	5.29, d *(9.3)*	14, 12-CH_3	11, 14, 15, 12-CH_3, 14-CH_3	11, 14, 15, 14-CH_3
14	36.1	2.53, m	13, 15, 14-CH_3	12, 13, 15, 14-CH_3	15, 12-CH_3, 14-CH_3
15	80.5	3.59, br d *(6.6)*	14, 15-OH	13, 16, 17, 14-CH_3	13, 14, 14-CH_3, 15-OH
16	119.4	5.33, q *(6.6)*	17-CH_3	15, 18, 17-CH_3	15, 17-CH_3
17	137.7				
18	11.3	1.50, s		16, 17	15
3-OH		10.46, s		4	
15-OH		4.46, d *(3.6)*	15		15
2-CH_3	9.2	1.82, s		1, 2, 3	
4-CH_3	9.8	1.88, s	6	3, 4, 5	6
8-CH_3	16.3	1.66, s	6, 7	7, 8, 9	6, 9
12-CH_3	12.7	1.65, s	13	12, 13	10, 11, 14
14-CH_3	17.9	0.76, d *(6.8)*	14	13, 14, 15	13, 14, 15
17-CH_3	12.8	1.54, d (6.7)	16	16, 17	16

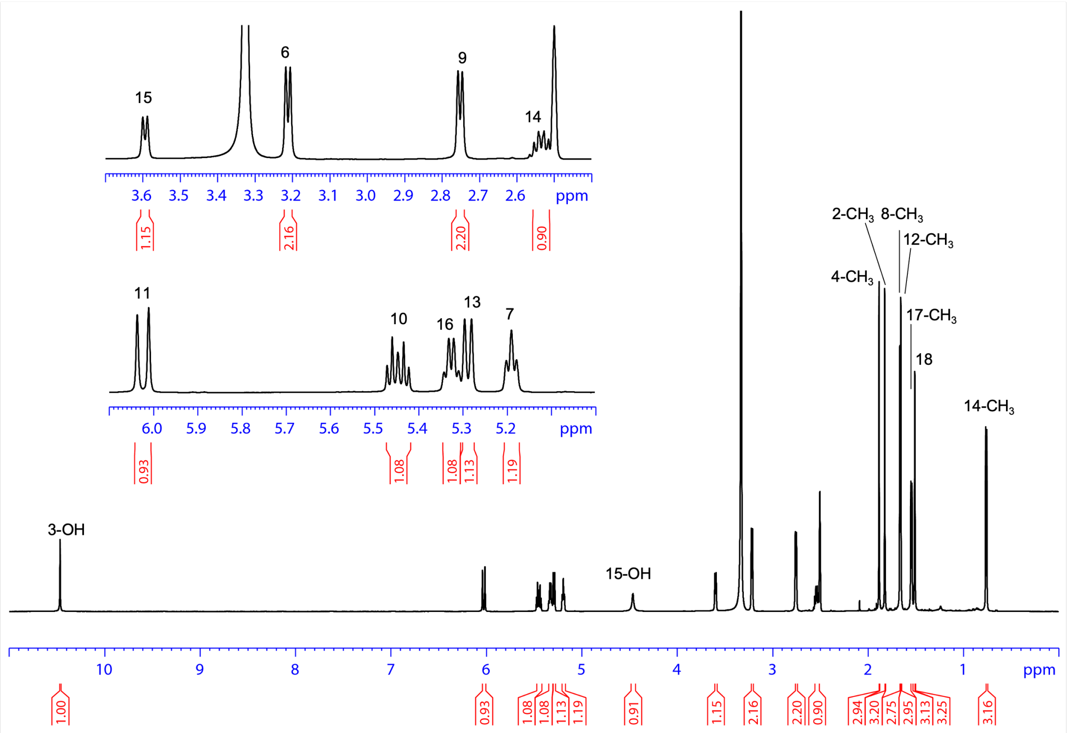

Figure S61. ^{1}H NMR (DMSO-d_6) spectrum of goondapyrone I (**9**).

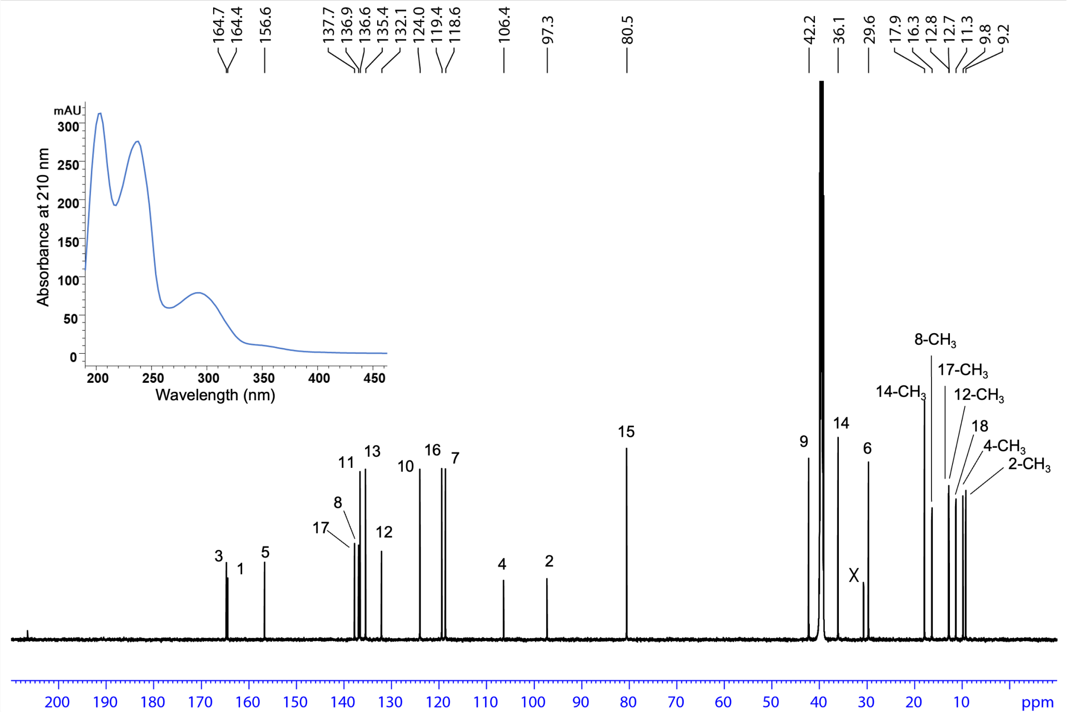

Figure S62. ^{13}C NMR (DMSO-d_6) and UV-vis (inset) spectra of goondapyrone I (**9**).

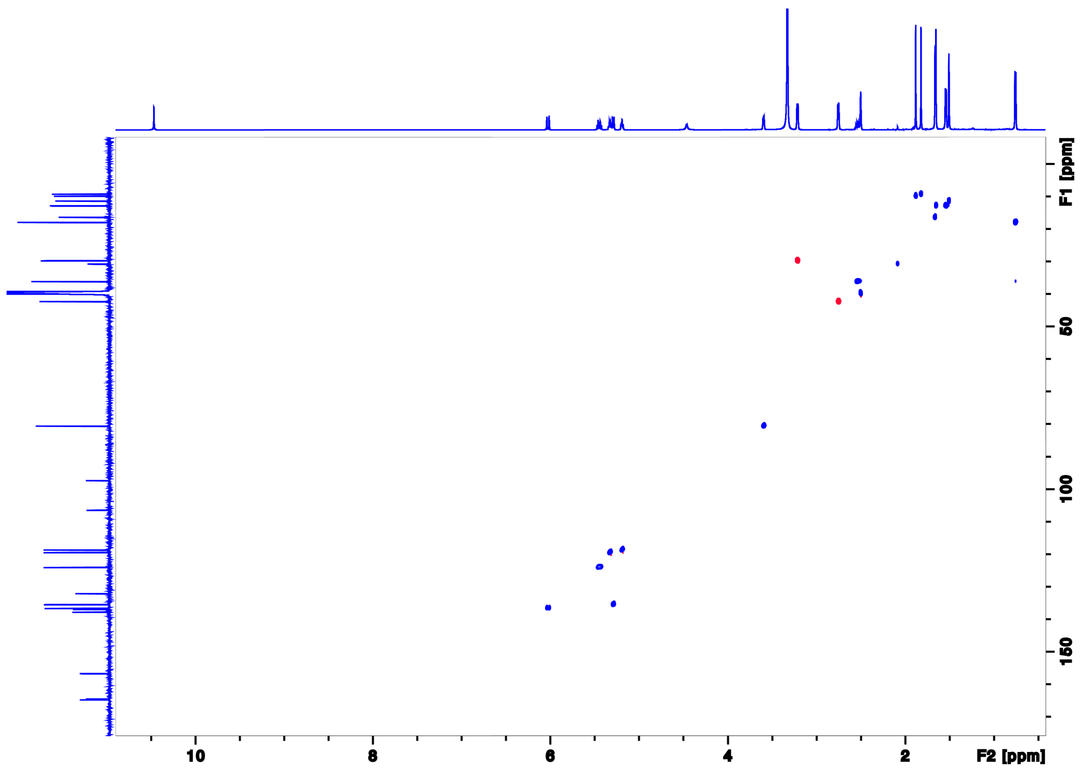

Figure S63. HSQC NMR (DMSO-d_6) spectrum of goondapyrone I (**9**).

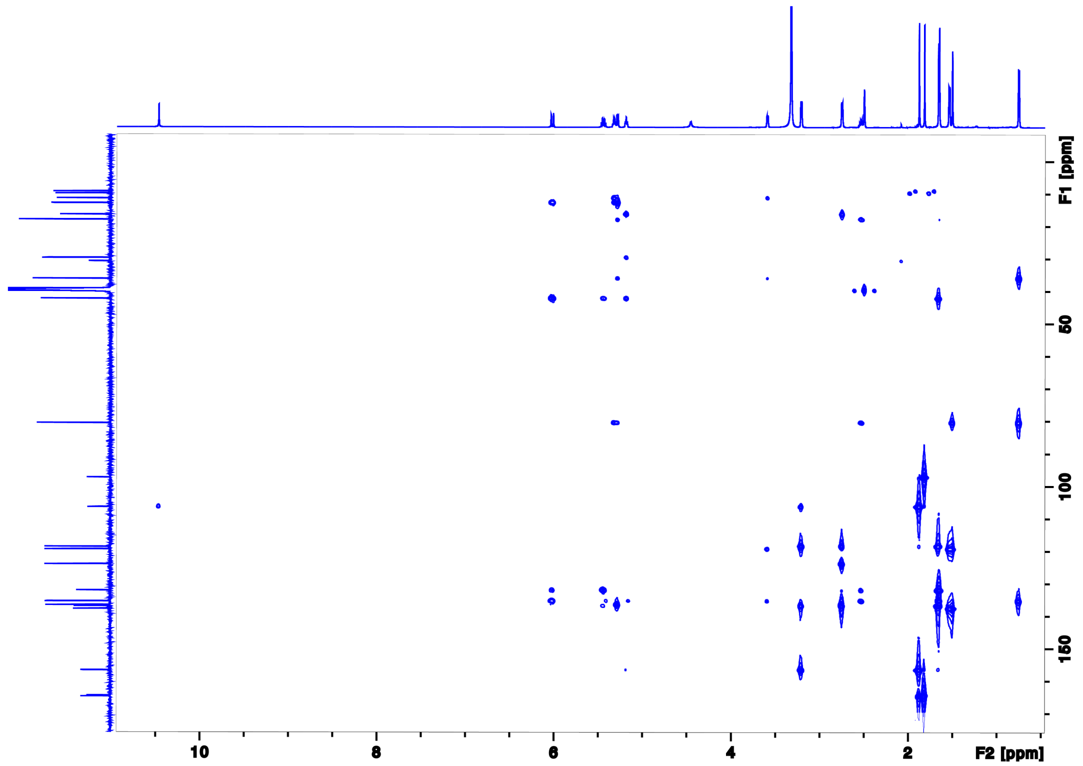

Figure S64. HMBC NMR (DMSO-d_6) spectrum of goondapyrone I (**9**).

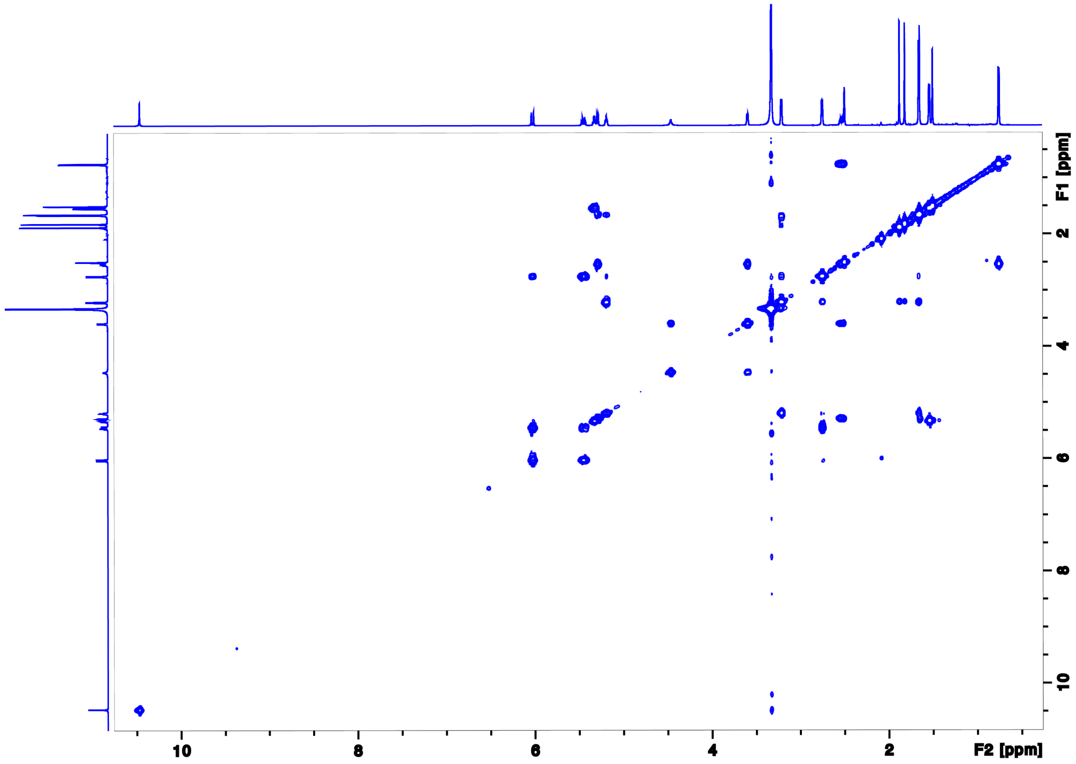

Figure S65. COSY NMR (DMSO-*d*6) spectrum of goondapyrone I (**9**).

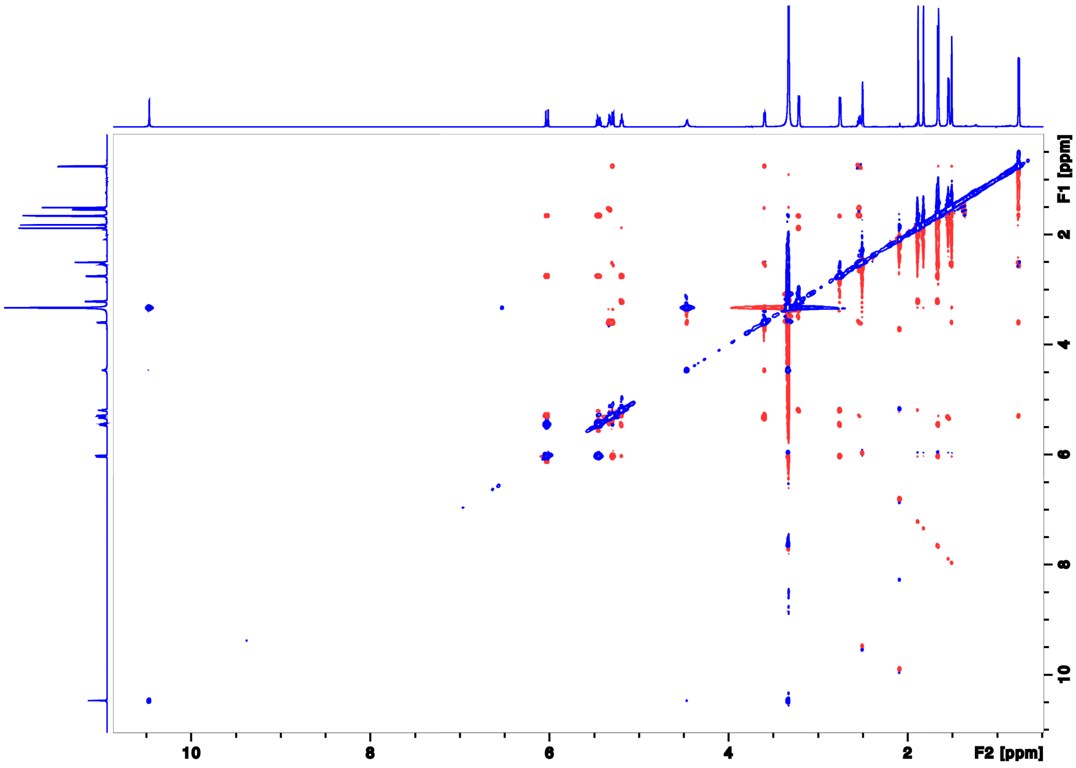

Figure S66. ROESY NMR (DMSO-*d*6) spectrum of goondapyrone I (**9**).

Mass Spectrum Molecular Formula Report

Analysis Info

Analysis Name	D:\Data\Shengbin\Shengbin_S4S196A10_F30-32D_1.d	Acquisition Date	9/7/2022 3:14:46 PM
Method	tune-med_AP.m	Operator	a.salim
Sample Name	Shengbin_S4S196A10_F30-32D_1	Instrument / Ser#	micrOTOF 213750.00232
Comment			

Acquisition Parameter

Source Type	ESI	Ion Polarity	Positive	Set Nebulizer	0.8 Bar
Focus	Not active			Set Dry Heater	180 °C
Scan Begin	100 m/z	Set Capillary	4500 V	Set Dry Gas	5.0 l/min
Scan End	1000 m/z	Set End Plate Offset	-500 V	Set Divert Valve	Source

Generate Molecular Formula Parameter

Formula, min.		
Formula, max.		
Measured m/z	Tolerance	Charge
Check Valence	Minimum	Maximum
Nirogen Rule	Electron Configuration	
Filter H/C Ratio	Minimum	Maximum
Estimate Carbon		

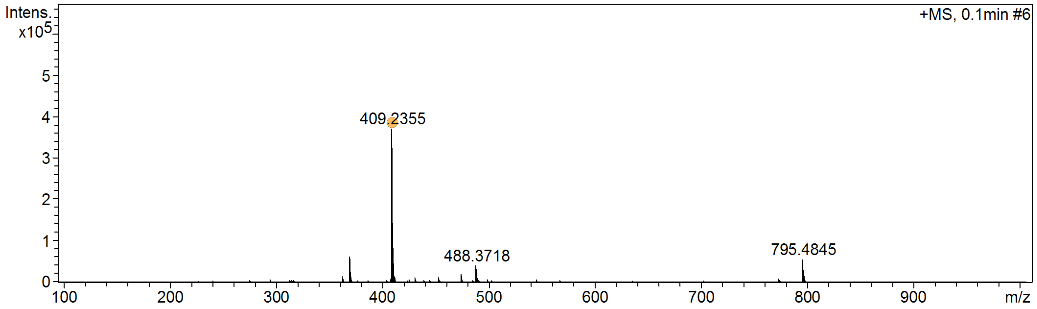

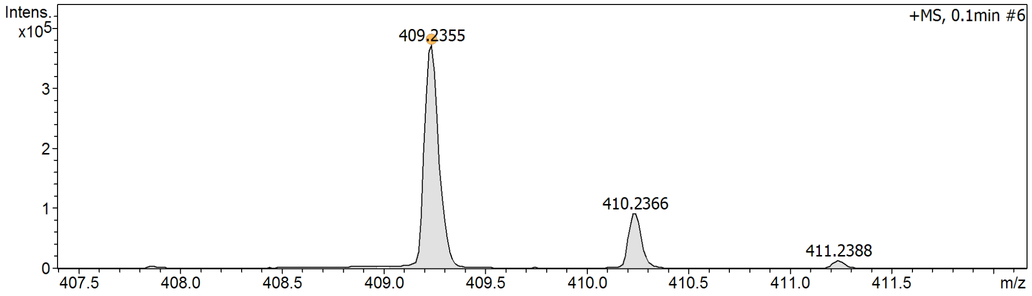

Meas. m/z	#	Ion Formula	m/z	err [ppm]	mSigma	# Sigma	Score	rdb	e⁻ Conf	N-Rule
409.2355	1	C24H34NaO4	409.2349	-1.4	9.6	1	100.00	7.5	even	ok

Bruker Compass DataAnalysis 4.1 printed: 3/27/2023 10:36:50 AM Page 1 of 1

Figure S67. HRESIMS spectrum for goondapyrone I (**9**).

2.10 Goondapyrone J (10)

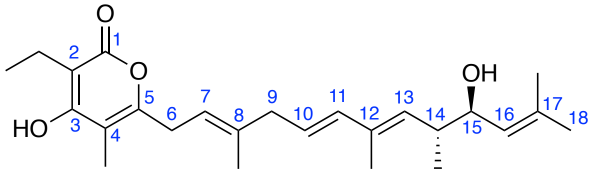

Table S11. 1D and 2D NMR (DMSO-d_6) data for goondapyrone J (**10**).

**Pos.**	δ_C	δ_H, mult (*J* in Hz)	COSY	^{1}H-^{13}C HMBC	ROESY
1	164.0				
2	103.5				
3	164.2				
4	106.4				
5	156.9				
6	29.7	3.21, d *(7.0)*	7, 8-CH_3	4, 5, 7, 8	7, 4-CH_3, 8-CH_3
7	118.6	5.19, t *(7.0)*	6, 8-CH_3	5, 6, 9, 8-CH_3	6, 9
8	136.9				
9	42.2	2.75, d *(6.9)*	10	7, 8, 10, 8-CH_3	7, 10, 11, 8-CH_3
10	124.0	5.45, dt *(15.6, 6.9)*	9, 11	8, 9, 12	9, 11, 12-CH_3
11	136.6	6.02, d *(15.6)*	9, 10	9, 12, 13, 12-CH_3	9, 10, 13
12	132.1				
13	135.4	5.29, d *(9.3)*	14, 12-CH_3	11, 14, 15, 12-CH_3, 14-CH_3	11, 14, 14-CH_3
14	36.1	2.54, m	13, 15, 14-CH_3	12, 13, 15, 14-CH_3	15, 14-CH_3
15	80.5	3.59, br s	14, 15-OH	16	13, 14, 14-CH_3, 15-OH
16	119.4	5.32, q *(6.6)*	17-CH_3	15, 18, 17-CH_3	15, 18, 17-CH_3
17	137.8				
18	11.3	1.50, s		15, 16, 17	14, 15, 14-CH_3
3-OH					
15-OH		4.47, d *(3.9)*	15	14	15
4-CH_3	9.8	1.88, s	6	3, 4, 5	6
8-CH_3	16.3	1.66, s	6, 7	7, 8, 9	6, 9
12-CH_3	12.7	1.65, s	13	12, 13	10, 14
14-CH_3	17.9	0.75, d *(6.7)*	14	13, 14, 15	13, 14, 15
17-CH_3	12.8	1.54, d *(6.6)*	16	16, 17	16
2-$\underline{CH_2}CH_3$	16.5	2.35, q *(7.5)*	2-$CH_2\underline{CH_3}$	1, 2, 3, 2-$CH_2\underline{C}H_3$	2-$CH_2\underline{CH_3}$
2-$CH_2\underline{CH_3}$	12.8	0.93, t *(7.5)*	2-$C\underline{H_2}CH_3$	2, 2-$\underline{C}H_2CH_3$	2-$C\underline{H_2}CH_3$

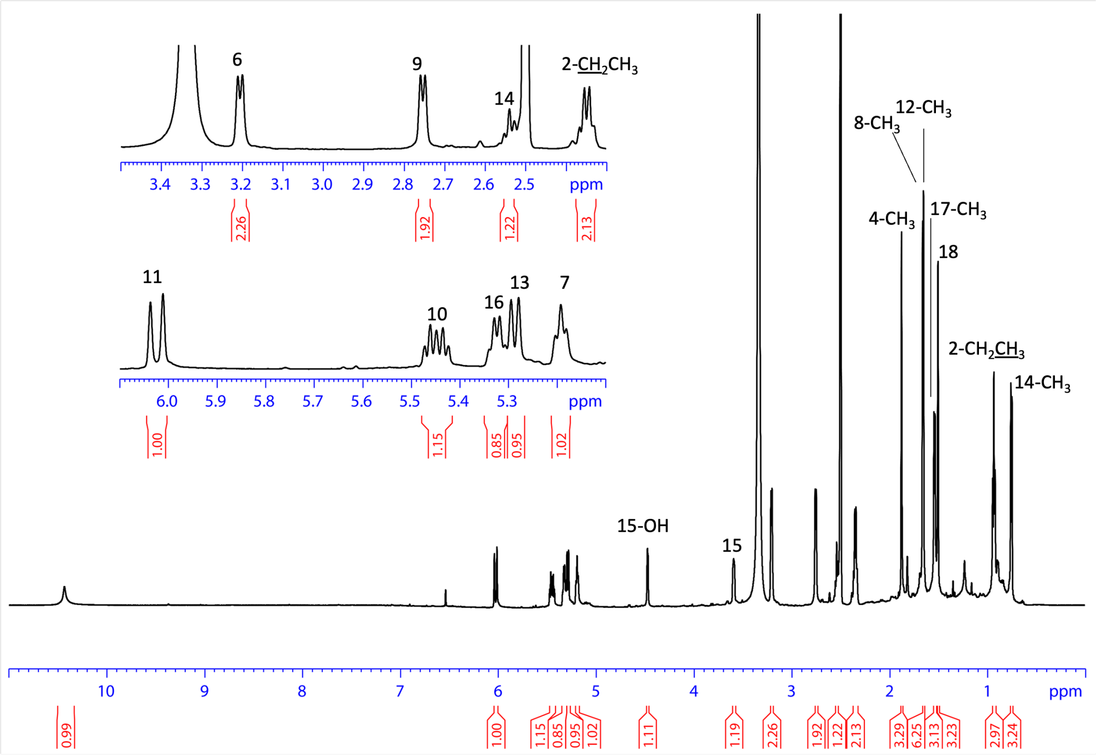

Figure S68. ^{1}H NMR (DMSO-d_6) spectrum of goondapyrone J (**10**).

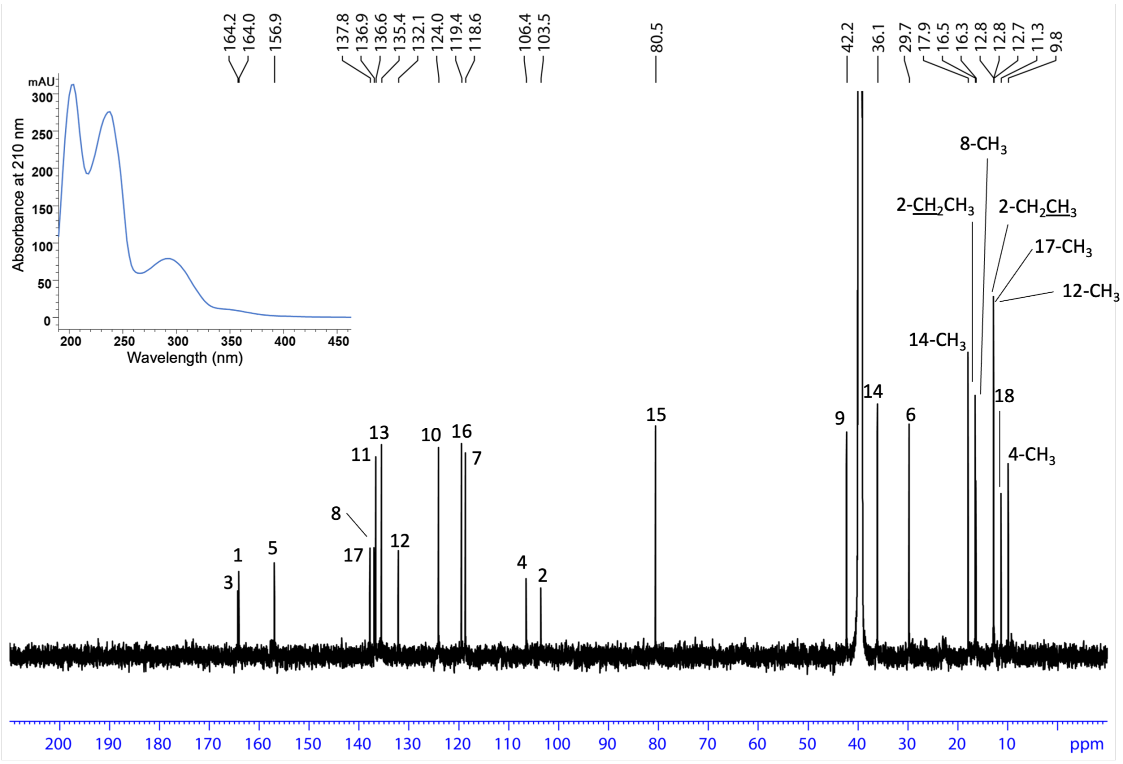

Figure S69. ^{13}C NMR (DMSO-d_6) and UV-vis (inset) spectra of goondapyrone J (**10**).

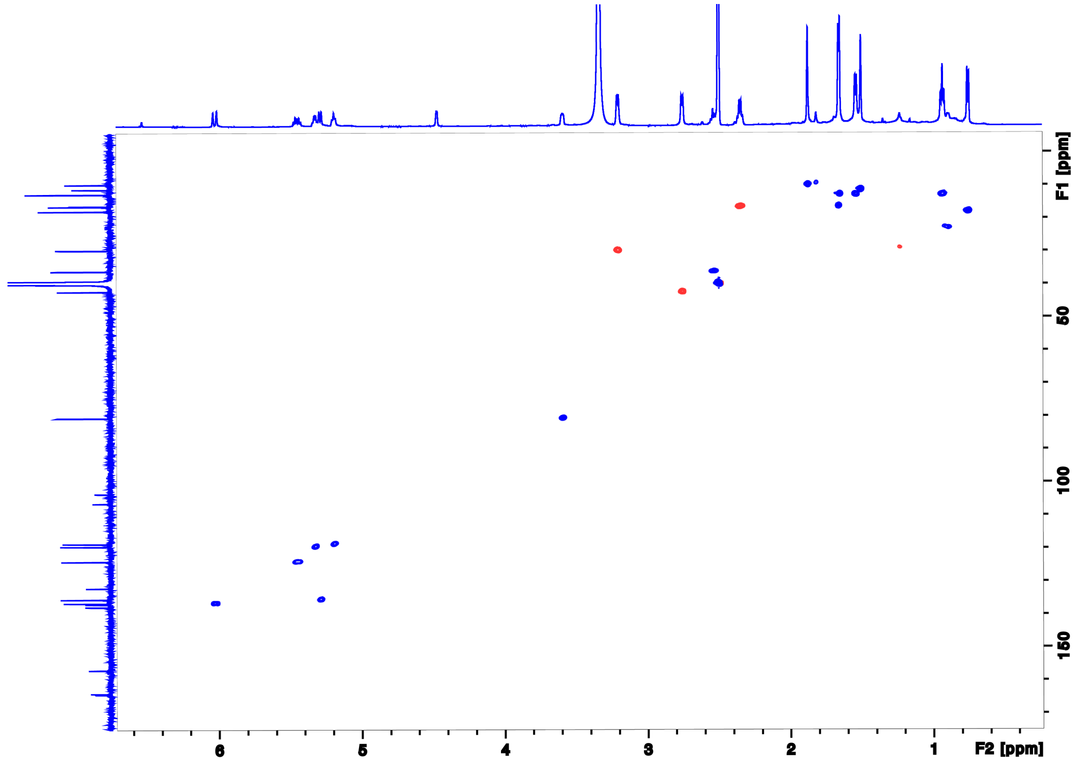

Figure S70. HSQC NMR (DMSO-d_6) spectrum of goondapyrone J (**10**).

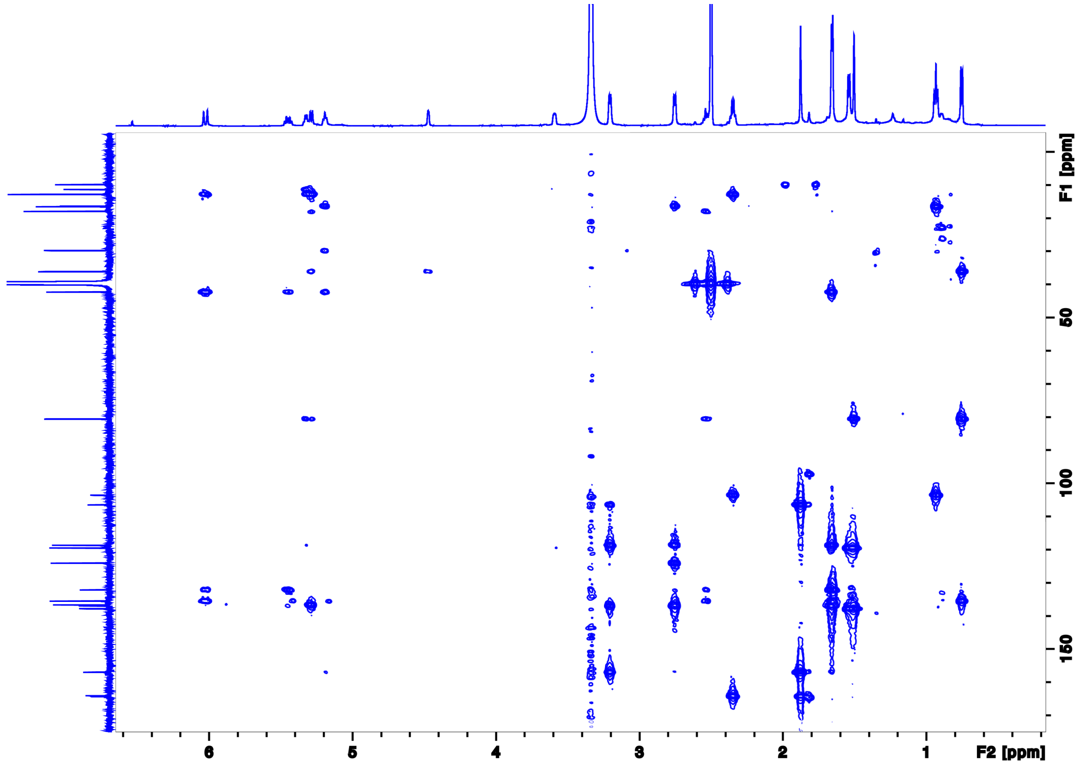

Figure S71. HMBC NMR (DMSO-d_6) spectrum of goondapyrone J (**10**).

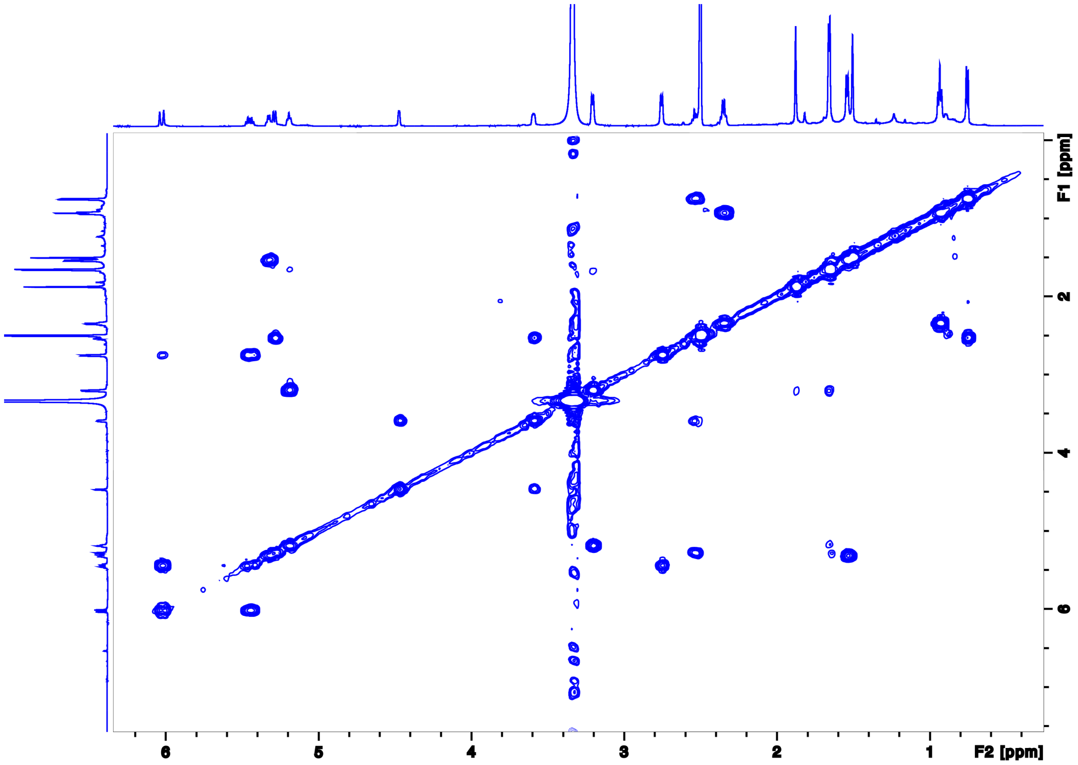

Figure S72. COSY NMR (DMSO-d_6) spectrum of goondapyrone J (**10**).

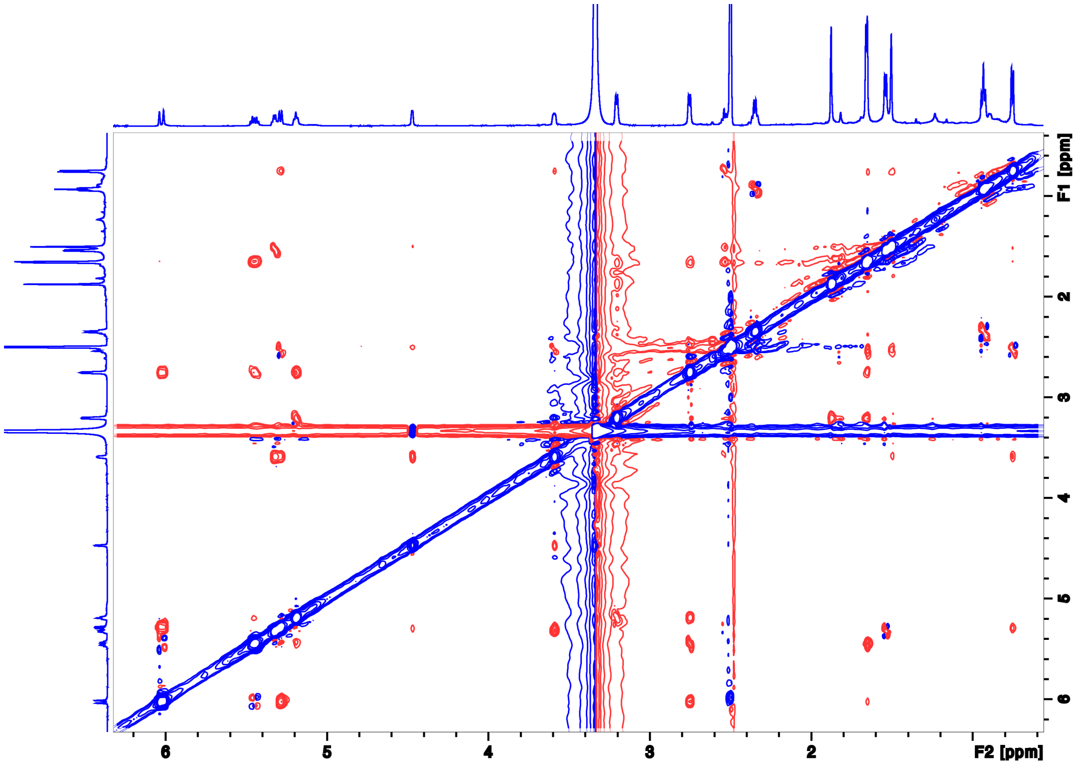

Figure S73. ROESY NMR (DMSO-d_6) spectrum of goondapyrone J (**10**).

Mass Spectrum Molecular Formula Report

Analysis Info

Analysis Name	D:\Data\Shengbin\Shengbin_S4S196A10_F30-32D_2.d	Acquisition Date	9/7/2022 4:52:01 PM
Method	tune-medhigh_AP.m	Operator	a.salim
Sample Name	Shengbin_S4S196A10_F30-32D_2	Instrument / Ser#	micrOTOF 213750.00232
Comment			

Acquisition Parameter

Source Type	ESI	Ion Polarity	Positive	Set Nebulizer	0.5 Bar
Focus	Not active			Set Dry Heater	180 °C
Scan Begin	100 m/z	Set Capillary	4500 V	Set Dry Gas	5.0 l/min
Scan End	1500 m/z	Set End Plate Offset	-500 V	Set Divert Valve	Source

Generate Molecular Formula Parameter

Formula, min.		
Formula, max.		
Measured m/z	Tolerance	Charge
Check Valence	Minimum	Maximum
Nirogen Rule	Electron Configuration	
Filter H/C Ratio	Minimum	Maximum
Estimate Carbon		

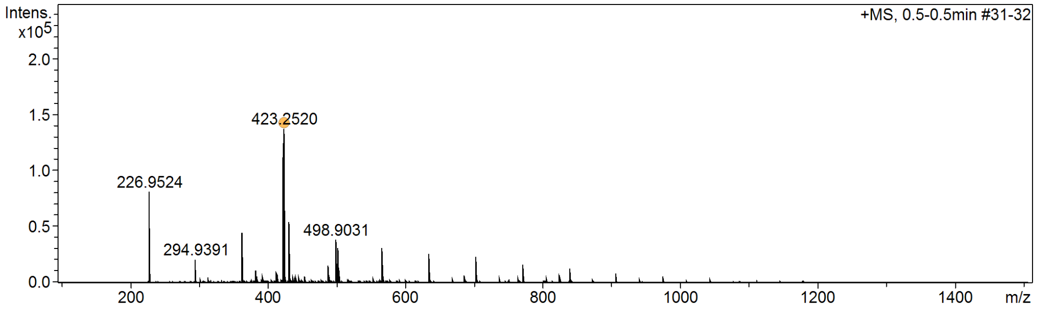

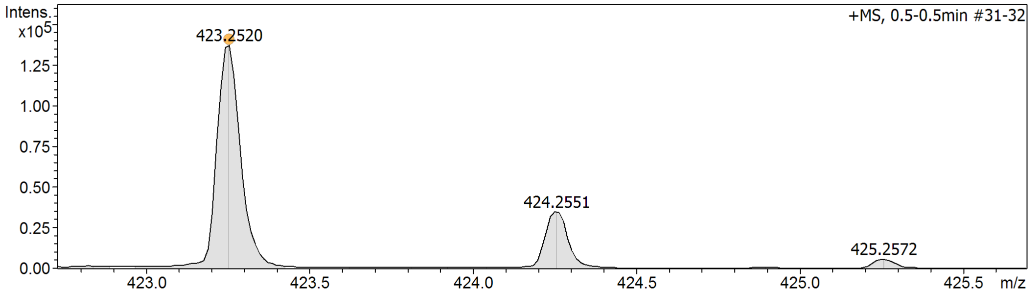

Meas. m/z	#	Ion Formula	m/z	err [ppm]	mSigma	# Sigma	Score	rdb	e¯ Conf	N-Rule
423.2520	1	C25H36NaO4	423.2506	3.4	11.2	1	62.40	7.5	even	ok

Bruker Compass DataAnalysis 4.1 printed: 3/27/2023 10:44:17 AM Page 1 of 1

Figure S74. HRESIMS spectrum for goondapyrone J (**10**).

2.11 Actinopyrone A (11)

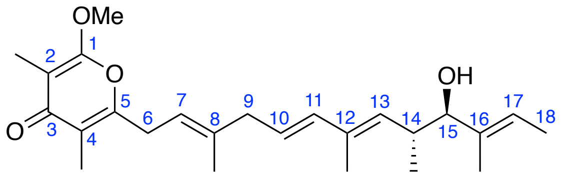

Table S12. ^{1}H NMR ($CDCl_3$) data for actinopyrone A (**11**).

position	**(11)** δ_H, mult (*J* in Hz)	actinopyrone A (lit#) δ_H, mult (*J* in Hz)
6	3.32, d (*7.1*)	3.31, d (*7.3*)
7	5.27, t *(7.1)*	5.26, t *(7.3)*
9	2.81, d *(7.0)*	2.80, d *(6.9)*
10	5.57, dt *(15.6, 7.0)*	5.56, dt *(15.5, 6.9)*
11	6.10, d (*15.6*)	6.10, d (*15.5*)
13	5.25, d *(9.5)*	5.24, d *(9.6)*
14	2.69, ddq *(9.4, 9.3, 6.8)*	2.68, ddq *(9.6, 9.1, 6.9)*
15	3.64, d (*9.2*)	3.63, d (*9.1*)
17	5.50, q (*5.6*)	5.49, q (*6.0*)
18	1.64, d (*5.6*)	1.63, d (*6.0*)
2-CH_3	1.85, s	1.84, s
4-CH_3	1.97, s	1.96, s
8-CH_3	1.81, s	1.81, s
12-CH_3	1.74, s	1.73, s
14-CH_3	0.82, d (*6.8*)	0.81, d (*6.9*)
16-CH_3	1.65, s	1.64, s
1-OCH_3	3.93, s	3.92, s

#Tet Letts, 2006, 47, 5415-5418, synthetic actinopyrone A

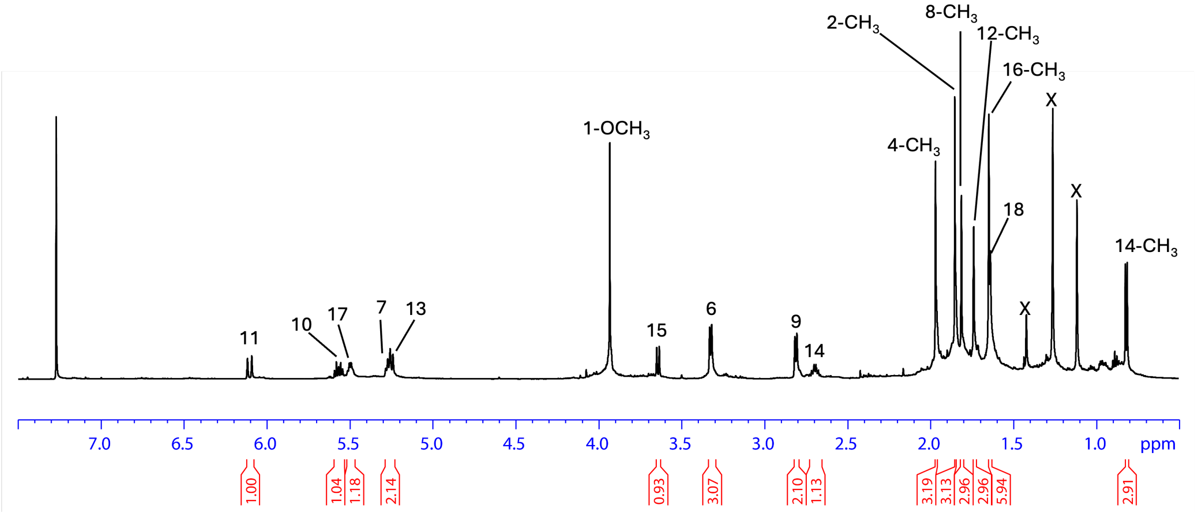

Figure S75. ^{1}H NMR ($CDCl_3$) spectrum of actinopyrone A (**11**).

Table S13. 1D NMR (DMSO-d_6) data for actinopyrone A (**11**)

position	**(11)**	
	δ_C, type	δ_H, mult (*J* in Hz)
1	161.8, C	-
2	97.5, C	-
3	179.4, C	-
4	116.8, C	-
5	157.2, C	-
6	29.4, CH_2	3.35[A], m
7	117.8, CH	5.29, m
8	137.8, C	-
9	42.1, CH_2	2.77, d (*6.9*)
10	124.0, CH	5.47, dt *(15.6, 7.0)*
11	136.6, CH	6.02, d *(15.6)*
12	132.0, C	-
13	135.4, CH	5.27, d *(9.3)*
14	36.0, CH	2.53, m
15	80.5, CH	3.59, d *(7.3)*
16	137.7, C	-
17	119.4, CH	5.33, q *(6.6)*
18	12.8, CH_3	1.54, d (*6.5*)
2-CH_3	6.8, CH_3	1.68, s
4-CH_3	9.5, CH_3	1.83, s
8-CH_3	16.3, CH_3	1.70, br s
12-CH_3	12.7, CH_3	1.65, br s
14-CH_3	17.9, CH_3	0.75, d *(6.8)*
16-CH_3	11.3, CH_3	1.50, br s
1-OCH_3	55.6, CH_3	3.91, s

[A] obscured by solvent, ND not detected

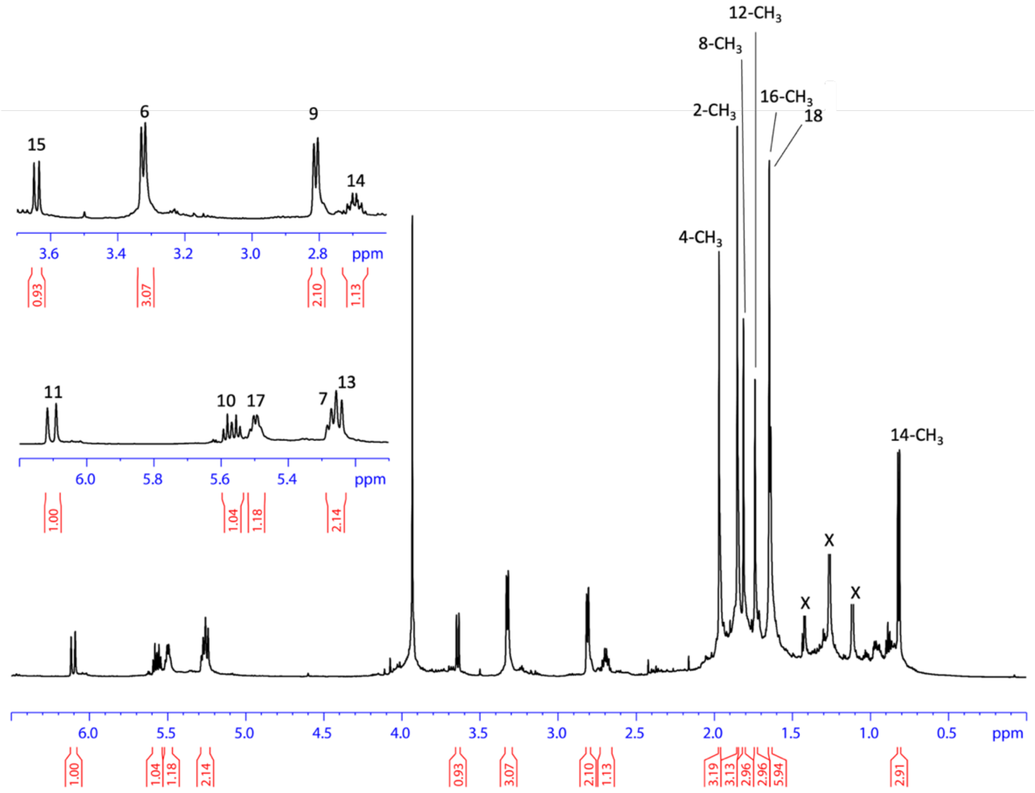

Figure S76. ^{1}H NMR (DMSO-d$_6$) spectrum of actinopyrone A (**11**).

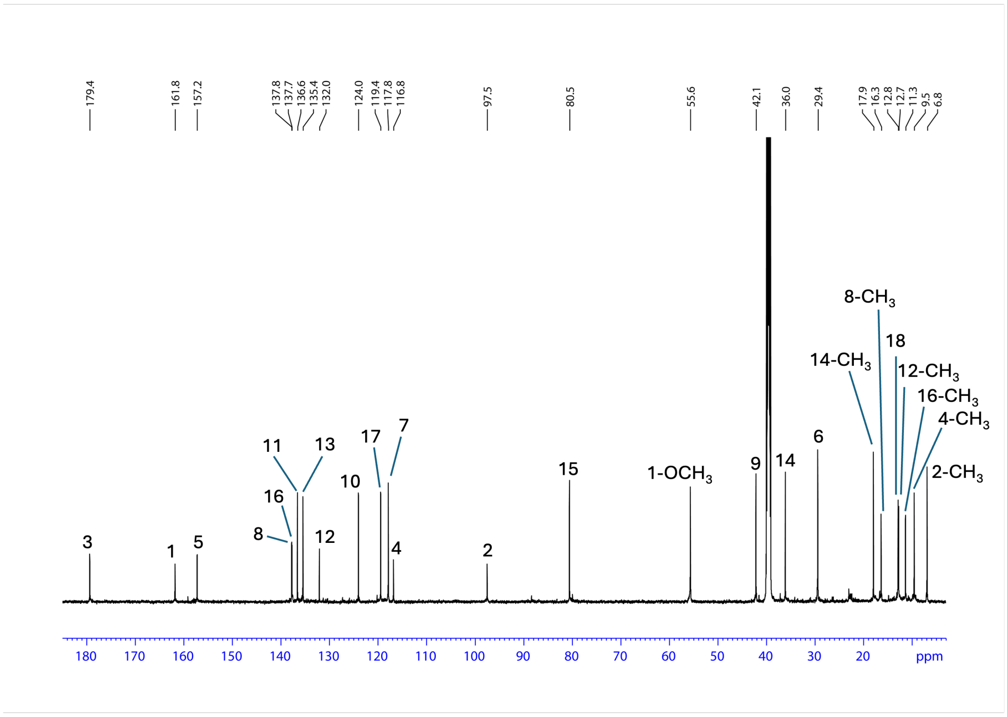

Figure S77. ^{13}C NMR (DMSO-*d*$_6$) spectrum of actinopyrone A (**11**).

2.12 Actinopyrone C (12)

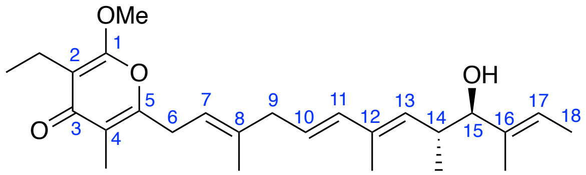

Table S14. 1D NMR (DMSO-d_6) data for actinopyrone A (**11**) and C (**12**).

position	(11) δ_H, mult (J in Hz)	(11) δ_C, type	(12) δ_H, mult (J in Hz)	(12) δ_C, type
1	-	161.8, C	-	161.8, C
2	-	97.5, C	-	103.7, C
3	-	179.4, C	-	178.8, C
4	-	116.8, C	-	117.2, C
5	-	157.2, C	-	157.2, C
6	3.35[A], m	29.4, CH_2	3.35[A], m	29.4, CH_2
7	5.29, m	117.8, CH	5.29, m	117.8, CH
8	-	137.8, C	-	137.8, C
9	2.77, d *(6.9)*	42.1, CH_2	2.77, d *(6.9)*	42.1, CH_2
10	5.47, dt *(15.6, 7.0)*	124.0, CH	5.47, dt *(15.5, 6.9)*	124.0, CH
11	6.02, d *(15.6)*	136.6, CH	6.02, d *(15.5)*	136.6, CH
12	-	132.0, C	-	132.0, C
13	5.27, d *(9.3)*	135.4, CH	5.27, d *(9.5)*	135.4, CH
14	2.53, m	36.0, CH	2.53, m	36.0, CH
15	3.59, d *(7.3)*	80.5, CH	3.59, d *(7.2)*	80.5, CH
16	-	137.7, C	-	137.7, C
17	5.33, q *(6.6)*	119.4, CH	5.33, q *(6.6)*	119.4, CH
18	1.54, d *(6.5)*	12.8, CH_3	1.54, d (*6.6*)	12.8, CH_3
2-CH_3	1.68, s	6.8, CH_3	-	-
2-<u>CH_2</u>CH_3	-	-	2.23, q *(7.4)*	14.8, CH_2
2-CH_2<u>CH_3</u>	-	-	0.91, t *(7.4)*	12.9, CH_3
4-CH_3	1.83, s	9.5, CH_3	1.82, s	9.5, CH_3
8-CH_3	1.70, br s	16.3, CH_3	1.70, br s	16.3, CH_3
12-CH_3	1.65, br s	12.7, CH_3	1.65, br s	12.7, CH_3
14-CH_3	0.75, d *(6.8)*	17.9, CH_3	0.75, d *(6.8)*	17.9, CH_3
16-CH_3	1.50, br s	11.3, CH_3	1.50, br s	11.3, CH_3
1-OCH_3	3.91, s	55.6, CH_3	3.91, s	55.7, CH_3

[A] obscured by solvent, ND not detected

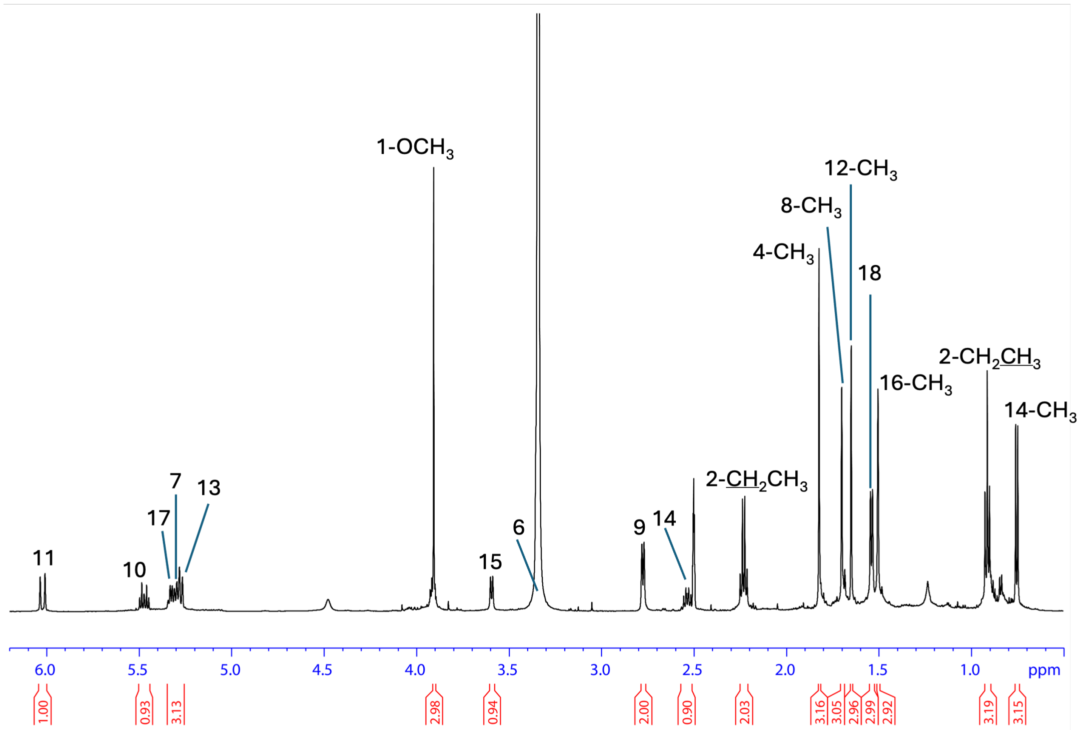

Figure S78. ^{1}H NMR (600 MHz, DMSO-d_6) spectrum of actinopyrone C (**12**).

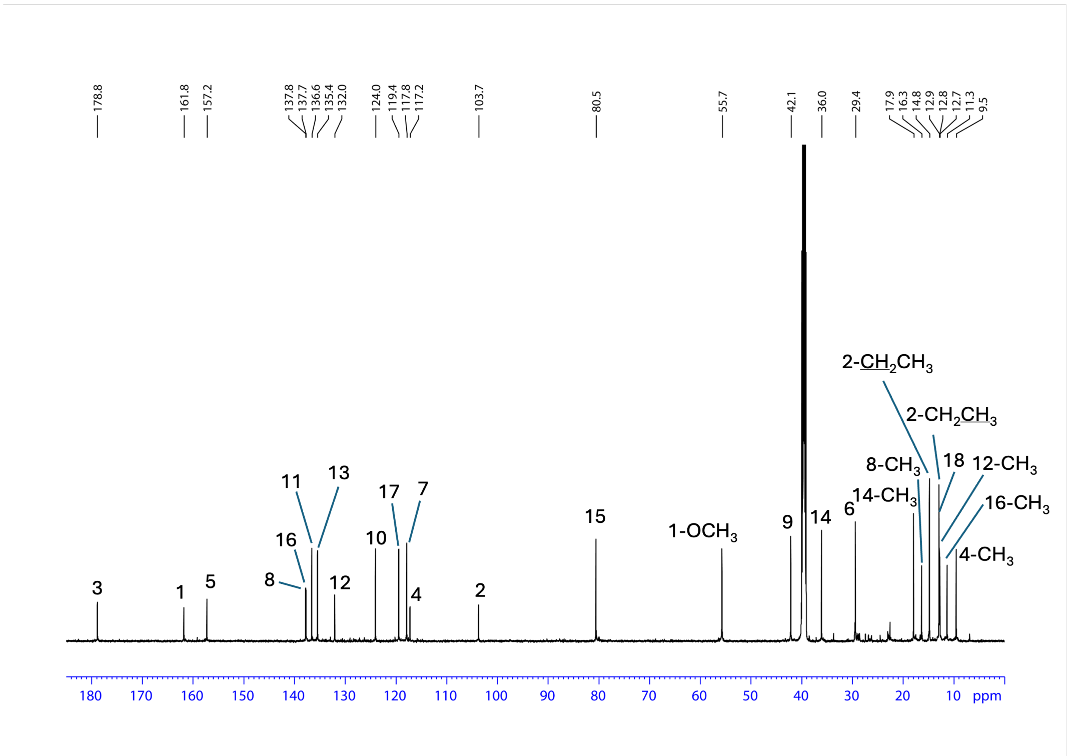

Figure S79. ^{13}C NMR (DMSO-d_6) spectrum of actinopyrone C (**12**).

3. Biological assays

3.1 Antibacterial and Antifungal Assays

Gram-negative *Escherichia coli* ATCC 11775 and Gram-positive *Staphylococcus aureus* ATCC 25923 were streaked on LB agar plates (SDA plates for the fungus *Candida albicans* ATCC 10231) and incubated at 37 °C for 24 h. One bacteria colony was then transferred to fresh LB broth (5 mL) (SD broth for fungi) and incubated at 37°C for 4 h, after which the cell density was adjusted to 10^4–10^5 CFU/mL. Compounds **1–12** were dissolved in DMSO and diluted with H_2O to give a 600 µM stock solution (20% DMSO) which were serially diluted with 20 % DMSO to yield concentrations ranging from 600–0.2 µM. An aliquot (10 µL) of each compound was transferred to a 96-well microtiter plate, and freshly prepared microbial broth (190 µL) was added to each well to give final concentrations of 30–0.01 µM in 1% DMSO. The plates were incubated at 37°C for 24 h for the bacterium and 48 h for the fungus. The optical density of each well was measured spectrophotometrically at 600 nm using a POLARstar Omega plate reader (BMG LABTECH). A mixture of rifampicin and ampicillin (10 µM in 1% DMSO) was used as a positive control for the antibacterial assays, and amphotericin B was used as a positive control (10 µM in 1% DMSO) for the antifungal assay. Each analysis was repeated two times, the data represented graphically, and IC_{50} values calculated using GraphPad Prism version 10.2.0 (Figure S80-S81).

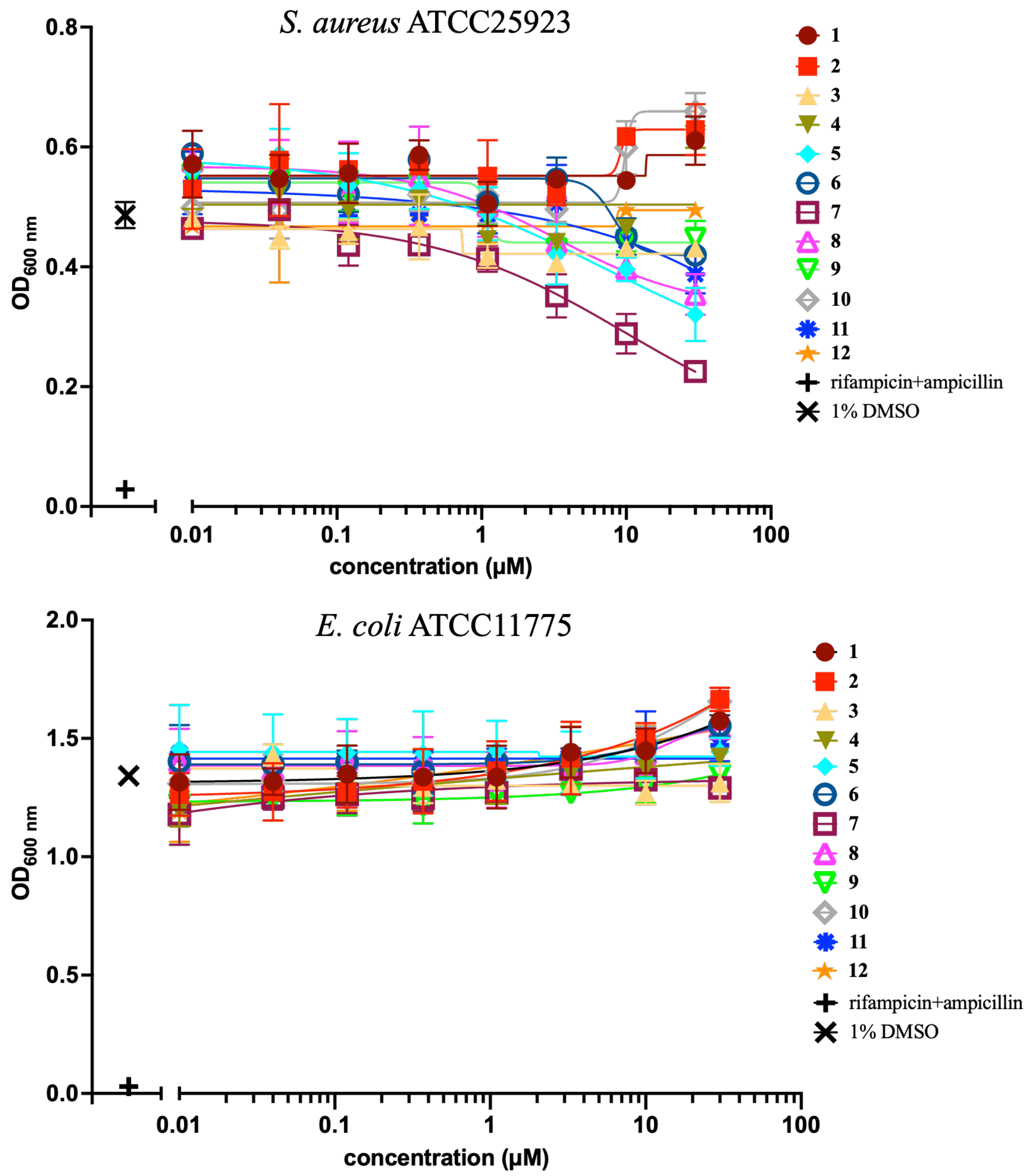

Figure S80. Antibacterial assays for **1-12**.

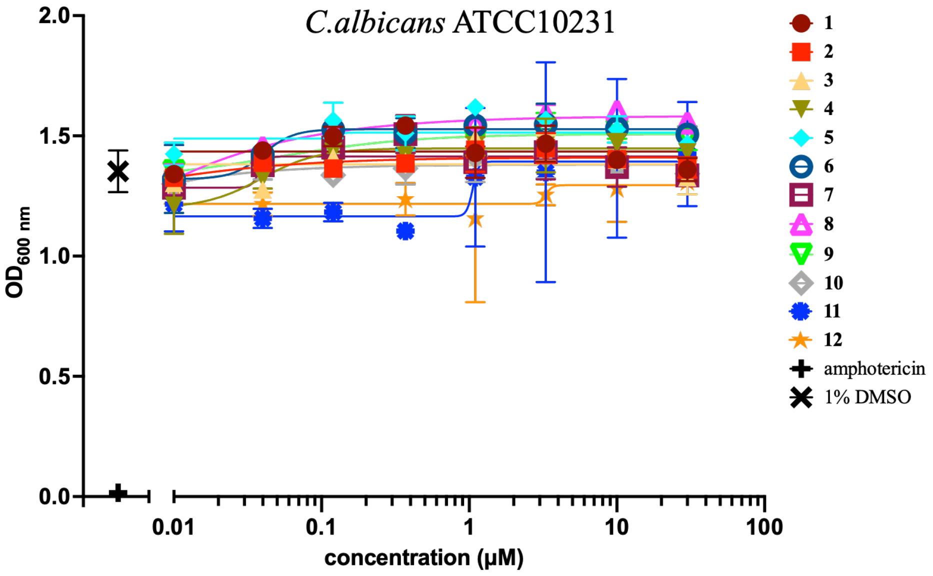

Figure S81. Antifungal assay for **1-12**.

3.2 Cytotoxicity Assay

Human colorectal (SW620) and lung (NCI-H460) carcinoma cells were cultured in Roswell Park Memorial Institute (RPMI) 1640 medium in flasks supplemented with 10% foetal bovine serum, 2 mM L-glutamine, 100 unit/mL penicillin and 100 µg/mL streptomycin in a humidified 37°C incubator supplied with 5% CO_2. Cells were then harvested with trypsin and dispensed into 96-well microtiter assay plates at 2000 cells/well. An aliquot (10 µL) of analytes (**1**–**12**) as prepared above for antibacterial assays were transferred to the 96-well microtiter plate and incubated again for 24 h, after which an aliquot (10 µL) of 3-(4,5-dimethylthiazol-2-yl)-2,5-diphenyltetrazolium bromide (MTT) in sterile water (5 mg/mL) was added to each well, and the microtiter plates were then incubated for a further 3 h at 37°C with 5% CO_2. The medium was aspirated and precipitated formazan crystals were dissolved in DMSO (100 µL/well). The absorbance of each well was measured at 600 nm with a POLARstar Omega plate reader (BMG LABTECH). The negative control was 1% aqueous DMSO, while the positive control was sodium dodecyl sulfate (SDS, 10 mg/mL) for both NCI-H460 and SW620. Each analysis was repeated two times, the data represented graphically, and IC_{50} values calculated using GraphPad Prism version 10.2.0 (Figure S82).

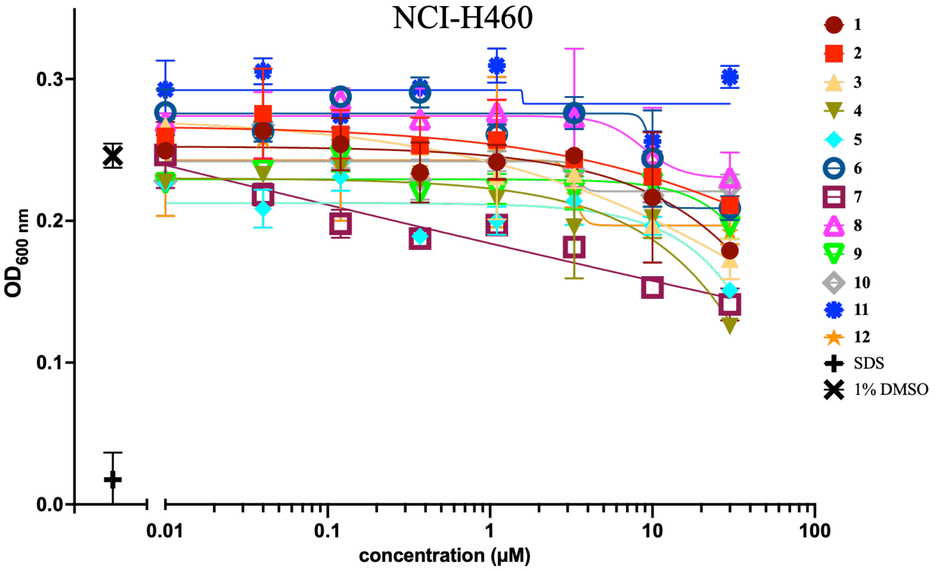

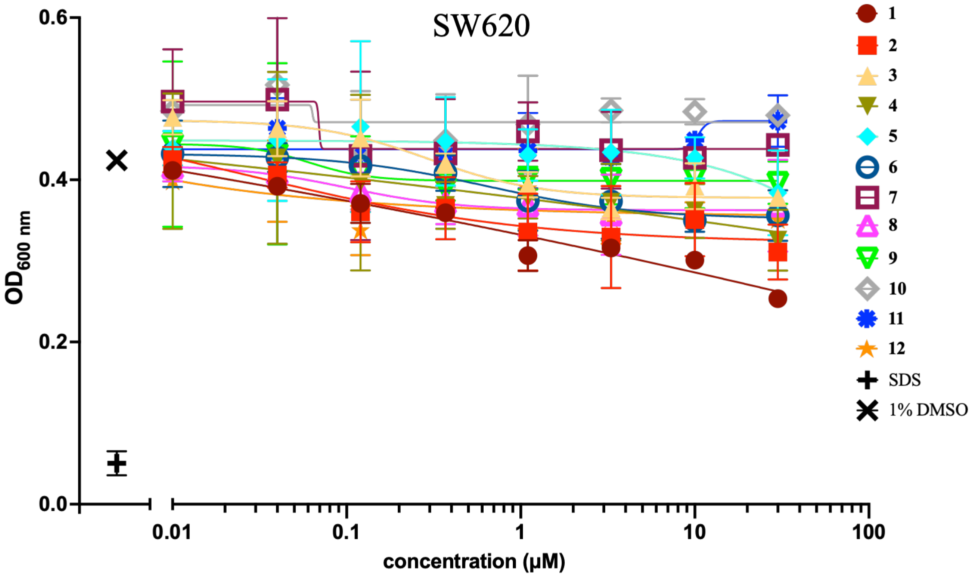

Figure S82. Cytotoxicity assays for **1-12**.

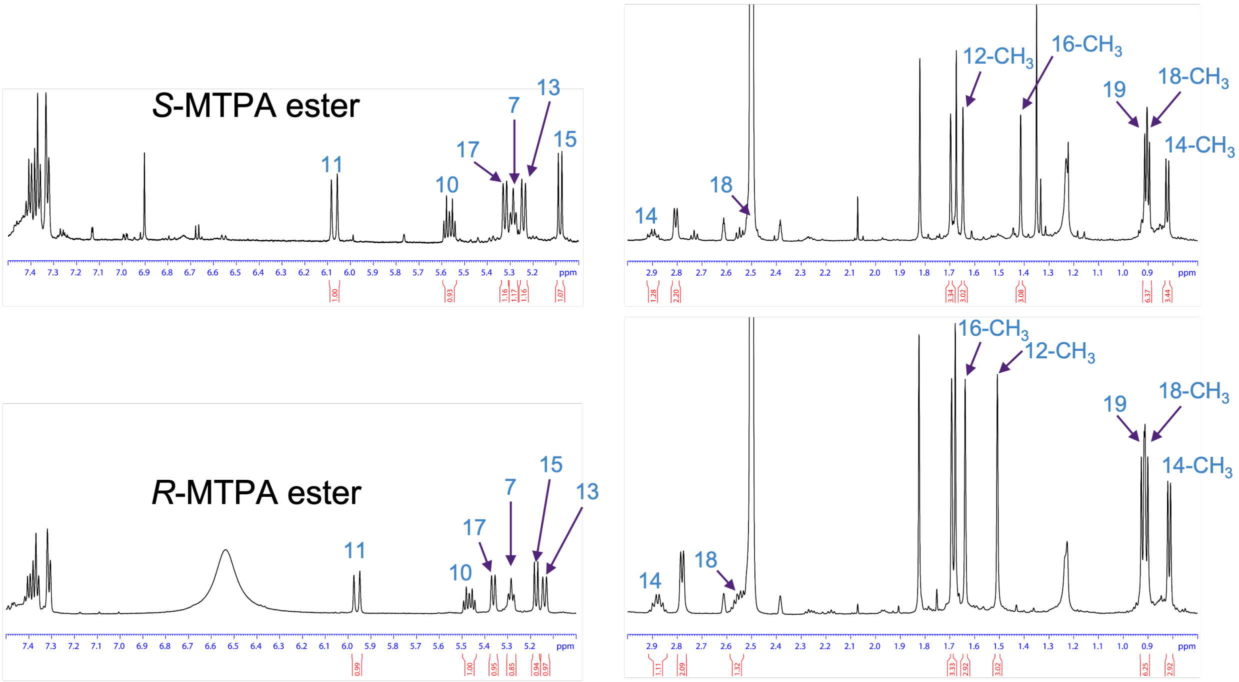

Figure S83. ^{1}H NMR (DMSO-d_6) spectrum of **3a** (*S*-MTPA) and **3b** (*R*-MTPA) esters.

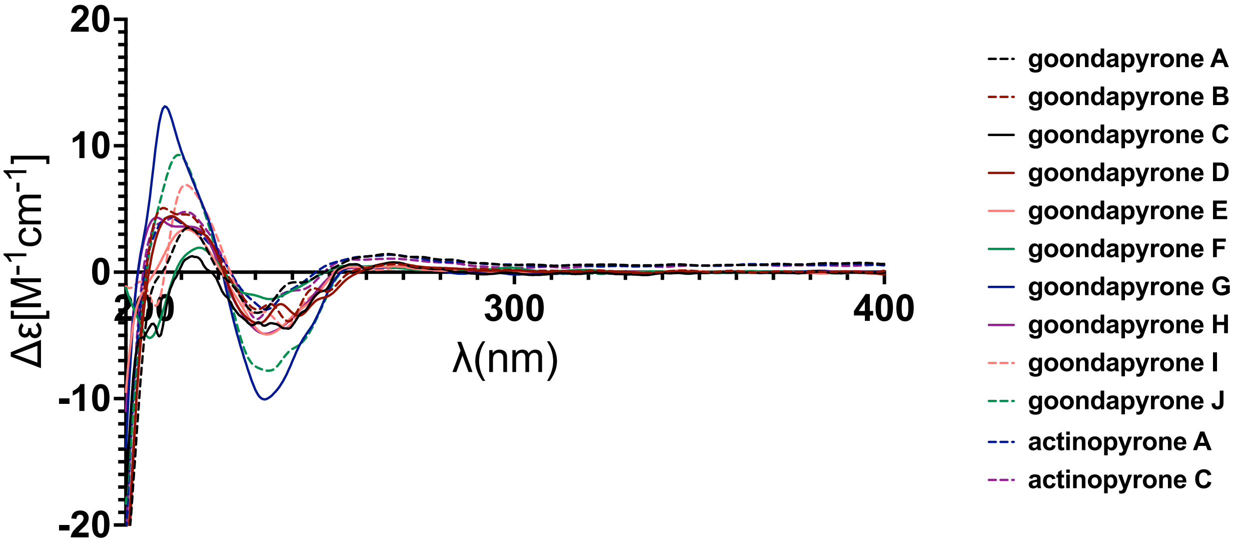

Figure S84. ECD spectra of goondapyrones and actinopyrones.
